# ASD Phenotype—Genotype Associations in Concordant and Discordant Monozygotic and Dizygotic Twins Stratified by Severity of Autistic Traits

**DOI:** 10.3390/ijms20153804

**Published:** 2019-08-03

**Authors:** Valerie W. Hu, Christine A. Devlin, Jessica J. Debski

**Affiliations:** Department of Biochemistry and Molecular Medicine, School of Medicine and Health Sciences, The George Washington University, Washington, DC 20037, USA

**Keywords:** autism, genetics, quantitative traits, stratification by trait severity, heterogeneity reduction, case-control association analysis

## Abstract

Autism spectrum disorder (ASD) is a highly heterogeneous neurodevelopmental disorder characterized by impaired social communication coupled with stereotyped behaviors and restricted interests. Despite the high concordance rate for diagnosis, there is little information on the magnitude of genetic contributions to specific ASD behaviors. Using behavioral/trait severity scores from the Autism Diagnostic Interview-Revised (ADI-R) diagnostic instrument, we compared the phenotypic profiles of mono- and dizygotic twins where both co-twins were diagnosed with ASD or only one twin had a diagnosis. The trait distribution profiles across the respective twin populations were first used for quantitative trait association analyses using publicly available genome-wide genotyping data. Trait-associated single nucleotide polymorphisms (SNPs) were then used for case-control association analyses, in which cases were defined as individuals in the lowest (Q1) and highest (Q4) quartiles of the severity distribution curves for each trait. While all of the ASD-diagnosed twins exhibited similar trait severity profiles, the non-autistic dizygotic twins exhibited significantly lower ADI-R item scores than the non-autistic monozygotic twins. Case-control association analyses of twins stratified by trait severity revealed statistically significant SNPs with odds ratios that clearly distinguished individuals in Q4 from those in Q1. While the level of shared genomic variation is a strong determinant of the severity of autistic traits in the discordant non-autistic twins, the similarity of trait profiles in the concordantly autistic dizygotic twins also suggests a role for environmental influences. Stratification of cases by trait severity resulted in the identification of statistically significant SNPs located near genes over-represented within autism gene datasets.

## 1. Introduction

Autism spectrum disorder (ASD) is a highly heritable and heterogeneous neurodevelopmental disorder which is characterized by deficits in social communication and reciprocal interactions as well as by stereotyped behavior and restricted interests, often accompanied by language difficulties, especially in the area of pragmatics [1]. ASD is often comorbid with disorders such as anxiety, attention-deficit/hyperactivity disorder, and intellectual disability, and some affected individuals also suffer from gastrointestinal, immune system, and sleep disorders, suggestive of systemic dysregulation. Severity levels of autism vary broadly with some cases having only mild deficits in social interactions, while other cases exhibit severe deficits in social behaviors and manifest noticeably aberrant stereotypic behaviors, including self-injury. Boys are four times more likely than girls to have an ASD diagnosis and treatment options are limited in part due to the heterogeneity of the disorder as well as a lack of knowledge of the underlying biology [2].

Ever since the twin study of Bailey et al. [3] reported a concordance rate for autism of 60% for genetically identical monozygotic (MZ) twins and 0% for dizygotic (DZ) twins, autism has been perceived as a strongly genetic disorder. However, a review of twin studies conducted between 1977 and 2011 suggests that concordance rates for autism are quite variable ranging from 36% to 96% for MZ twins, depending in part on sex as well as whether or not the diagnosis was narrowly defined (i.e., strict autism) or more broadly defined as autism spectrum disorder (ASD) [4]. The differences in concordance rates and the incomplete penetrance have been attributed to undefined environmental factors. To tease out the contributions of genetics and environment, there have been increased efforts to assemble large twin cohorts not only for genetic analyses but also for studies on the associated phenotypes of autism, including neurodevelopmental differences [5,6,7,8]. In fact, heritability rates of ASD vary greatly in different studies conducted since 2011, with Hallmayer et al. reporting a moderate genetic effect of 38% [9], Gaugler et al. reporting a narrow-sense (strictly defined) autism heritability of 52% [10], and Tick et al. estimating heritability at 64–91% in a meta-analysis of twin studies [11], with each study acknowledging the effects of shared environment to explain the non-genetic component of ASD. Heritability studies that included subclinical (i.e., broad autism phenotype) diagnoses also indicate that heritability is determined by both genetics and non-shared environment, but that concordance rates are somewhat dependent on diagnostic method [12]. Using three different diagnostic methods, Colvert et al. showed that concordance rates among monozygotic twins ranged from 62% to 75%, while the rate varied from 5% to 40% among dizygotic twins. More recently, several studies using monozygotic and dizygotic twins have investigated the association between autistic traits and specific phenotypes, such as atypical sensory reactivity [13], face identify recognition [14], and social cognition [15], with all of these studies reinforcing genetic links to these phenotypes. However, few studies have used a broad range of specific phenotypic differences among clinically discordant non-autistic twins to determine how zygosity influences the severity of autistic traits.

While twin studies make it apparent that genetics plays a significant role in ASD and associated phenotypes, no one genetic profile emerges due to the heterogeneity of ASD. In order to overcome this issue, some groups have attempted to reduce the heterogeneity by clustering individuals according to clinical, behavioral, or molecular subphenotypes. Previously, we used cluster analyses of severity scores on a broad spectrum of traits and behaviors probed by the Autism Diagnostic Interview-Revised (ADI-R) instrument [16] to identify four phenotypic subgroups of individuals which were qualitatively described as severely language-impaired, intermediate, mild, and savant [17]. In addition, transcriptomic analyses of lymphoblastoid cell lines derived from individuals in three of the four subgroups showed that the ASD subgroups could be distinguished from a group of non-autistic controls as well as from each other, suggesting the contribution of different biological processes to specific subphenotypes of ASD [18]. Furthermore, case-control association analyses based upon the four ASD phenotypic subgroups using SNPs derived from quantitative trait association analyses which were based on ADI-R scores for multiple traits identified novel and statistically significant SNPs that were subtype-dependent [19]. Similarly, a linkage analysis based on these subphenotypes revealed novel genetic loci with highly significant LOD scores that were not detected in the absence of subtyping in addition to intra-family phenotypic and genetic heterogeneity [20]. Another study used a cohort of ASD individuals with extreme sleep onset delay as a means to decrease heterogeneity of cases to tease out significant variants in genes for melatonin pathway enzymes [21]. Recently, subgrouping of females with autism using X chromosome inactivation status as an epigenetic marker revealed a novel alternatively-spliced isoform of an X-linked chromatin gene, KDM5C, which contained the 3′UTR from a retrotransposed gene and was differentially expressed in this subgroup of autistic females relative to controls [22]. Collectively, these studies based on reducing heterogeneity among cases suggest the potential for relating genotype as well as gene structure with specific subphenotypes of ASD. However, there is little information on the relationship between genotype and the specific traits or subphenotypes observed in ASD.

The goals of this study were to: (1) investigate how genetics influences specific autism-associated behaviors and traits among monozygotic versus dizygotic twins, either concordant or discordant for autism diagnosis; (2) utilize ASD trait distribution profiles based on individual ADI-R scores in quantitative trait association analyses to identify single nucleotide polymorphisms (SNPs or quantitative trait nucleotides [QTNs]) that associate with specific traits; (3) determine whether the QTNs can differentiate cases from controls using individuals with ASD at the extremes of each trait distribution profile to reduce the phenotypic heterogeneity of cases for case-control association analyses; and (4) identify genes, pathways, and biological functions associated with specific traits. The results of these deep phenotyping analyses suggest that while shared genotype is a strong determinant of the severity of autistic traits in discordant monozygotic twins, the similarity of trait profiles of genetically distinct dizygotic twins concordant for diagnosis of ASD also suggests a role for shared environment. Moreover, genetic analyses which utilize both quantitative trait and case-control association analyses in which clinical or phenotypic heterogeneity is reduced by limiting the cases to those at the extremes of trait severities reveal highly significant SNPs associated with genes enriched in autism datasets and over-represented in neurological functions relevant to ASD.

## 2. Results

### 2.1. Deep Phenotypic Analyses of MZ and DZ Twins: Influence of Zygosity and Diagnostic Concordance

**Figure 1:**
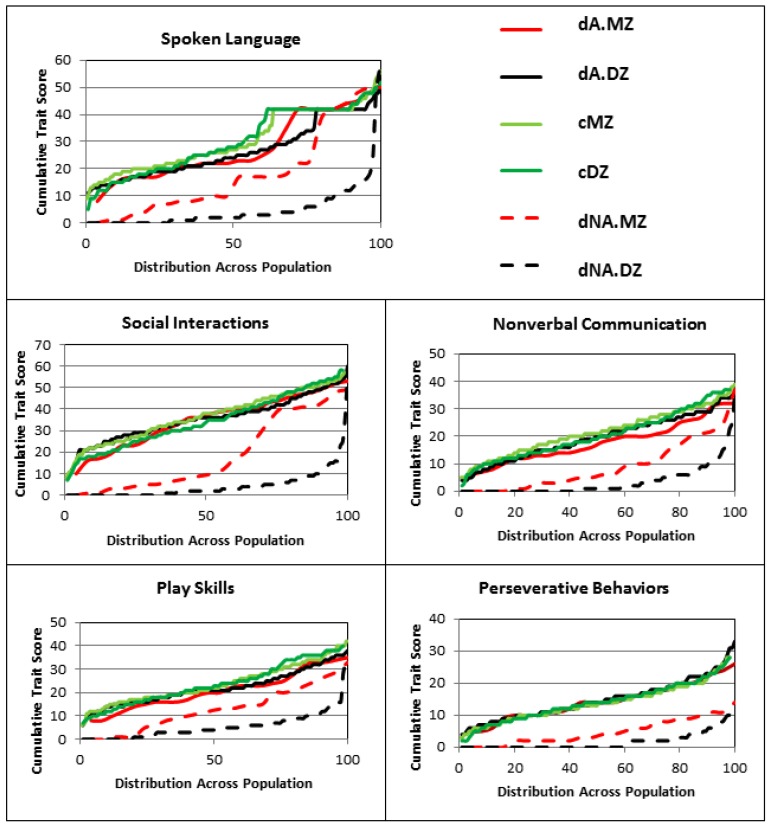
Phenotypic (trait) profiles based on cumulative ADI-R scores for spoken language, nonverbal communication, play skills, social skills/interactions, and perseverative behaviors. In order to better compare the distribution of traits in each twin subgroup, the total number of individuals comprising each group was normalized to 100. The concordant autistic monozygotic (MZ) and dizygotic (DZ) twin subgroups are identified as cMZ and cDZ, respectively, and the discordant autistic (dA) and non-autistic (dNA) MZ and DZ twin subgroups are referred to as dA.MZ, dA.DZ, dNA.MZ, and dNA.DZ, respectively.

A total of 284 pairs of twins represented in the Autism Genetic Resource Exchange (AGRE) were included in this study. The twins were sorted into subgroups based on validated zygosity (monozygotic versus dizygotic) as well as by diagnostic concordance (concordant or discordant) for ASD. Each individual’s ADI-R scores for 88 items covering five ASD traits (spoken language, nonverbal communication, play skills, social interactions, and perseverative behaviors) were downloaded from AGRE and were used to establish trait severity distribution profiles for each subgroup of twins (Figure 1, see legend for nomenclature used for different twin subgroups). Among discordant twins who do not meet the ADI-R diagnostic criteria for autism (i.e., non-autistic co-twins), the trait distribution profiles show noticeably lower severity, as indicated by lower cumulative trait scores, among the DZ twins in comparison to the MZ twins. On the other hand, the discordant MZ and DZ twins who received an autism diagnosis exhibited similar severity profiles across each trait which closely tracked the severity profiles of both MZ and DZ twins who were concordant for autism diagnosis.

**Figure 2:**
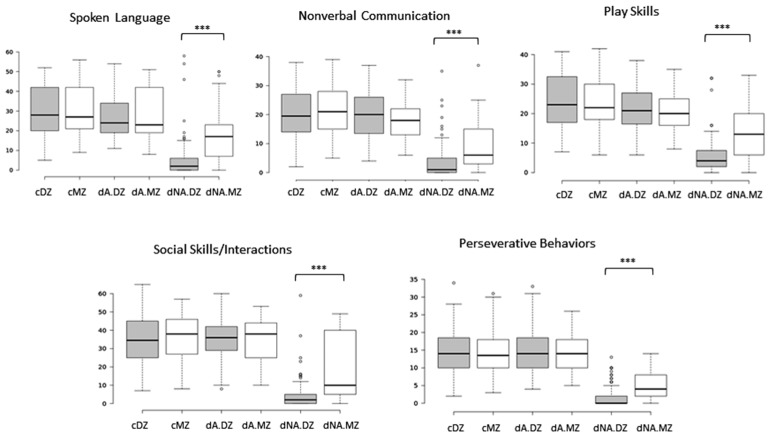
Boxplots showing range of trait scores of all twin groups. Student’s *t*-test was used to show that there were significant differences between the trait scores for dNA.MZ and dNA.DZ twins. *** *p*-value ≤ 0.001.

**Figure 3:**
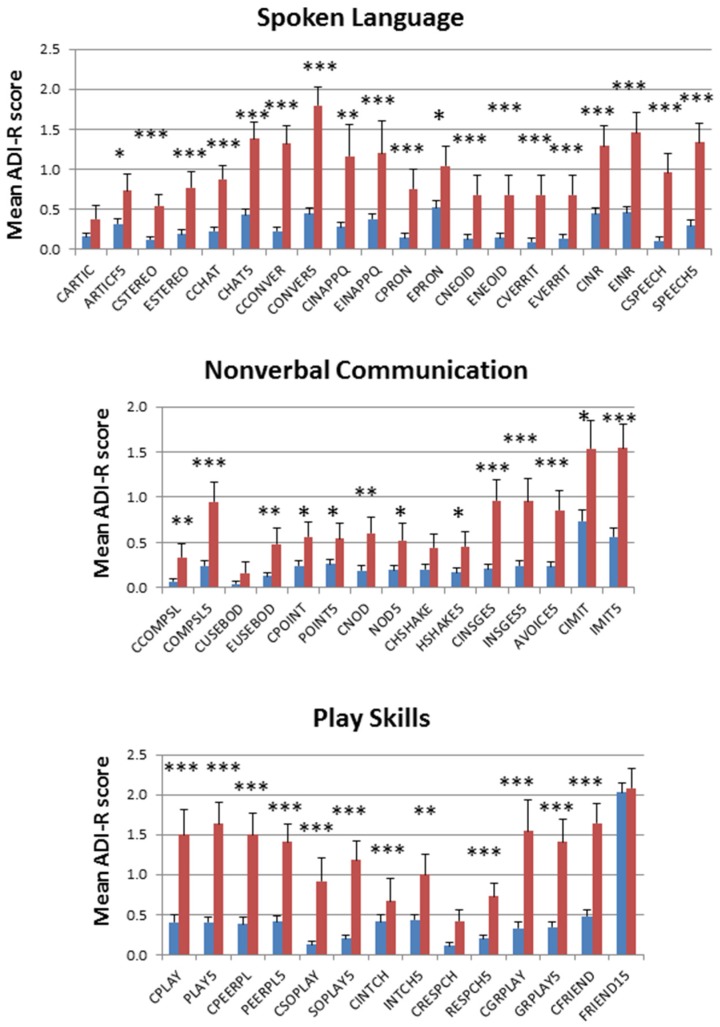

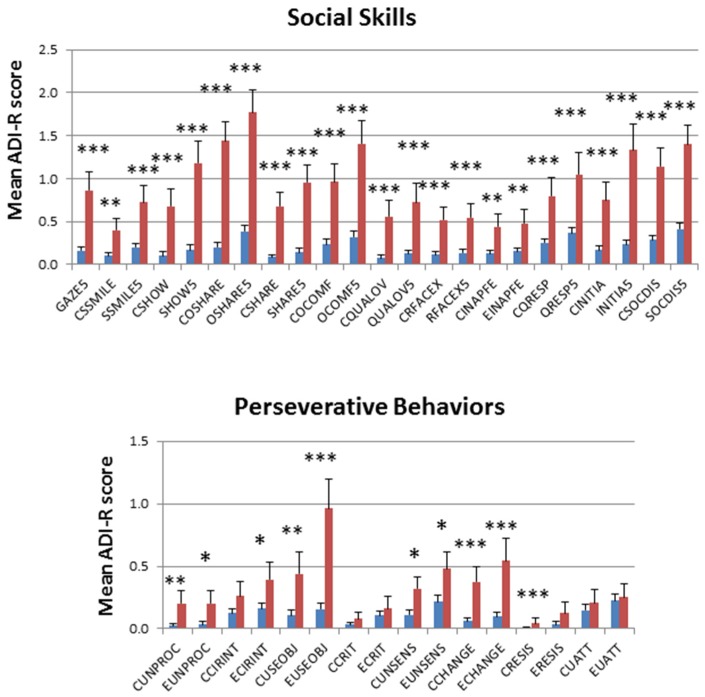
Comparison of average severity scores for dNA.MZ (red bars) and dNA.DZ (blue bars) twins across all ADI-R items (along horizontal axes) used to derive a cumulative trait score. * *p*-value ≤ 0.05, ** *p*-value ≤ 0.01, *** *p*-value ≤ 0.001.

Boxplots of the cumulative trait scores for each trait exhibit the same pattern of severity where the non-autistic DZ twins have a significantly lower average severity for each trait in comparison to the non-autistic MZ twins, but all ASD-diagnosed twins exhibit similar average trait severity scores, regardless of zygosity or concordance (Figure 2). The comparisons of average severity scores for all ADI-R items that comprise each trait also reveal significant differences (*p*-values ≤ 0.05) between the non-autistic MZ and DZ twins for a majority of the items (Figure 3). Taken together, the significantly higher trait severity of non-autistic MZ twins in comparison to non-autistic DZ twins supports the notion that genetics is a strong determinant of the severity of virtually all autistic traits and behaviors, even in undiagnosed individuals. On the other hand, the almost identical severity profiles of the concordantly autistic but genetically different DZ co-twins suggest that shared environment may also contribute to trait severity. However, the specific relationships among genotype, environmental factors, and ASD phenotypes remain unknown.

### 2.2. Genotype–Phenotype Relationships Revealed by Quantitative Trait Association Analyses

**Scheme 1:**
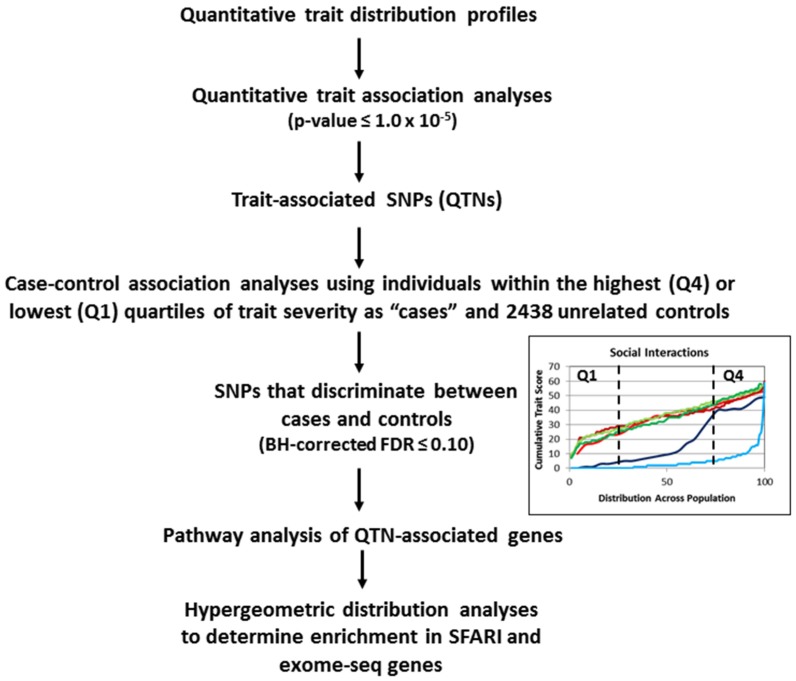
Workflow for two-phase genetic analyses using both quantitative trait and case-control association analyses of individuals stratified according to severity of traits.

**Figure 4:**
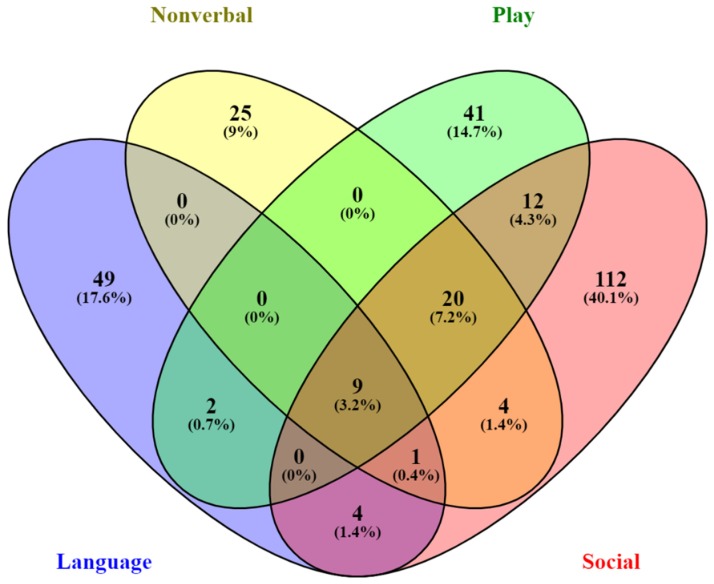
Venn diagram showing SNP-associated gene overlap among language, nonverbal communication, play skills, and social skills. There was no gene overlap between the genes associated with perseverative behaviors and those associated with any of the other four traits.

Scheme 1 illustrates the overall design and workflow for the genetic analyses conducted in this study. Based upon the severity differences in multiple traits exhibited in each of the subgroups of twins, we conducted a series of quantitative trait association analyses to discover SNPs that may associate with the phenotypic differences in trait severity. Quantitative trait association analyses were conducted using the cumulative trait scores for each individual in each twin subgroup and their respective genotyping data derived from the study by Wang et al. (2009). QTNs with unadjusted *p*-values ≤ 1 × 10^−5^ were selected for subsequent case-control association analyses. Results of the QTL analyses for each subgroup of twins are shown in Tables S1–S6 in Supplementary Materials. The combined sets of genes associated with the QTNs across all twin groups are shown in Table 1, with a Venn diagram showing the distribution of the overlapping genes among four of the five traits (Figure 4). Notably, there were no genes shared between those associated with perseverative behaviors and any of the other four traits. Hypergeometric distribution analyses of each set of QTN-associated genes indicated significant enrichment in genes from the SFARI database for all traits except non-verbal communication (Table 2).

### 2.3. Case-Control Association Analyses Based on Individuals Stratified by Trait Severity for Each Subgroup of Twins

The SNPs used for case-control association analyses were limited to the trait-associated SNPs derived from QTL analyses involving the corresponding twin groups. Results of these case-control association analyses are presented in Tables S7–S12. Examination of these results for each set of twins reveals significant SNPs (corrected for multiple testing by Bonferroni and/or Benjamini–Hochberg methods) that differentiate between cases and controls. Moreover, for all SNPs that were found to be significantly associated with both the lowest quartile (Q1) and highest quartile (Q4) cases diagnosed with ASD, the minor allele frequencies (MAF) relative to that of controls were noticeably different, resulting in different odds ratios. That is, a specific trait-associated SNP that may be considered as predisposing to ASD in the Q4 subgroup with a high odds ratio (>1.0) was found to have a lower odds ratio (often <1.0) in the Q1 subgroup, further suggesting relevance to severity of that specific trait. Although a SNP may occasionally have a higher odds ratio in the Q1 than in the Q4 case group, the differences in MAFs still suggest that these subgroups based on trait severity are genetically distinct with respect to that genetic variant. Notably, the majority of significant SNPs in the non-autistic subgroups (dNA.MZ and dNA.DZ) were found in Q4, at the highest severity end of the trait distribution.

### 2.4. Enrichment of Known Autism Risk Genes from Case-Control Association Analyses

The SNP-associated genes derived from the case-control association analyses described above were compared to the complete set of autism risk genes that comprise the SFARI gene database. Table 3 shows that genes mapped to the trait-associated SNPs that are significant by case-control association analyses using individuals in both Q1 and Q4 as cases for each subgroup of twins are enriched among SFARI genes in three of the six twin groups (dNA.MZ, dNA.DZ, and cDZ). Together with the results shown in Table 2, these findings suggest that the experimental strategy of combining quantitative trait association analyses with case-control association analyses using cases stratified by trait severity is capable of identifying significant autism risk genes even with relatively small numbers of samples. Furthermore, the SNPs identified can be associated with severity of specific traits, thereby associating genotype with phenotype.

### 2.5. Functional Analysis of Genes Associated with QTNs from Quantitative Trait and Case-Control Association Analyses

Genes implicated by QTNs were analyzed using Ingenuity Pathway Analysis (IPA) software with a focus on identifying genes that impact nervous system development and function. Table 4, Table 5, Table 6, Table 7 and Table 8 show the neurological functions that are statistically enriched among genes implicated by QTNs associated with spoken language, nonverbal communication, play skills, social skills, and perseverative behaviors, respectively, for each subgroup of twins. Notably, many of the annotated functions are relevant to what is known about specific neuronal processes disrupted in ASD. Moreover, IPA analyses of genes associated with significant SNPs identified by case-control association analyses of both dNA.MZ and dNA.DZ subgroups in addition to the cDZ subgroup which are all enriched in SFARI genes also revealed significant over-representation of genes involved in neurological functions, behaviors, and disorders relevant to ASD, as shown in Table 9, Table 10 and Table 11, respectively.

### 2.6. Case-Control Association Analysis Using dNA.DZ Twins as Controls for dA.DZ Co-Twins

Given the differential diagnosis of discordant dizygotic twins with presumed genetic differences between the autistic and non-autistic co-twins, we conducted an exploratory case-control association analysis using the dNA.DZ twins (*n* = 36) as controls for the diagnosed dA.DZ co-twins (*n* = 38). Interestingly, over 140 genes were associated with a total of 390 SNPs that exhibited a nominal *p*-value ≤ 0.05 and odds ratio ≥ 3 (Table 12, complete case-control association data in Table S13). Hypergeometric distribution analyses showed that this set of genes was highly enriched for autism risk genes from the SFARI database (*q* = 1.84 × 10^−7^) and from an exome-sequencing study [23] (*q* = 4.64 × 10^−8^). Pathway analysis of this gene set using IPA revealed that netrin signaling (involving PRKG1, DCC, and PRKAG2) and axon guidance signaling (involving PRKCE, PDGFD, DCC, ROBO1, SLIT3, PRKAG2, NTNG1, PLCG2) were among the top 10 canonical pathways represented with Fisher’s Exact *p*-values of 2.56 × 10^−3^ and 6.18 × 10^−3^, respectively. Several of the above-mentioned genes (specifically, PRKCE, PRKAG2, and PLCG2) are also significantly over-represented in additional pathways potentially relevant to ASD, including melatonin signaling (*p* = 1.18 × 10^−2^) and synaptic long-term potentiation (*p* = 4.45 × 10^−2^). Functional analysis showed significant over-representation of many neurological processes commonly impacted by autism, notably neuritogenesis, axonogenesis, neuronal migration, and axon guidance (Table 13).

## 3. Discussion

The goals of this study were to: (1) examine the influence of genetics on specific behaviors and traits of ASD; (2) identify SNPs (i.e., QTNs) that associate with a particular ASD trait; (3) determine if trait QTNs can discriminate between cases and controls, where the cases are individuals at the extremes of phenotypic severity across five autistic traits; and (4) to identify pathways and functions implicated by the genes associated with the QTNs. To accomplish these goals, we developed a novel approach which utilized MZ and DZ twins who are either concordant or discordant for diagnosis of autism to reveal differences in phenotypic severity among and within the subgroups of twins defined by diagnostic status and by zygosity. Then, we used the phenotypic (trait) profiles of each subgroup of twins to perform quantitative trait association analyses to identify SNPs that may be functionally associated with the respective ASD trait (i.e., QTNs). Case-control association analyses were then performed with the QTNs for five autistic behavioral traits using the six different subgroups of twins at the extreme ends (i.e., lowest and highest quartiles) of the respective quantitative trait distribution curves as cases and a large group of unrelated controls. We show here that this novel combination of experimental strategies allowed us to identify significant SNPs that could distinguish individuals with autism from controls. Genes harboring these SNPs are significantly enriched for autism risk genes annotated by SFARI Gene.

### 3.1. Severity of Autistic Traits Are Strongly Influenced by Genetics

Numerous studies using both monozygotic and dizygotic twins have sought to determine the role of genetics versus environment with varying results, as described earlier. The quantitative trait distribution profiles generated in this study demonstrate significant differences in severity between clinically discordant non-autistic MZ and DZ co-twins, with the profiles of the MZ twins being significantly more severe than DZ twins across all traits, while the profiles of all diagnosed twins are virtually indistinguishable from each other, regardless of zygosity (Figure 1 and Figure 2). These differences in trait severity between the non-autistic MZ and DZ twins are seen even at the most fundamental level, that is, at the level of specific ADI-R items which comprise each trait, demonstrating the strong influence of genetics on a broad spectrum of ASD-associated behaviors and characteristics (Figure 3). In other words, a non-autistic identical twin exhibits a significantly higher severity of autistic traits in comparison to an undiagnosed non-identical twin who shares only a part of his/her autistic co-twin’s genes.

### 3.2. Quantitative Trait Association Analyses Reveal SNPs that Associate with a Specific ASD Trait

QTL association analyses allowed us to discover SNPs that may be functionally relevant to autism traits. The genes associated with trait QTNs (Table 1) are over-represented in a number of neurological functions related to autism. The neurological functions associated with each set of trait genes are shown in Table 4, Table 5, Table 6, Table 7 and Table 8. SNPs associated with spoken language implicate genes involved in cell–cell adhesion of neurons, axon guidance and fasciculation, GABA-mediated receptor currents, and morphology of different brain regions, including the cerebral cortex, cerebellum, and corpus callosum, which are all impacted in ASD (Table 4). Another set of SNPs associated with nonverbal communication implicate genes that play a role in cell–cell adhesion of astrocytes, organization and morphology of mossy fibers, axon pruning, migration of pyramidal neurons, and synaptic transmission of collateral synapses (Table 5). SNPs associated with play skills highlight genes involved in dendritic spine formation, synaptic transmission, and long-term potentiation of Purkinje cells (Table 6). Purkinje cell morphology, neurotransmission, axonogenesis, and differentiation of motor neurons are functions over-represented among genes associated with social QTNs (Table 7). QTNs associated with perseverative behaviors point to genes involved in epileptic encephalopathy, loss of hippocampal neurons, and certain higher level functions such as social transmission of food preference, mating, spatial learning, and motor learning (Table 8). Interestingly, the genes associated with the QTNs for perseverative behaviors do not overlap with those associated with any of the other traits, while there is an overlap of 20 genes harboring QTNs for nonverbal, play and social skills (Figure 4), suggesting interrelated proficiencies involving the latter functions. These studies thus help to link genotype to genes and phenotypes and reveal some of the biological processes that may underlie specific ASD traits. The relevance of the above-described neurological functions to ASD further suggested that some of these QTNs may be able to distinguish cases from controls.

### 3.3. Trait-Associated QTNs Can Discriminate between Cases and Controls

Case-control association analyses using individuals at the extremes of phenotypic (i.e., trait) severity (Q1 and Q4) as cases enabled the identification of significant trait QTNs that were demonstrated to discriminate between cases and controls. This approach allowed reduction in the phenotypic heterogeneity of the individuals within each subgroup of twins for genetic analyses. In fact, genetic differences between the first and fourth quartiles of trait severity for all twin subgroups are quite clear. Notably, for SNPs associated with both Q1 and Q4 subgroups, the odds ratios for the two extreme groups are distinctly different and often in opposite directions (that is, greater or less than 1) relative to unrelated controls (Tables S7–S12), thereby supporting the associative nature of the specific SNP with that particular trait. Thus, the genetic heterogeneity of cases with respect to these trait-associated SNPs reflects the phenotypic heterogeneity of individuals at the extreme ends of the trait distribution profiles. In addition, hypergeometric distribution analyses of the genes derived from case-control analyses of twin subgroups show significant enrichment in SFARI autism risk genes in three of the twin subgroups (dNA.MZ, dNA.DZ, and cDZ) (Table 3). The over-representation of SFARI-annotated autism risk genes among those revealed here through case-control analyses indicates that the reduction in heterogeneity of cases at the extremes of the trait spectrum allows the detection of significant genetic variants differentiating cases and controls with relatively few samples, while also implicating novel ASD-associated genes that may be replicated by future studies. Although there is precedence for the use of individuals at the extremes of autistic traits for phenotype analyses [24,25,26,27], no other study has utilized individuals within these phenotypically extreme populations for genome-wide genetic association analyses.

### 3.4. Genetic Differences between Discordant DZ Twins Strongly Associate with Autism Risk Genes

The availability of genome-wide genotype data for both autistic and non-autistic DZ co-twins in our study prompted us to examine the feasibility of performing a case-control analysis in which the group of non-autistic DZ twins was used as a control for the group of autistic co-twins. Although none of the 3400 SNPs with raw *p*-values ≤ 0.05 remained significant after correction for multiple testing, there were 390 SNPs with nominal *p*-values ≤ 0.05 and odds ratios ≥ 3 (ranging from 3 to 11) (Table S13). Hypergeometric distribution analysis of the 146 SNP-associated protein-coding genes showed significant enrichment in ASD-risk genes annotated by SFARI Gene as well as in genes revealed by exome sequencing [23]. Table 13 shows that this set of genes is highly enriched for ASD-associated neuronal functions including development, migration and pathfinding of neurons, neuritogenesis, axonogenesis, and guidance of axons, as well as synaptic development and transmission.

While the use of discordant non-autistic DZ twins as a control group for their autistic co-twins in case-control association analyses may seem unconventional, there is precedence for family-based genetic studies in which concordant MZ twin pairs were compared against their parents by whole genome and exome sequencing, with each study revealing novel and rare variants in the probands [28,29]. The use of both concordant and discordant MZ and DZ twins to investigate the contribution of pre-and post-zygotic de novo CNVs to ASD and other neurodevelopmental disorders [30] further illustrates the value of using diagnostic concordance and discordance in dissecting the complex genetic underpinnings of ASD.

### 3.5. Advantages and Limitations of Study Design

An advantage of quantitative trait association analysis is that it allows the identification of SNPs that may potentially be more functionally related to the condition, thus reducing the number of SNPs to be considered for further case-control association analyses. A possible disadvantage is that this procedure may exclude some ASD-relevant but not trait-associated SNPs from case-control analyses. Another limitation is that we did not separately analyze male and female twins due to the relatively small and variable number of females in the different subgroups of twins, and thus cannot eliminate sex as a confounding factor. However, a prior analysis of affected males and females from the AGRE repository using cluster analyses of ADI-R scores as described previously [17] showed no obvious sex differences in subphenotypes [31].

Because of the extreme heterogeneity of ASD, there has been increasing attempts to stratify or subgroup individuals with ASD by specific phenotypes to obtain more homogeneous sample groups for large-scale genetics analyses. While some autism studies have been conducted using individuals at the top 5% of an autistic trait [24,25,27,32], no other study has performed genetic case-control association analyses on subgroups of twins stratified by trait severity in this manner. Although the use of both MZ and DZ twins at the extremes of trait severity reduces the genetic heterogeneity of ‘cases’ when compared against a large group of unrelated controls, sample sizes at these extremes are a limitation of this study. Another limitation is that there is no empirical validation of the significant SNPs identified by case-control analyses as this was purely an in silico study, and we did not have access to the twins’ biological samples for follow-up genotype analyses. On the other hand, the identification of some significant SNPs (with different odds ratios) for samples in the first and fourth quartiles of trait severity in case-control analyses replicates the association of those SNPs with ASD in distinct subgroups of individuals. Despite these limitations, case-control analyses revealed a number of significant trait-associated SNPs that implicate genes that converge on certain neurological functions associated with ASD, suggesting that disruption of those functions or pathways may play a role in the manifestation of that specific trait in ASD. Notably, multiple genes are associated with each of the five traits reflecting the multi-genic nature of ASD, even at these fundamental levels. However, no information is available on the functional significance of the specific SNPs with respect to gene regulation or to protein function and expression, and many SNPs are in noncoding regions which require further exploration.

Case-control association analyses using clinically discordant DZ twins as both cases and controls, while underpowered, identified a large number of nominally significant SNPs associated with genes that are significantly enriched in autism risk genes. While this study shows that reduction of genetic heterogeneity by stratification of cases according to trait severity or by use of discordant DZ twins as cases and controls increases the ability to identify common genetic variants associated with ASD, future studies with greater numbers of individuals in each subgroup are required to replicate these findings.

## 4. Summary

We present here a novel experimental design using MZ and DZ twins, either concordant or discordant for ASD, which evaluates the role of genetics in contributing to the severity of autistic traits and identifies significant SNPs that can distinguish cases from unaffected controls by capitalizing on the extreme phenotypic differences exhibited especially by discordant MZ and DZ twins. Although prior studies have consistently indicated that genetics is a major contributor to the overall diagnosis of ASD, deep phenotypic profiling shows that almost all ASD traits are significantly more severe in non-autistic MZ twins than in non-autistic DZ twins, suggesting a dominant influence of genotype on ASD phenotypes probed by the majority of items on the ADI-R diagnostic instrument [16]. At the same time, the similarity of trait-severity profiles among all autistic subgroups suggests that the ADI-R, often considered the gold-standard instrument for autism diagnostics, captures the essential components of ASD across the ASD population.

Quantitative trait association analyses using ASD trait profiles reveal SNPs (QTNs) associated with genes that provide meaningful insight into neurological functions and pathways commonly associated with ASD, thus linking genotype to phenotype (i.e., specific traits) as well as to underlying molecular pathology. Moreover, reducing phenotypic heterogeneity by using individuals at the extremes of trait severity for case-control association analyses with the trait-associated QTNs resulted in the identification of SNPs that significantly discriminate both affected and non-autistic co-twins from a large group of unrelated controls. These results suggest an underlying genetic liability towards autistic traits, even in the undiagnosed individuals (i.e., non-autistic co-twins). Genes implicated by the QTNs that survive correction for multiple testing in case-control association analyses are statistically enriched in ASD-associated genes revealed by significant overlap with those in the SFARI Gene database. Finally, case-control association analysis using dA.DZ twins as cases and their discordant non-autistic co-twins (dNA.DZ) as ‘controls’ also reveals a large number of neurologically relevant genes that are significantly over-represented in both SFARI Gene and exome sequencing datasets.

## 5. Materials and Methods

### 5.1. ASD Subphenotype Analysis and Generation of Trait Severity Distribution Profiles

Detailed demographic information of the 284 pairs of twins included in this study is shown in Table S14. Of these sets of twins, 88 monozygotic (MZ) pairs were concordant for diagnosis of ASD, while 25 MZ pairs were discordant. Among the dizygotic (DZ) twins, 56 pairs were concordantly autistic, while 115 pairs were discordant for diagnosis. As mentioned earlier, we label the concordant autistic MZ and DZ twin subgroups as cMZ and cDZ, respectively, and the discordant autistic (dA) and non-autistic (dNA) MZ and DZ twin subgroups as dA.MZ, dA.DZ, dNA.MZ, and dNA.DZ, respectively. ADI-R diagnostic scoresheets for all sets of twins were downloaded from the AGRE repository. It should be noted that ADI-R scoresheets were available for both the autistic and non-autistic (discordant) co-twins in order to establish concordance. Raw scores for 88 items from the ADI-R scoresheet which related to five ASD traits (spoken language, nonverbal communication, play skills, social skills, and perservative behaviors) were extracted from each individual’s scoresheet, and the ADI-R scores were then adjusted according to previously described methods [17]. The specific items related to each trait are shown in Table S15. The average score for each ADI-R item, as well as the cumulative score for a specific trait, was calculated for each of the six twin subgroups. Quantitative trait distribution profiles for each twin subgroup were created by graphing each individual’s cumulative score for each trait. To account for differences in the number of individuals in the different subgroups of twins, the total number of individuals in each subgroup was normalized to 100 to facilitate graphical comparisons between trait severity distribution profiles based on zygosity as well as on diagnostic concordance or discordance. The student’s two-sample *t*-test in the StatPac statistical software package (https://statpac.com/index.htm) was used to determine significance of differences in severity scores of overall ASD traits and specific ADI-R items based on *p*-values ≤ 0.05.

### 5.2. Source of Genetic Data

Genome-wide genotype data for the twins and 2438 unaffected (non-autistic) controls were derived from a previously published genome-wide association study (GWAS) by Wang et al. [33], with data previously cleaned by Jennifer K. Lowe in the laboratory of Daniel H. Geschwind, M.D., Ph.D. at UCLA. Briefly, genotyping data were generated on Illumina HumanHap550 BeadChip arrays with over 550,000 SNP markers. Quality control procedures required DNA samples to have ≥ 0.95 call rate with minor allele frequency ≥ 0.05, and *p*-values testing significant deviation from Hardy–Weinberg equilibrium that were ≥ 0.001. Only subjects of European ancestry were genotyped and thus, only Caucasian twins could be included in the genetic association analyses, eliminating concerns about population stratification as a confounding factor. This publicly available dataset contained the genotypes for 74.3% of MZ twins (85 pairs) and ~85.4% of DZ twins (146 pairs) in this study. Controls were comprised of 2438 unrelated non-autistic individuals of European ancestry for whom genome-wide genotyping data was also available from the same GWAS study [33]. The workflow for the genetic association analyses performed on the twin data is shown in Scheme 1 for clarity.

### 5.3. Quantitative Trait Loci (QTL) Association Analyses

PLINK 1.07 software [34] was used to perform all genetic association analyses. Quantitative trait association analyses utilized the cumulative trait scores and the respective genotype data from the above-mentioned dataset for each individual within a specific twin subgroup as previously described [19]. Single nucleotide polymorphisms (SNPs) with unadjusted *p*-value ≤ 1.0 × 10^−5^ were identified as QTNs which were subsequently used in case-control association analyses as described below. The overlap of trait-associated genes implicated by the SNPs was determined using Venny 2.1.0, an online software package for creating Venn diagrams [35].

### 5.4. Case-Control Association Analyses

To reduce ASD heterogeneity for genetic analyses, only cases at the extremes of a defined ASD trait were used in separate case-control association analyses. These individuals exhibited cumulative trait severity scores within the first or fourth quartiles. The first quartile (Q1) was comprised of twins who exhibited the least severe phenotype for that trait and the fourth quartile (Q4) was comprised of twins who exhibited the highest severity for that trait. Cumulative trait scores at Q1 and Q4 for each subgroup of twins were determined using Tyers Box Plot software [36] (see Scheme 1 and Figure 2). Individuals with cumulative scores less than or equal to that determined by Q1 and greater than or equal to that determined by Q4 were used in case-control association analyses against 2438 unrelated controls from the study of Wang et al. [33], focusing on the SNPs discovered by QTL analysis. Discordant non-autistic twins for whom genome-wide genotyping data were available were also stratified and analyzed in the same way. Because only one individual of a pair of MZ twins was included in the original genome-wide genotyping analysis [33], the genotype data of each dA.MZ twin was used for his or her respective dNA.MZ co-twin in case-control analyses. SNPs were considered significant if the adjusted Benjamini–Hochberg FDR was ≤ 0.10.

### 5.5. SNP Annotation and Functional Analyses of Associated Genes

Significant SNPs were annotated using SNPper [37] and/or SNPnexus [38]. Both are web-based applications that allow downloading of information on SNPs according to user-defined criteria, such as chromosomal position, band, alleles, closest gene, role (e.g., coding exon, promoter, intron, 3′-UTR), amino acid change, and position. The SNP-associated genes were analyzed using Ingenuity Pathway Analysis (IPA) software (QIAGEN, Redwood City, CA) with a focus on genes involved in neurological functions and pathways. Significance for enrichment of genes associated with a particular pathway or function included in IPA’s Knowledgebase was determined by right-tailed Fisher’s exact test (*p*-value ≤ 0.05).

### 5.6. Hypergeometric Distribution Analyses for Gene Enrichment

SNP-associated genes were then compared to a list of known autism risk genes in the SFARI Gene database [39] and/or a list of ASD-associated genes identified by an exome sequencing study [23]. Hypergeometric distribution probabilities for over-representation of overlapped genes within the SFARI dataset or among ASD genes identified by exome sequencing were calculated using the CASIO Keisan Online Calculator (http://keisan.casio.com/exec/system/1180573201), with significance determined by an upper cumulative *q*-value ≤ 0.05.

## 6. Conclusions

Deep phenotyping analysis of monozygotic and dizygotic twins concordant or discordant for diagnosis of ASD indicates that the identical genotype of non-autistic MZ twins with their respective affected co-twins strongly influences the severity of autistic traits in comparison to the trait severity expressed by non-autistic DZ twins. Quantitative trait association analyses using trait distribution profiles and genotype data for the respective twin groups identify SNPs linked to traits. Case-control association analyses using these QTNs and individuals at the extremes of trait distribution profiles reveal significant SNPs associated with genes that are functionally significant to the known pathobiology of ASD. Finally, we suggest that more effective treatment strategies may be derived by associating specific phenotypes with genotype-implicated genes and related functional deficits manifested by a given subgroup of individuals.

## Figures and Tables

**Table 1:** Summary of genes associated with QTNs for each trait across all twin groups.

Spoken Language		Nonverbal Commun.		Play Skills		Social Skills				Pers. Behav.
ADAM12	KLHL29	ABHD6	MGAT4C	ADAMTS8	KALRN	ABCC4	DCAF16	LHPP	RCOR1	APOLD1
ALOX12P1	L3MBTL4	ADAMTS8	MICA	ADPRHL1	KIRREL3	ABCD3	DCDC5	LMO1	REV1	BNIP3L
ANGPT1	LHFPL3	AGMO	MTCP1	AGMO	L3MBTL4	ACBD5	DGKH	LPPR4	RNASE8	C2orf88
BMP5	LHX4	AK097143	NCALD	AMPD3	LARGE	ADAMTS8	DLG2	LPPR5	RNF144B	CDH13
BMPR2	LRRFIP1	ANKRD44	NCAPG	ANKRD44	LCORL	ADARB1	DMRT2	LRIG3	RPTOR	CORO2A
C12orf42	LYPD6B	ANKS1B	NOP58	BEND4	LECT1	AGMO	DNAH9	LRRC4C	RUNX1	FAM136B
C3orf55	MGAT4C	BCAS3	NUP210P1	BMPR2	MARK3	AKAP6	DPF3	MAGI3	SGCZ	FHAD1
CCDC152	MGAT5	BMPR2	PAX3	C1orf143	MGAT4C	ALPK1	ELK3	MARK3	SHISA6	GABRR2
CCDC85A	NCALD	CADM1	PCDHGA1	CA10	MTCP1	AMPD2	ELOVL6	MCPH1	SLC16A9	GPC6
CCND3	NOP58	CCDC85A	PIEZO2	CCDC102B	MYH11	ANKRD32	EPHB1	MECOM	SLC44A1	GRAMD1C
CD101	NOS1AP	CCND3	PLCXD3	CCDC60	NCALD	ANKRD44	EPS15	MGAT4C	SLCO2B1	GRIP1
CDK5R1	NPAS3	CDH20	RARB	CCDC8	NCAPG	AOC1	EYS	MMP9	SPATA13	IL31RA
CHRM1	NRXN1	CEP95	RNF24	CCDC85A	NOP58	ARHGAP26	FAM210B	MTCP1	SPOP	KCNAB1
CHRNA7	NTM	CHL1	RYR2	CCND3	NRG2	ARL15	FAM89A	MYH11	SRD5A1	MAML2
CNTN1	OVCH2	CMKLR1	SKAP2	CHN2	PAG1	ASAP1	FAT4	MYO18B	SRGAP3	MRPS6
CNTN4	PAX3	COL20A1	SLC22A23	CHRM3	PARK2	ASXL3	FBXL7	NAALAD2	ST6GALNAC3	MTBP
CPM	PDE4B	CSMD1	SLC27A6	CLMP	PAX3	BC093087	FCER2	NCALD	STAC	MXRA5
CREM	PIEZO2	DCAF16	SPATA13	CMKLR1	PAX7	BMPR2	FHIT	NCAPG	STK11	OR2H2
CSMD1	PPIE	DLG2	STAC	CMTM7	PCDHGA1	BPIFB1	FRRS1L	NDRG2	STX8	PLCB1
DCTN5	SLC1A7	EPHB1	STK32B	COBLL1	PLCXD3	C10orf11	GCNT1	NOP58	SUPT3H	PLD6
DGKH	SLC30A8	EYS	THEG5	CPE	PSD3	C6orf106	GNE	OPA3	THEG5	PRICKLE1
DSCAM	SMEK1	FAT4	TMEM38A	CSMD1	RARB	C9orf171	GRID1	P4HA2	THSD4	PTPRD
DSCAML1	STX8	FLVCR1	TRIOBP	CTNND2	RPTOR	CA10	GRID2	PARK2	TMEM108	RAPGEF5
E2F7	SYT6	GCNT1	WBSCR17	DCAF16	SH3PXD2B	CASP6	GRM7	PAX3	TMEM135	RNLS
EP400	TCHP	IL1R2	WNK2	DFFA	SLC16A9	CASP8	HBS1L	PCDHGA1	TMEM245	SLC22A18
FAM49B	TLN2	ITIH5	ZNF207	DISC1	SLCO4A1	CCDC102B	HDAC11	PCSK5	TNS3	SORBS2
FMN1	TMEM192	KCNIP4		DLG2	SOX5	CCDC85A	HIST1H1D	PDE8A	TRDN	ST8SIA5
GOSR1	TNS3	L3MBTL4		DNAJC10	SPOP	CCND3	HIST1H2BF	PDGFC	TTC27	SYNPO2L
GPR139	TOP2B	LARGE		DPF3	STAC	CCSER1	HIST1H4E	PFKP	USP37	UNC13C
ID3	UNC5D	LCORL		E2F7	STEAP1B	CDH8	HIST1H4G	PIEZO2	WDSUB1	
JAZF1	WWC2	LDB2		EPHB1	STK38	CDK17	IGSF21	PITPNA	WNK2	
KIAA0947	YY1	LIM2		EYS	TECRL	CDK2	IKBKAP	PKD1L1	XYLT1	
KIAA1549		LRRC4C		FAT4	THEG5	CEP95	ITGB3	PLCXD3	ZFP64	
				FIP1L1	TMEM144	CMIP	KCND2	PLXDC1	ZNF142	
				FZD4	TMEM161B	CMKLR1	KCNIP4	PREP	ZNF630	
				GALNTL6	TNFSF12	CMYA5	KIAA0319	PRKCDBP	ZNF804B	
				GCNT1	TP53	CNTN1	KIAA1377	PSD3		
				GPR56	TRDN	COLEC12	KIF13A	PTPRE		
				GREM1	VAV3	CR2	KLF12	PTPRK		
				HBS1L	WNK2	CSMD1	L3MBTL4	RABGAP1L		
				ID3	ZCCHC14	CTNNAL1	LARGE	RARB		
				ITSN1	ZNF385D	CTTNBP2	LCORL	RBFOX1		

**Table 2:** Hypergeometric distribution analyses of trait-associated genes and SFARI genes.

Hypergeometric Distribution Analyses of Enrichment of SFARI Genes Among ASD Trait-Associated Genes			
Trait	Number of Trait-Associated Genes	# Genes Represented within SFARI Gene	*p*-Value * for Enrichment
Language deficits	65	11	4.20 × 10^−4^
Nonverbal communication	59	5	2.01 × 10^−1^
Play skills	84	9	3.30 × 10^−2^
Social skills	162	19	9.48 × 10^−4^
Perseverative behaviors	29	5	1.68 × 10^−2^

* For the hypergeometric distribution analyses, the total number of genes in the population was estimated to be 20,406, and the total number of ASD-associated genes in the SFARI gene database was 1079.

**Table 3:** Hypergeometric analyses of case-control associated genes and SFARI genes.

Case Groups (Combined Q1 and Q4) vs. Unaffected Controls	Number of Case-Associated Genes	# Genes Represented within SFARI Gene	Q-Value * for Enrichment
dA.MZ	13	0	1
dA.DZ	8	0	1
dNA.MZ	53	9	1.69 × 10^−3^
dNA.DZ	19	4	1.59 × 10^−2^
cMZ	29	2	4.58 × 10^−1^
cDZ	23	4	3.10 × 10^−2^

* For the hypergeometric distribution analyses, the total number of genes in the population was estimated to be 20,406; and the total number of ASD-associated genes in the SFARI gene database was 1079.

**Table 4:** Neurological functions associated with genes implicated by language QTNs.

Functions Annotation	*p*-Value *	Genes
cell-cell adhesion of neurons	9.52 × 10^−6^	*CDK5R1, CNTN4, NRXN1*
guidance of axons	9.66 × 10^−6^	*CDK5R1, CNTN1, CNTN4, LHX4, NRXN1, UNC5D*
fasciculation of axons	8.24 × 10^−5^	*CDK5R1, CNTN1, CNTN4*
abnormal morphology of brain	1.86 × 10^−4^	*CDK5R1, CHRM1, CHRNA7, CNTN1, ID3, LHX4, NPAS3, TOP2B*
GABA-mediated receptor currents	6.98 × 10^−4^	*CHRNA7, NRXN1*
abnormal morphology of granule cells	9.17 × 10^−4^	*CDK5R1, CNTN1*
abnormal morphology of nervous system	1.48 × 10^−3^	*CDK5R1, CHRM1, CHRNA7, CNTN1, CNTN4, ID3, LHX4, NPAS3, TOP2B*
stratification of cerebral cortex	1.75 × 10^−3^	*CDK5R1, TOP2B*
formation of brain	2.52 × 10^−3^	*BMP5, CDK5R1, CNTN1, CREM, DSCAML1, NPAS3, TOP2B*
memory	2.58 × 10^−3^	*CDK5R1, CHRM1, CHRNA7, CREM, NPAS3*
abnormal morphology of molecular layer of cerebellum	2.63 × 10^−3^	*CDK5R1, CNTN1*
formation of enteric ganglion	2.82 × 10^−3^	*PAX3*
morphology of motor cortex	2.82 × 10^−3^	*CDK5R1*
origination of axons	2.82 × 10^−3^	*CDK5R1*
reorganization of molecular layer	2.82 × 10^−3^	*CDK5R1*
sprouting of mossy fiber cells	2.82 × 10^−3^	*CDK5R1*

* Fisher exact *p*-value which indicates the probability that the list of SNP-associated genes is not enriched for annotated function, using genes in IPA’s Knowledgebase as the reference set of genes.

**Table 5:** Neurological functions associated with genes implicated by nonverbal QTNs.

Functions Annotation	*p*-Value *	Genes
organization of mossy fibers	2.60 × 10^−^^3^	*CHL1*
formation of enteric ganglion	2.60 × 10^−^^3^	*PAX3*
targeting of retinal ganglion cells	2.60 × 10^−^^3^	*EPHB1*
cell-cell adhesion of astrocytes	2.60 × 10^−^^3^	*CADM1*
maintenance of neural crest	5.18 × 10^−^^3^	*PAX3*
survival of neural crest	5.18 × 10^−^^3^	*PAX3*
innervation of forelimb	7.77 × 10^−^^3^	*BMPR2*
differentiation of satellite cells	1.03 × 10^−^^2^	*PAX3*
morphogenesis of optic nerve	1.29 × 10^−^^2^	*EPHB1*
migration of pyramidal neurons	1.29 × 10^−^^2^	*CHL1*
abnormal pruning of axons	1.29 × 10^−^^2^	*EPHB1*
excitatory postsynaptic potential of neurons	1.39 × 10^−^^2^	*DLG2, RARB*
formation of pyramidal neurons	1.55 × 10^−^^2^	*CHL1*
synaptic depression of collateral synapses	1.80 × 10^−^^2^	*EPHB1*
synaptic transmission of collateral synapses	1.80 × 10^−^^2^	*EPHB1*
abnormal morphology of axons	2.29 × 10^−^^2^	*CHL1, EPHB1*
development of retinal pigment epithelium	2.31 × 10^−^^2^	*RARB*
abnormal morphology of optic tract	2.31 × 10^−^^2^	*EPHB1*
abnormal morphology of hypoglossal nerve	2.82 × 10^−^^2^	*RARB*
abnormal morphology of olfactory receptor neurons	3.32 × 10^−^^2^	*CHL1*
abnormal morphology of mossy fibers	4.07 × 10^−^^2^	*CHL1*
projection of axons	4.57 × 10^−^^2^	*CHL1*
synaptic transmission of nervous tissue	4.74 × 10^−^^2^	*ANKS1B, EPHB1*
abnormal morphology of neural arch	4.82 × 10^−^^2^	*RARB*

* Fisher exact *p*-value which indicates the probability that the list of SNP-associated genes is not enriched for annotated function, using genes in IPA’s Knowledgebase as the reference set of genes.

**Table 6:** Neurological functions associated with genes implicated by play skills QTNs.

Functions Annotation	*p*-Value *	Genes
formation of dendritic spines	6.97 × 10^−^^4^	*ITSN1, KALRN*
short-term depression of calyx-type synapse	7.29 × 10^−^^4^	*ITSN1*
neurotransmission of synapse	8.88 × 10^−^^4^	*CHRM3, PRKN*
formation of tip of neurite-like extensions	1.46 × 10^−^^3^	*KALRN*
elongation of dendritic spine neck	1.46 × 10^−^^3^	*ITSN1*
long-term potentiation of Purkinje cells	1.46 × 10^−^^3^	*CHRM3*
arrest in axonal transport of mitochondria	1.46 × 10^−^^3^	*PRKN*
quantity of catecholaminergic neurons	1.46 × 10^−^^3^	*PRKN*
length of neurons	2.18 × 10^−^^3^	*ITSN1, KALRN*
excitation of striatal neurons	2.18 × 10^−^^3^	*PRKN*
differentiation of satellite cells	2.91 × 10^−^^3^	*PAX7*
replenishment of synaptic vesicles	2.91 × 10^−^^3^	*ITSN1*
abnormal morphology of locus ceruleus	2.91 × 10^−^^3^	*PRKN*
afterhyperpolarization of pyramidal neurons	4.36 × 10^−^^3^	*CHRM3*
morphology of corticcal neurons	4.36 × 10^−^^3^	*KALRN*
sensorimotor integration	5.09 × 10^−^^3^	*PRKN*
abnormal morphology of substantia nigra	5.81 × 10^−^^3^	*PRKN*
neurotransmission	6.89 × 10^−^^3^	*CHRM3,,KALRN, PRKN*
morphogenesis of dendritic spines	7.26 × 10^−^^3^	*KALRN*
retraction of dendrites	7.99 × 10^−^^3^	*KALRN*
maturation of dendritic spines	8.71 × 10^−^^3^	*KALRN*
length of axons	9.43 × 10^−^^3^	*KALRN*
abnormal morphology of cerebral cortex	9.91 × 10^−^^3^	*KALRN,,PRKN*
length of dendritic spines	1.38 × 10^−2^	*ITSN1*
coordination	1.43 × 10^−2^	*KALRN, PRKN*

* Fisher exact *p*-value which indicates the probability that the list of SNP-associated genes is not enriched for annotated function, using genes in IPA’s Knowledgebase as the reference set of genes.

**Table 7:** Neurological functions associated with genes implicated by social skills QTNs.

Functions Annotation	*p*-Value *	Genes
abnormal morphology of thoracic vertebra	1.19 × 10^−^^3^	*DMRT2, FAT4, NDRG2*
action potential of neurons	4.63 × 10^−^^3^	*DLG2, GRID2, KCND2, PARK7, RARB*
abnormal morphology of Purkinje cells	4.78 × 10^−^^3^	*CNTN1, GRID2, PITPNA*
innervation of climbing fiber	7.24 × 10^−^^3^	*GRID2*
formation of enteric ganglion	7.24 × 10^−^^3^	*PAX3*
development of climbing fiber	7.24 × 10^−^^3^	*GRID2*
differentiation of branchial motor neurons	7.24 × 10^−^^3^	*RUNX1*
targeting of retinal ganglion cells	7.24 × 10^−^^3^	*EPHB1*
differentiation of visceral motor neurons	7.24 × 10^−^^3^	*RUNX1*
abnormal morphology of Golgi interneurons	7.24 × 10^−^^3^	*CNTN1*
proliferation of neuroglia	7.26 × 10^−^^3^	*CCND3, CDK2, CNTN1, MMP9, RUNX1*
abnormal morphology of white matter	7.39 × 10^−^^3^	*PITPNA, SRGAP3*
function of blood-brain barrier	9.99 × 10^−^^3^	*ABCC4, MMP9*
abnormal morphology of lumbar vertebra	1.09 × 10^−^^2^	*DMRT2, FAT4*
function of brain	1.12 × 10^−^^2^	*ABCC4, KCNIP4, MMP9*
morphology of brain	1.18 × 10^−^^2^	*CASP6, CNTN1, EPHB1, GRID1, GRID2, MCPH1, MECOM, PARK7, PITPNA, SRGAP3*
axonogenesis	1.31 × 10^−^^2^	*CASP6, EPHB1, GRID2, LRRC4C, PTPRE, STK11*
arrest in cell cycle progression of oligodendrocyte precursor cells	1.44 × 10^−^^2^	*CDK2*
abnormal morphology of superior ganglion of glossopharyngeal nerve	1.44 × 10^−^^2^	*MECOM*
delay in initiation of pruning of axons	1.44 × 10^−^^2^	*CASP6*
maintenance of neural crest	1.44 × 10^−^^2^	*PAX3*
blood-cerebrospinal fluid barrier function	1.44 × 10^−^^2^	*ABCC4*

* Fisher exact *p*-value which indicates the probability that the list of SNP-associated genes is not enriched for annotated function, using genes in IPA’s Knowledgebase as the reference set of genes.

**Table 8:** Neurological functions associated with genes implicated by Perseverative behavior QTNs.

Diseases or Functions Annotation	*p*-value *	Genes
paired-pulse facilitation of parallel fiber-Purkinje cell synapses	1.27 × 10^−^^3^	*UNC13C*
early infantile epileptic encephalopathy type 12	1.27 × 10^−^^3^	*PLCB1*
loss of hippocampal neurons	6.36 × 10^−^^3^	*PLCB1*
epilepsy	1.27 × 10^−2^	*GABRR2, PLCB1, PRICKLE1*
afterhyperpolarization of neurons	2.27 × 10^−2^	*KCNAB1*
progressive myoclonic epilepsy	2.64 × 10^−2^	*PRICKLE1*
status epilepticus	4.13 × 10^−2^	*GABRR2*
density of synapse	4.61 × 10^−2^	*GRIP1*
sedation	4.61 × 10^−2^	*GABRR2*

* Fisher exact *p*-value which indicates the probability that the list of SNP-associated genes is not enriched for annotated function, using genes in IPA’s Knowledgebase as the reference set of genes.

**Table 9:** Neurological functions implicated by QTN-associated genes in dNA.MZ.

Functions Annotation	*p*-value *	Genes
abnormal morphology of pyramidal neurons	5.59 × 10^−^^5^	*CHL1, CHRNA7, KALRN*
GABA-mediated receptor currents	4.19 × 10^−^^4^	*CHRNA7, NRXN1*
abnormal morphology of cerebral cortex	1.12 × 10^−^^3^	*CHL1, CHRNA7, KALRN, NPAS3*
organization of mossy fibers	2.19 × 10^−^^3^	*CHL1*
cell-cell adhesion of astrocytes	2.19 × 10^−^^3^	*CADM1*
developmental process of synapse	2.35 × 10^−^^3^	*CADM1, CHRNA7, KALRN, NRXN1*
formation of tip of neurite-like extensions	4.37 × 10^−3^	*KALRN*
hyperexcitation of hippocampal neurons; short-term potentiation of hippocampus; nicotine-mediated receptor current	4.37 × 10^−^^3^	*CHRNA7*
abnormal morphology of enlarged fourth cerebral ventricle	6.54 × 10^−^^3^	*NPAS3*
miniature excitatory postsynaptic currents	7.15 × 10^−^^3^	*KALRN, NRXN1*
abnormal morphology of lateral cerebral ventricle	7.64 × 10^−^^3^	*CHL1, NPAS3*
differentiation of satellite cells	8.71 × 10^−^^3^	*PAX7*
abnormal morphology of cingulate gyrus	8.71 × 10^−^^3^	*NPAS3*
abnormal morphology of GABAergic neurons	8.71 × 10^−^^3^	*CHRNA7*
migration of pyramidal neurons	1.09 × 10^−^^2^	*CHL1*
pervasive developmental disorder	1.13 × 10^−2^	*BCAS3, PREP, SLC1A7*
excitatory postsynaptic current	1.26 × 10^−^^2^	*KALRN, NRXN1*
morphology of cortical neurons	1.30 × 10^−2^	*KALRN*
formation of pyramidal neurons	1.30 × 10^−2^	*CHL1*
maturatin of neurons	1.46 × 10^−2^	*KALRN, NRXN1*
branching of axons	1.49 × 10^−^^2^	*KALRN, STK11*
prepulse inhibition	1.56 × 10^−^^2^	*KALRN, NRXN1*
abnormal morphology of enlarged third cerebral ventricle	1.74 × 10^−^^2^	*NPAS3*
paired-pulse inhibition	1.74 × 10^−^^2^	*NRXN1*
coordination	1.79 × 10^−^^2^	*CHRNA7, KALRN, NPAS3*
abnormal morphology of cerebral aqueduct	1.95 × 10^−^^2^	*NPAS3*
morphogenesis of dendritic spines	2.16 × 10^−^^2^	*KALRN*
retraction of dendrites	2.38 × 10^−2^	*KALRN*
activation of dopaminergic neurons	2.59 × 10^−^^2^	*CHRNA7*
maturation of dendritic spines	2.59 × 10^−^^2^	*KALRN*
abnormal morphology of olfactory receptor neurons	2.81 × 10^−^^2^	*CHL1*
length of axons	2.81 × 10^−^^2^	*KALRN*
spatial memory	2.88 × 10^−^^2^	*CHRNA7, KALRN, NPAS3*
sleep disorders	3.06 × 10^−^^2^	*CHRNA7, PDE4B*
neurotransmission	3.20 × 10^−^^2^	*ANKS1B, CHRNA7, KALRN, NRXN1*
development of neurons	3.44 × 10^−^^2^	*CADM1, CHL1, CHRNA7, KALRN, NRXN1, STK11*

* Fisher exact *p*-value which indicates the probability that the list of SNP-associated genes is not enriched for annotated function, using genes in IPA’s Knowledgebase as the reference set of genes.

**Table 10:** Neurological functions implicated by QTN-associated genes in dNA.DZ.

Diseases or Functions Annotation	*p*-Value *	Genes
adhesion of cerebellar granule cell	8.65 × 10^−^^4^	*ADGRG1*
migration of CA1 neuron	8.65 × 10^−^^4^	*DISC1*
abnormal morphology of lumbar dorsal root ganglion	8.62 × 10^−3^	*GABBR1*
abnormal morphology of somatic nervous system	1.03 × 10^−2^	*GABBR1*
pervasive developmental disorder	1.44 × 10^−2^	*CORO2A, GABBR1*
abnormal morphology of sciatic nerve	1.46 × 10^−2^	*GABBR1*
positioning of neurons	1.55 × 10^−2^	*DISC1*
development of olfactory cilia	1.80 × 10^−2^	*DISC1*
progressive myoclonic epilepsy	1.80 × 10^−2^	*PRICKLE1*
maturation of synapse	1.89 × 10^−2^	*DISC1*
migration of neuronal progenitor cells	1.97 × 10^−2^	*ADGGRG1*
fragile X syndrome	2.48 × 10^−2^	*GABBR1*
elongation of axons	2.73 × 10^−2^	*DISC1*
migration of neurons	2.81 × 10^−2^	*ADGRG1, DISC1*
abnormal morphology of myelin sheath	2.99 × 10^−2^	*GABBR1*
differentiation of oligodendroccyte precursor cells	3.24 × 10^−2^	*DISC1*
absense seizure	3.24 × 10^−2^	*GABBR1*
passive avoidance learning	3.49 × 10^−2^	*GABBR1*
inhibitory postsynaptic current	4.41 × 10^−2^	*GABBR1*
electrophysiology of nervous system	4.74 × 10^−2^	*GABBR1*

* Fisher exact *p*-value which indicates the probability that the list of SNP-associated genes is not enriched for annotated function, using genes in IPA’s Knowledgebase as the reference set of genes.

**Table 11:** Neurological functions implicated by QTN-associated genes in cDZ.

Diseases or Functions Annotation	*p*-Value *	Genes
*morphology of brain*	1.51 × 10^−3^	*DSCAM, LHX4, PRKN, TOP2B*
*abnormal morphology of abducens nerve*	1.73 × 10^−3^	*LHX4*
*arrest in axonal transport of mitochondria*	1.73 × 10^−3^	*PRKN*
*quantity of catecholaminergic neurons*	1.73 × 10^−3^	*PRKN*
*delay in specification of corticotroph cells*	1.73 × 10^−3^	*LHX4*
*excitation of striatal neurons*	2.59 × 10^−3^	*PRKN*
*startle response*	2.60 × 10^−3^	*PLCB1, PRKN*
*development of granule cell layer*	3.46 × 10^−3^	*CREM*
*function of central pattern generator*	3.46 × 10^−3^	*DSCAM, LHX4, PRKN, TOP2B*
*development of cortical subplate*	3.46 × 10^−3^	*TOP2B*
*abnormal morphology of locus ceruleus*	3.46 × 10^−3^	*PRKN*
*morphogenesis of dendrites*	3.67 × 10^−3^	*DSCAM, FMN1*
*loss of hippocampal neurons*	4.32 × 10^−3^	*PLCB1, PRKN*
*development of central nervous system*	5.75 × 10^−3^	*CREM, PLCB1, PRKN, TOP2B*
*abnormal morphology of amacrine cells*	6.04 × 10^−3^	*DSCAM*
*sensorimotor integration*	6.04 × 10^−3^	*PRKN*
*abnormal morphology of substantia nigra*	6.90 × 10^−3^	*PRKN*
*abnormal morphology of neurons*	7.53 × 10^−3^	*DSCAM, LHX4, PRKN*
*abnormal morphology of nervous system*	8.50 × 10^−3^	*DSCAM, LHX4, PRKN, TOP2B*
*abnormal morphology of hypoglossal nerve*	9.48 × 10^−3^	*LHX4*
*thickness of cerebral cortex*	1.03 × 10^−2^	*DSCAM*
*quantity of gonadotropes*	1.12 × 10^−2^	*LHX4*
*innervation of motor neurons*	1.20 × 10^−2^	*TOP2B*
*abnormal morphology of brain*	1.22 × 10^−2^	*LHX4, PRKN, TOP2B*
*loss of neurons*	1.27 × 10^−2^	*PLCB1, PRKN*
*curvature of vertebral column*	1.80 × 10^−2^	*TOP2B*
*formation of brain*	1.83 × 10^−2^	*CREM, PLCB1, TOP2B*
*stratification of cerebral cortex*	1.89 × 10^−2^	*TOP2B*
*innervation of muscle*	1.89 × 10^−2^	*TOP2B*
*abnormal morphology of adenohypophysis*	1.97 × 10^−2^	*LHX4*
*quantity of nerve ending*	2.14 × 10^−2^	*PRKN*
*guidance of motor axons*	2.23 × 10^−2^	*LHX4*
*loss of dopaminergic neurons*	2.23 × 10^−2^	*PRKN*
*abnormal morphology of spinal nerve*	2.48 × 10^−2^	*LHX4*
*fasciculation of axons*	2.56 × 10^−2^	*DSCAM*
*abnormal morphology of motor neurons*	2.82 × 10^−2^	*LHX4*
*neuritogenesis*	3.00 × 10^−2^	*DSCAM, FMN1, TOP2B*
*abnormal morphology of subventricular zone*	3.07 × 10^−2^	*TOP2B*
*memory*	3.36 × 10^−2^	*CREM, PLCB1*
*morphology of dendritic spines*	3.57 × 10^−2^	*DSCAM*
*quantity of dendritic spines*	3.99 × 10^−2^	*DSCAM*
*development of spinal cord*	4.49 × 10^−2^	*TOP2B*
*abnormal morphology of olfactory bulb*	4.57 × 10^−2^	*TOP2B*
*cell viability of cortical neurons*	4.74 × 10^−2^	*PRKN*
*electrophysiology of nervous system*	4.74 × 10^−2^	*PRKN*

* Fisher exact *p*-value which indicates the probability that the list of SNP-associated genes is not enriched for annotated function, using genes in IPA’s Knowledgebase as the reference set of genes.

**Table 12:** Genes associated with SNPs identified by case-control association analyses using dA.DZ twins as cases and dNA.DZ twins as controls.

Case-control Association Analysis of dA.DZ vs. dNA.DZ					Overlap with SFARI	Overlap with Exome-seq ^#^	
(146 genes associated with SNPs with *p* ≤ 0.05 and OR ≥ 3)					* Q = 1.84 × 10^−7^	* Q = 4.64 × 10^−8^	
ABCA13	DCC	KCTD16	PCSK6	RPH3AL	ABCA13	ABCA13	OR1J2
ACVRL1	DKK3	KIAA0232	PDCD4	SCOC	CACNA1A	ALDH3B2	PCM1
ALDH3B2	DMD	KIAA1407	PDE3A	SEC16B	CACNA2D3	ALMS1	PCSK6
ALMS1	DNAH5	KLHDC7A	PDE9A	SETBP1	CNTN4	ARHGAP28	PDCD4
ANO4	DST	KLHL1	PDGFD	SFMBT2	CSMD1	ATP8B4	PDE9A
ARHGAP28	ESRRB	KSR2	PDXK	SLC12A2	CTNND2	C20orf96	PDGFD
ATP8B4	EXOSC1	LDLRAD3	PDZD2	SLC22A23	DMD	CACNA1A	PDZD2
ATXN3	FAM216A	LIN52	PEX13	SLC25A21	DST	CACNA2D3	PKNOX2
BBIP1	FAM89A	LPHN3	PGAM1P5	SLC39A11	ESRRB	CDK14	PLCG2
C20orf96	FAT4	LRP11	PIP5K1B	SLC5A12	FER	CSMD1	PRKAG2
CACNA1A	FER	LRP1B	PIP5K1C	SLIT3	FHIT	CSMD3	PRKCE
CACNA2D3	FERMT1	MAL2	PKNOX2	SMC6	GABRB1	CTNND2	PRPF4B
CACNG2	FHIT	MARCH1	PLCG2	SNTG2	GRIP1	DCC	PSD3
CARD6	FLIP1L	MAST4	POU2F3	SNX9	MCPH1	DMD	RBMS3
CCDC102B	FREM1	MCPH1	PPARGC1A	SORCS2	NELL1	DNAH5	RHBDF2
CCDC111	GABRB1	MIPEP	PRKAG2	SYT9	NTNG1	DST	RNF213
CCDC176	GALC	MPP7	PRKCE	TBCE	NXPH1	ESRRB	ROBO1
CCDC3	GAS7	MYO15B	PRKG1	TMEM182	PSD3	FAT4	RPH3AL
CD36	GNN	MYOM3	PRPF4B	TMTC2	RBMS3	FERMT1	SCOC
CDK14	GPR124	NCOR2	PSD3	TRIP11	ROBO1	GALC	SEC16B
CDKAL1	GPR125	NELL1	QRTRTD1	TTC7A	SETBP1	HEG1	SETBP1
CLYBL	GRIP1	NMNAT2	RABGAP1L	TUBGCP5	SLC39A11	KIAA0232	SLC22A23
CMSS1	HAL	NRDE2	RASGEF1B	VIT	SLIT3	KIAA1407	SLC39A11
CNOT10	HEG1	NTNG1	RBMS3	XRCC4	SNTG2	LRP11	SMC6
CNTN4	HSPA12A	NXPH1	RCN1	YIPF5	TUBGCP5	LRP1B	TMTC2
CSMD1	HVCN1	OAS2	RGS17	ZDDGGC16		MCPH1	TRIP11
CSMD3	IFT81	OR1J2	RHBDF2			MYOM3	TTC7A
CTNND2	IRS1	OR8U8	RNF180			NCOR2	TUBGCP5
CWC27	KCNIP4	PALLD	RNF213			NELL1	YIPF5
CYMP	KCNN3	PCM1	ROBO1			NTNG1	

* Q indicates probability of enrichment in SFARI or exome-seq genes from hypergeometric distribution analysis. ^#^ Exome-seq genes were downloaded from study by Iossifov et al. [23].

**Table 13:** Neurological functions of SNP-associated genes from dA.DZ vs. dNA.DZ case-control association analysis.

Function Annotation	*p*-Value *	Genes
development of neurons	5.50 × 10^−^^6^	*ADGRL3, CACNA1A, CACNG2, CNTN4, CTNND2, DCC, DMD, FER, GAS7, KLHL1, NTNG1, PALLD, PCM1, PIP5K1B, PIP5K1C, PRKCE, PRKG1, ROBO1, SLC12A2, SLIT3, TBCE*
neuritogenesis	3.57 × 10^−^^5^	*CACNA1A, CNTN4, CTNND2, DCC, FER, GAS7, KLHL1, NTNG1, PALLD, PIP5K1B, PIP5K1C, PRKCE, PRKG1, ROBO1, SLIT3, TBCE*
axonogenesis	2.58 × 10^−^^4^	*CACNA1A, CNTN4, DCC, NTNG1, PIP5K1C, PRKG1, ROBO1, TBCE*
migration of neurons	6.40 × 10^−^^4^	*ADGRL3, DCC, PCM1, PEX13, PIP5K1C, PRKG1, ROBO1, SLC12A2*
development of sensory projections	7.83 × 10^−^^4^	*ROBO1, SLIT3*
pathfinding of neurons	2.02 × 10^−^^3^	*DCC, ROBO1*
morphology of nervous system	2.62 × 10^−^^3^	*ADGRA2, BBIP1, CACNA1A, CNTN4, DCC, KLHL1, MCPH1, NCOR2, PALLD, PCSK6, PEX13, PPARGC1A, ROBO1, SLC12A2, XRCC4*
guidance of axons	4.61 × 10^−^^3^	*CNTN4, DCC, PRKG1, ROBO1, SLIT3*
abnormal morphology of forebrain	4.80 × 10^−^^3^	*ADGRA2, BBIP1, DCC, NCOR2, PPARGC1A, ROBO1*
formation of dendrites	4.83 × 10^−^^3^	*CACNA1A, GAS7, KLHL1, PRKG1, ROBO1*
developmental process of synapse	5.28 × 10^−^^3^	*ADGRL3, CACNA1A, CACNG2, DCC, DMD, SLC12A2*
quantity of oxytocin neurons	6.19 × 10^−^^3^	*DCC*
synaptic transmission of stellate cells	6.19 × 10^−^^3^	*CACNG2*
guidance of corticocortical axons	1.23 × 10^−^^2^	*ROBO1*
quantity of vasopressin neurons	1.23 × 10^−^^2^	*DCC*
activation of Purkinje cells	1.23 × 10^−^^2^	*CACNA1A*
afterhyperpolarization of dopaminergic neurons	1.23 × 10^−^^2^	*KCNN3*
size of hippocampal commissure	1.23 × 10^−^^2^	*ROBO1*
startle response	1.74 × 10^−^^2^	*CACNA2D3, CSMD1, PPARGC1A*
inhibition of presympathetic neurons	1.85 × 10^−^^2^	*SLC12A2*
fragmentation of myelin sheath	1.85 × 10^−^^2^	*GALC*
maturation of cerebellum	1.85 × 10^−^^2^	*CACNA1A*
turnover of synaptic vesicles	1.85 × 10^−^^2^	*CACNA1A*
density of synapse	2.20 × 10^−^^2^	*CTNND2, GRIP1*
morphogenesis of neurites	2.43 × 10^−^^2^	*CACNA1A, CNTN4, CTNND2, FER, GAS7, PALLD, PRKG1, ROBO1*

* Fisher exact *p*-value which indicates the probability that the list of SNP-associated genes is not enriched for annotated function, using genes in IPA’s Knowledgebase as the reference set of genes.

## Supplementary Materials

Supplementary materials can be found at https://www.mdpi.com/1422-0067/20/15/3804/s1, Table S1: All trait QTNs for cMZ twins, Table S2: All trait QTNs for dA.MZ twins, Table S3: All trait QTNs for dNA.MZ twins, Table S4: All trait QTNs for cDZ twins, Table S5: All trait QTNs for dA.DZ twins, Table S6: All trait QTNs for dNA.DZ twins, Table S7: Case-control by quartile analysis of cMZ twins, Table S8: Case-control by quartile analysis of dA.MZ twins, Table S9: Case-control by quartile analysis of dNA.MZ twins, Table S10: Case-control by quartile analysis of cDZ twins, Table S11: Case-control by quartile analysis of dA.DZ twins, Table S12: Case-control by quartile analysis of dNA.DZ twins, Table S13: Case-control analysis using dA.DZ as cases and dNA.DZ as controls, Table S14: Demographic information for all twins from AGRE repository, Table S15: ADI-R items used for quantitative trait severity profiles and analyses.
