# Supplementary material for: ASD Phenotype—Genotype Associations in Concordant and Discordant Monozygotic and Dizygotic Twins Stratified by Severity of Autistic Traits
Source: Int J Mol Sci. 2019 Aug 3;20(15):3804. doi: 10.3390/ijms20153804 (PMC6696087; doi:10.3390/ijms20153804)
Supplement: Supplementary file 1 [file ijms-20-03804-s001.zip › Supplementary Materials/Table S1. All trait QTNs for cMZ twins.pdf]

All trait QTNs for cMZ twins								
**Language**								
CHR	SNP	BP	P	Band	Genes	Role	aa Position	aa Change
6	rs6597379	9000801	8.75E-07	6p24.3				
6	rs7767901	9014658	8.75E-07	6p24.3				
8	rs4481588	35609723	8.00E-06	8p12	UNC5D	Intron	-	-
5	rs2546481	114841181	8.31E-06	5q22.3				
**Nonverbal**								
CHR	SNP	BP	P	Band	Genes	Role	aa Position	aa Change
5	rs2216904	51769238	1.08E-06	5q11.2				
4	rs13132286	20972739	1.43E-06	4p15.2	KCNIP4	Intron	-	-
4	rs893929	144406830	2.00E-06	4q31.21				
14	rs7145220	34193684	3.50E-06	14q13.1				
1	rs10925300	235283523	4.18E-06	1q43	RYR2	Intron	-	-
22	rs2073454	20847479	4.21E-06	22q11.22				
7	rs10235680	26875121	4.83E-06	7p15.2	SKAP2	Promoter	-	-
13	rs2221406	88972527	5.21E-06	13q31.3				
3	rs1554125	58252541	5.26E-06	3p14.3	ABHD6	Intron	-	-
4	rs17686361	144427136	6.59E-06	4q31.21				
13	rs1886215	41847359	8.11E-06	13q14.11				
8	rs359782	47302891	9.88E-06	8q11.1				
**Play**								
CHR	SNP	BP	P	Band	Genes	Role	aa Position	aa Change
1	rs497576	238092600	2.33E-07	1q43	CHRM3	Intron	-	-
2	rs1866472	129310314	3.36E-07	2q14.3				
4	rs13130915	173703447	2.57E-06	4q34.1	GALNTL6	Intron	-	-
5	rs357519	87546610	4.30E-06	5q14.3	TMEM161B	Intron	-	-
3	rs347134	32414789	9.55E-06	3p22.3	CMTM7	Intron	-	-

**Social**								
CHR	SNP	BP	P	Band	Genes	Role	aa Position	aa Change
8	rs7836486	47062007	1.60E-06	q11.1				
8	rs359782	47302891	1.65E-06	q11.1				
7	rs10486903	88621188	1.85E-06	q21.13	ZNF804B	intronic		
7	rs7787455	88588409	2.18E-06	q21.13	ZNF804B	intronic		
5	rs6556348	157807809	2.43E-06	q33.3	RP11-542A14.	non-coding intronic		
7	rs7795594	36794360	2.44E-06	p14.2				
7	rs9986865	36807127	2.44E-06	p14.2				
8	rs10100027	73553339	3.20E-06	q13.3				
8	rs11136142	47240116	3.58E-06	q11.1				
8	rs4873076	47255301	3.58E-06	q11.1				
8	rs359807	47309786	3.58E-06	q11.1				
8	rs7827943	73568186	4.81E-06	q13.3				
10	rs4880592	3163581	5.13E-06	p15.2	PFKP	intronic		

**Perseverative Behaviors**								
CHR	SNP	BP	P	Band	Genes	Role	aa Position	
5	rs324454	55238308	2.40E-06	12p13.1	APOLD1	Downstream	-	
12	rs2058604	12841126	3.71E-06	5q11.2	IL31RA	Intron	-	
11	rs4930028	2889641	4.46E-06	11p15.4	SLC22A18	Intron	-	
11	rs7926034	90154444	8.39E-06	11q14.3				
