# Supplementary material for: ASD Phenotype—Genotype Associations in Concordant and Discordant Monozygotic and Dizygotic Twins Stratified by Severity of Autistic Traits
Source: Int J Mol Sci. 2019 Aug 3;20(15):3804. doi: 10.3390/ijms20153804 (PMC6696087; doi:10.3390/ijms20153804)
Supplement: Supplementary file 1 [file ijms-20-03804-s001.zip › Supplementary Materials/Table S10. Case-control by quartile analysis of cDZ twins.pdf]

Summary of case-control by quartile analysis of cDZ twins

Language

Q1 (N = 9)	CHR	SNP	BP	A1	F_A	F_U	A2	CHISQ	OR	UNADJ P	BONF	FDR_BH	Band	Allele(s)	Gene	Location
	14	rs17127374	91017796	C	0.500	0.083	T	40.36	11.06	2.11E-10	5.49E-09	5.49E-09	14q32.12	C/T	SMEK1	Coding exon
	5	rs1559286	42787794	G	0.389	0.054	T	38.52	11.14	5.42E-10	1.41E-08	7.04E-09	5p12	G/T	CCDC152	Promoter
	2	rs17543960	116586663	G	0.444	0.071	T	37.04	10.42	1.16E-09	3.00E-08	1.00E-08	2q14.1	G/T		
	2	rs13425437	116550707	G	0.444	0.092	T	26.47	7.927	2.68E-07	6.97E-06	1.74E-06	2q14.1	G/T		
	21	rs2837366	40301653	T	0.500	0.169	C	13.91	4.917	1.92E-04	4.98E-03	9.97E-04	21q22.2	C/T	DSCAM	Downstream
	4	rs10520554	184446191	C	0.833	0.415	T	12.95	7.063	3.20E-04	8.32E-03	1.39E-03	4q35.1	C/T	WWC2	Intron
	3	rs11917359	25673708	A	0.333	0.093	G	12.23	4.889	4.71E-04	1.23E-02	1.75E-03	3p24.2	A/G	TOP2B	Intron
	13	rs1887263	22381716	A	0.500	0.187	G	11.56	4.362	6.73E-04	1.75E-02	2.19E-03	13q12.12	A/G		
	10	rs10827492	35469831	T	0.000	0.361	C	10.15	0	1.44E-03	3.75E-02	3.84E-03	10p11.21	C/T	CREM	Intron
	10	rs4934540	35514705	C	0.000	0.360	T	10.11	0	1.48E-03	3.84E-02	3.84E-03	10p11.21	C/T	CREM	Intron
	15	rs7177755	60886781	C	0.611	0.278	T	9.852	4.072	1.70E-03	4.41E-02	4.01E-03	15q22.2	C/T	TLN2	Intron
	16	rs4011585	71914015	A	0.111	0.472	C	9.391	0.1397	2.18E-03	5.67E-02	4.73E-03	16q22.3	A/C		
	16	rs4888646	71920088	A	0.111	0.457	G	8.636	0.1487	3.30E-03	8.57E-02	6.59E-03	16q22.3	A/G		
	7	rs2106740	89092149	T	0.375	0.133	C	8.093	3.922	4.45E-03	1.16E-01	8.26E-03	7q21.13	C/T		
	7	rs2058002	25406658	C	0.333	0.122	T	7.397	3.587	6.54E-03	1.70E-01	1.12E-02	7p15.3	C/T		
	21	rs2026886	45655301	C	0.111	0.424	A	7.191	0.1699	7.33E-03	1.91E-01	1.12E-02	21q22.3	A/C	NCRNA001	Downstream
	16	rs1886714	19970009	C	0.667	0.363	T	7.16	3.514	7.46E-03	1.94E-01	1.12E-02	16p12.3	C/T	GPR139	Intron
	18	rs4891775	65627430	C	0.625	0.315	T	7.081	3.623	7.79E-03	2.03E-01	1.12E-02				
	2	rs2889606	105921422	G	0.056	0.354	A	6.992	0.1074	8.19E-03	2.13E-01	1.12E-02	2q12.2	A/G		
	5	rs2578500	5515148	G	0.056	0.322	A	5.836	0.1239	1.57E-02	4.08E-01	2.04E-02	5p15.32	A/G	KIAA0947	Coding exon
	7	rs11975137	88988845	G	0.333	0.138	A	5.689	3.113	1.71E-02	4.44E-01	2.11E-02	7q21.13	A/G		
	7	rs7794184	89052607	C	0.333	0.145	T	5.106	2.947	2.38E-02	6.20E-01	2.82E-02	7q21.13	C/T		
	5	rs2578539	5547739	C	0.063	0.325	A	5.009	0.1386	2.52E-02	6.56E-01	2.85E-02	5p15.32	A/C	KIAA0947	Downstream
	15	rs7162186	30973508	T	0.389	0.196	C	4.231	2.613	3.97E-02	1.00E+00	4.30E-02	15q13.3	C/T	FMN1	Intron

Q4 (N = 28)	CHR	SNP	BP	A1	F_A	F_U	A2	CHISQ	OR	UNADJ P	BONF	FDR_BH	Band	Allele(s)	Gene	Location
	11	rs184267	1945152	A	0.089	0.378	G	19.69	0.1614	9.12E-06	2.37E-04	2.37E-04	11p15.5	A/G		
	5	rs2578539	5547739	C	0.589	0.325	A	17.57	2.983	2.77E-05	7.19E-04	3.40E-04	5p15.32	A/C	KIAA0947	Downstream
	16	rs4888646	71920088	A	0.732	0.457	G	16.91	3.252	3.92E-05	1.02E-03	3.40E-04	16q22.3	A/G		
	1	rs357044	178506096	G	0.268	0.105	A	15.28	3.107	9.27E-05	2.41E-03	5.63E-04	1q25.2	A/G	LHX4	Intron
	16	rs4011585	71914015	A	0.732	0.472	C	14.99	3.054	1.08E-04	2.81E-03	5.63E-04	16q22.3	A/C		
	16	rs1886714	19970009	C	0.143	0.363	T	11.61	0.2929	6.56E-04	1.71E-02	2.62E-03	16p12.3	C/T	GPR139	Intron
	18	rs4891775	65627430	C	0.096	0.315	T	11.47	0.2312	7.09E-04	1.84E-02	2.62E-03	NA			
	15	rs7162186	30973508	T	0.018	0.196	C	11.23	0.0747	8.07E-04	2.10E-02	2.62E-03	15q13.3	C/T	FMN1	Intron
	4	rs10520554	184446191	C	0.196	0.415	T	10.87	0.3453	9.80E-04	2.55E-02	2.83E-03	4q35.1	C/T	WWC2	Intron
	21	rs2837366	40301653	T	0.018	0.169	C	9.079	0.0894	2.59E-03	6.72E-02	6.72E-03	21q22.2	C/T	DSCAM	Downstream
	7	rs2058002	25406658	C	0.000	0.122	T	7.793	0	5.25E-03	1.36E-01	1.24E-02	7p15.3	C/T		
	5	rs2578500	5515148	G	0.482	0.322	A	6.499	1.962	1.08E-02	2.81E-01	2.17E-02	5p15.32	A/G	KIAA0947	Coding exon
	13	rs1887263	22381716	A	0.054	0.187	G	6.488	0.2469	1.09E-02	2.82E-01	2.17E-02	13q12.12	A/G		
	7	rs2106740	89092149	T	0.019	0.133	C	6.096	0.1233	1.36E-02	3.52E-01	2.52E-02	7q21.13	C/T		
	3	rs11917359	25673708	A	0.000	0.093	G	5.72	0	1.68E-02	4.36E-01	2.84E-02	3p24.2	A/G	TOP2B	Intron
	2	rs13425437	116550707	G	0.000	0.092	T	5.645	0	1.75E-02	4.55E-01	2.84E-02	2q14.1	G/T		
	15	rs7177755	60886781	C	0.143	0.278	T	5.083	0.4319	2.42E-02	6.28E-01	3.70E-02	15q22.2	C/T	TLN2	Intron
	2	rs17543960	116586663	G	0.000	0.071	T	4.296	0	3.82E-02	9.93E-01	5.52E-02	2q14.1	G/T		
	2	rs2889606	105921422	G	0.482	0.354	A	3.972	1.699	4.63E-02	1.00E+00	6.33E-02	2q12.2	A/G		
	7	rs7794184	89052607	C	0.054	0.145	T	3.759	0.3336	5.25E-02	1.00E+00	6.83E-02	7q21.13	C/T		
	7	rs11975137	88988845	G	0.054	0.138	A	3.361	0.3524	6.68E-02	1.00E+00	8.27E-02	7q21.13	A/G		
	5	rs1559286	42787794	G	0.000	0.054	T	3.196	0	7.38E-02	1.00E+00	8.73E-02	5p12	G/T	CCDC152	Promoter

Nonverbal

Q1 (N = 17)	CHR	SNP	BP	A1	F_A	F_U	A2	CHISQ	OR	UNADJ P	BONF	FDR_BH	Band	Alleles	Gene(s)	Role
Nonverb.Q1	14	rs11848377	36178269	C	0.059	0.204	T	4.387	0.2443	3.62E-02	7.24E-02	4.26E-02	14q13.3	C/T		
	14	rs12881439	36175604	G	0.059	0.198	A	4.11	0.254	4.26E-02	8.53E-02	4.26E-02	14q13.3	A/G		

Q4 (N = 11)	CHR	SNP	BP	A1	F_A	F_U	A2	CHISQ	OR	UNADJ P	BONF	FDR_BH	Band	Alleles	Gene(s)	Role
Nonverb.Q4	14	rs12881439	36175604	G	0.500	0.198	A	12.59	4.064	3.89E-04	7.78E-04	5.95E-04	14q13.3	A/G		
	14	rs11848377	36178269	C	0.500	0.204	T	11.79	3.908	5.95E-04	1.19E-03	5.95E-04	14q13.3	C/T		

Play

Q1 (N = 13)	CHR	SNP	BP	A1	F_A	F_U	A2	CHISQ	OR	UNADJ P	BONF	FDR_BH	Band	Alleles	Gene(s)	Role
Play.Q1	7	rs1180774	97475759	A	0.615	0.268	G	15.88	4.378	6.73E-05	7.41E-04	7.41E-04	7q21.3	A/G		
	6	rs9295192	162900893	T	0.000	0.339	G	12.27	0	4.59E-04	5.05E-03	2.53E-03	6q25.2-q2	G/T	PARK2	intron
	6	rs1407220	123934131	G	0.423	0.185	A	9.72	3.239	1.82E-03	2.01E-02	6.68E-03	6q22.31	A/G	TRDN	intron
	6	rs976145	81463977	T	0.385	0.167	C	8.746	3.116	3.10E-03	3.41E-02	7.18E-03	6q14.1	C/T		
	5	rs9329125	177334115	A	0.385	0.168	G	8.655	3.099	3.26E-03	3.59E-02	7.18E-03	5q35.3	A|G	RP11-1252	intron
	6	rs4707485	89294410	C	0.038	0.277	T	7.356	0.1045	6.68E-03	7.35E-02	1.23E-02	6q15	C/T	LOC10192	non-coding
	16	rs8060975	15784912	T	0.385	0.222	C	3.969	2.194	4.64E-02	5.10E-01	7.29E-02	16p13.11	C/T	MYH11	intron5upstream/3do
	6	rs2610763	89313184	C	0.038	0.177	T	3.433	0.1855	6.39E-02	7.03E-01	8.79E-02	6q15	C/T		
	2	rs6717613	299480	A	0.192	0.357	G	3.054	0.4291	8.05E-02	8.86E-01	9.84E-02	2p25.3	G|A	AC079779.	5upstream/intronic/3

Q4 (N = 15)	CHR	SNP	BP	A1	F_A	F_U	A2	CHISQ	OR	UNADJ P	BONF	FDR_BH	Band	Alleles	Gene(s)	Role
	2	rs6717613	299480	A	0.733	0.357	G	18.36	4.956	1.83E-05	2.01E-04	2.01E-04	2p25.3	G|A	AC079779.	5upstream/intronic/3
	10	rs1171582	61164043	T	0.800	0.432	C	16.46	5.266	4.96E-05	5.45E-04	2.73E-04	10q21.2	C|T	SLC16A9	intronic

	8	rs6982697	29938457	A	0.267	0.076	G	15.11	4.401	1.01E-04	1.11E-03	3.72E-04	8p12			
	6	rs2610763	89313184	C	0.433	0.177	T	13.29	3.546	2.67E-04	2.94E-03	7.34E-04	6q15	C/T		
	6	rs4707485	89294410	C	0.500	0.277	T	7.404	2.614	6.51E-03	7.16E-02	1.43E-02	6q15	C/T		
	6	rs1407220	123934131	G	0.000	0.185	A	6.782	0	9.21E-03	1.01E-01	1.69E-02	6q22.31	A/G	TRDN	intron
	7	rs1180774	97475759	A	0.067	0.268	G	6.163	0.1954	1.31E-02	1.44E-01	1.74E-02	7q21.3	A/G		
	5	rs9329125	177334115	A	0.000	0.168	G	6.043	0	1.40E-02	1.54E-01	1.74E-02	5q35.3	A|G	RP11-1252	intron
	6	rs976145	81463977	T	0.000	0.167	C	6.01	0	1.42E-02	1.56E-01	1.74E-02	6q14.1	C/T		
	16	rs8060975	15784912	T	0.067	0.222	C	4.165	0.2508	4.13E-02	4.54E-01	4.54E-02	16p13.11	C/T	MYH11	intron
	6	rs9295192	162900893	T	0.500	0.339	G	2.99	1.95	8.38E-02	9.21E-01	8.38E-02	6q25.2-q2	G/T	PARK2	intron

Social

Q1 (N = 18)	CHR	SNP	BP	A1	F_A	F_U	A2	CHISQ	OR	UNADJ P	BONF	FDR_BH	Band	Alleles	Gene(s)	Role
	15	rs4551980	47910160	T	0.028	0.320	C	14.09	0.0607	1.74E-04	1.22E-03	6.22E-04	15q21.2	C/T		
	15	rs7182178	47911691	T	0.028	0.320	C	14.05	0.0608	1.78E-04	1.24E-03	6.22E-04	15q21.2	C/T		
	18	rs6506251	4787073	G	0.639	0.378	A	10.35	2.915	1.30E-03	9.06E-03	3.02E-03	18p11.31	A/G		
	2	rs6717613	299480	A	0.167	0.357	G	5.642	0.3605	1.75E-02	1.23E-01	3.07E-02	2p25.3	A/G		
	17	rs2916154	62923036	G	0.471	0.296	A	4.924	2.114	2.65E-02	1.85E-01	3.30E-02	17q24.2	A/G	PITPNC1	Intron
	10	rs1171582	61164043	T	0.250	0.432	C	4.813	0.4388	2.83E-02	1.98E-01	3.30E-02	10q21.2	C/T		

Q4 (N = 11)	CHR	SNP	BP	A1	F_A	F_U	A2	CHISQ	OR	UNADJ P	BONF	FDR_BH	Band	Alleles	Gene(s)	Role
Social.Q4	18	rs352135	48086130	G	0.636	0.211	A	23.67	6.548	1.14E-06	8.01E-06	8.01E-06	18q21.2	A/G		
	2	rs6717613	299480	A	0.818	0.357	G	20.26	8.11	6.77E-06	4.74E-05	2.37E-05	2p25.3	A/G		
	10	rs1171582	61164043	T	0.818	0.432	C	13.32	5.924	2.63E-04	1.84E-03	6.12E-04	10q21.2	C/T		
	18	rs6506251	4787073	G	0.045	0.378	A	10.3	0.0785	1.33E-03	9.30E-03	2.32E-03	18p11.31	A/G		
	17	rs2916154	62923036	G	0.000	0.296	A	9.231	0	2.38E-03	1.67E-02	3.33E-03	17q24.2	A/G	PITPNC1	Intron
	15	rs7182178	47911691	T	0.591	0.320	C	7.39	3.073	6.56E-03	4.59E-02	6.67E-03	15q21.2	C/T		
	15	rs4551980	47910160	T	0.591	0.320	C	7.359	3.067	6.67E-03	4.67E-02	6.67E-03	15q21.2	C/T		

Perseverative Behavior (Sameness)

Q1 (N = 19)	CHR	SNP	BP	A1	F_A	F_U	A2	CHISQ	OR	UNADJ P	BONF	FDR_BH	Band	Alleles	Gene(s)	Role
	1	rs7547507	15494295	T	0.632	0.363	G	11.78	3.015	5.98E-04	8.97E-03	8.50E-03	1p36.21	G/T	FHAD1	Intron
	18	rs662397	42574834	G	0.026	0.258	T	10.6	0.0779	1.13E-03	1.70E-02	8.50E-03	18q21.1	G/T	ST8SIA5	Intron
	18	rs9675544	42570717	A	0.026	0.204	G	7.348	0.1057	6.71E-03	1.01E-01	3.36E-02	18q21.1	A/G	ST8SIA5	Intron
	14	rs8014901	68159378	C	0.053	0.212	T	5.776	0.2061	1.63E-02	2.44E-01	4.14E-02	14q24.1	C/T		
	14	rs2331779	68151670	T	0.026	0.173	G	5.687	0.1294	1.71E-02	2.56E-01	4.14E-02	14q24.1	G/T		
	6	rs1040521	2984608	A	0.263	0.453	G	5.496	0.431	1.91E-02	2.86E-01	4.14E-02	6p25.2	A/G	FAM136B	Promoter
	20	rs6039104	8164697	A	0.026	0.169	G	5.475	0.1333	1.93E-02	2.89E-01	4.14E-02	20p12.3	A/G	PLCB1	Intron
	11	rs12146477	102130166	G	0.105	0.268	A	5.082	0.3221	2.42E-02	3.63E-01	4.53E-02	11q22.2	A/G		
	6	rs7774283	2986199	C	0.263	0.441	T	4.858	0.4521	2.75E-02	4.13E-01	4.59E-02	6p25.2	C/T	FAM136B	Promoter
	13	rs9562157	113999458	A	0.184	0.351	G	4.595	0.4182	3.21E-02	4.81E-01	4.81E-02	13q34	A/G		
	8	rs4875505	5149306	T	0.316	0.474	C	3.774	0.5127	5.21E-02	7.81E-01	7.10E-02	8p23.2	C/T		
	11	rs2846714	102031820	G	0.105	0.234	T	3.504	0.3847	6.12E-02	9.18E-01	7.52E-02	11q22.2	G/T		
	4	rs12500644	138941802	T	0.211	0.354	C	3.402	0.4865	6.51E-02	9.77E-01	7.52E-02	4q28.3	C/T		

Q4 (N = 14)	CHR	SNP	BP	A1	F_A	F_U	A2	CHISQ	OR	UNADJ P	BONF	FDR_BH	Band	Alleles	Gene(s)	Role
	11	rs2846714	102031820	G	0.654	0.234	T	25.23	6.177	5.09E-07	7.63E-06	7.63E-06	11q22.2	G/T		
	4	rs12500644	138941802	T	0.714	0.354	C	15.75	4.561	7.21E-05	1.08E-03	3.72E-04	4q28.3	C/T		
	21	rs7281374	34423844	G	0.308	0.087	A	15.7	4.67	7.44E-05	1.12E-03	3.72E-04	21q22.11	A/G	MRPS6	Intron
	13	rs9562157	113999458	A	0.679	0.351	G	13.12	3.91	2.93E-04	4.39E-03	1.10E-03	13q34	A/G		
	14	rs2331779	68151670	T	0.429	0.173	G	12.66	3.591	3.73E-04	5.59E-03	1.12E-03	14q24.1	G/T		
	14	rs8014901	68159378	C	0.464	0.212	T	10.51	3.215	1.19E-03	1.78E-02	2.66E-03	14q24.1	C/T		
	9	rs1340007	1314127	G	0.654	0.353	A	10.27	3.47	1.35E-03	2.03E-02	2.66E-03	9p24.3	A/G		
	11	rs12146477	102130166	G	0.536	0.268	A	10.18	3.159	1.42E-03	2.13E-02	2.66E-03	11q22.2	A/G		
	20	rs6039104	8164697	A	0.393	0.169	G	9.925	3.191	1.63E-03	2.45E-02	2.72E-03	20p12.3	A/G	PLCB1	Intron
	8	rs4875505	5149306	T	0.750	0.474	C	8.521	3.333	3.51E-03	5.27E-02	5.27E-03	8p23.2	C/T		
	6	rs7774283	2986199	C	0.679	0.441	T	6.351	2.672	1.17E-02	1.76E-01	1.60E-02	6p25.2	C/T	FAM136B	Promoter
	18	rs9675544	42570717	A	0.393	0.204	G	6.121	2.53	1.34E-02	2.00E-01	1.67E-02	18q21.1	A/G	ST8SIA5	Intron
	6	rs1040521	2984608	A	0.679	0.453	G	5.705	2.547	1.69E-02	2.54E-01	1.92E-02	6p25.2	A/G	FAM136B	Promoter
	18	rs662397	42574834	G	0.462	0.258	T	5.608	2.47	1.79E-02	2.68E-01	1.92E-02	18q21.1	G/T	ST8SIA5	Intron
	1	rs7547507	15494295	T	0.179	0.363	G	4.081	0.3823	4.34E-02	6.50E-01	4.34E-02	1p36.21	G/T	FHAD1	Intron
