# Supplementary material for: ASD Phenotype—Genotype Associations in Concordant and Discordant Monozygotic and Dizygotic Twins Stratified by Severity of Autistic Traits
Source: Int J Mol Sci. 2019 Aug 3;20(15):3804. doi: 10.3390/ijms20153804 (PMC6696087; doi:10.3390/ijms20153804)
Supplement: Supplementary file 1 [file ijms-20-03804-s001.zip › Supplementary Materials/Table S11. Case-control by quartile analysis of dA.DZ twins.pdf]

Summary of case-control by quartile analysis of dA.DZ twins

Language

Q1 (N = 12)	CHR	SNP	BP	A1	F_A	F_U	A2	CHISQ	OR	UNADJ	BONF	FDR_BH	Band	Alleles	Gene	Role
	12	rs249047	112562237	G	0.083	0.422	A	11.26	0.1243	7.90E-04	2.37E-03	2.37E-03	12q24.13			
	15	rs1823718	71934297	C	0.708	0.470	T	5.453	2.74	1.95E-02	5.86E-02	2.93E-02	15q24.1			
	1	rs17587765	117372602	T	0.042	0.185	G	3.266	0.1915	7.07E-02	2.12E-01	7.07E-02	1p13.1	G/T	CD101	intronic

Q4 (N = 12)	CHR	SNP	BP	A1	F_A	F_U	A2	CHISQ	OR	UNADJ	BONF	FDR_BH	Band	Alleles	Gene	Role
	1	rs17587765	117372602	T	0.542	0.185	G	19.99	5.204	7.78E-06	2.33E-05	2.33E-05	1p13.1	G/T	CD101	intronic
	15	rs1823718	71934297	C	0.125	0.470	T	11.41	0.1612	7.32E-04	2.20E-03	1.10E-03	15q24.1			
	12	rs249047	112562237	G	0.625	0.422	A	4.016	2.279	4.51E-02	1.35E-01	4.51E-02	12q24.13			

Nonverbal

Q1 (N = 11)	CHR	SNP	BP	A1	F_A	F_U	A2	CHISQ	OR	UNADJ	BONF	FDR_BH	Band	Alleles	Gene	Role
	6	rs6923735	3362642	A	0.136	0.402	G	6.446	0.2346	1.11E-02	4.45E-02	1.58E-02	6p25.2	G|A	SLC22A23	intronic
	11	rs2953310	40094119	C	0.182	0.450	T	6.355	0.2719	1.17E-02	4.68E-02	1.58E-02	11p12	T|C	LRRC4C	coding
	6	rs807302	119968450	C	0.000	0.221	A	6.221	0	1.26E-02	5.05E-02	1.58E-02	6q22.31			
	3	rs7613095	194294915	G	0.045	0.276	A	5.83	0.125	1.58E-02	6.30E-02	1.58E-02	3q29			

Q4 (N = 13)	CHR	SNP	BP	A1	F_A	F_U	A2	CHISQ	OR	UNADJ	BONF	FDR_BH	Band	Alleles	Gene	Role
	6	rs6923735	3362642	A	0.692	0.402	G	9.037	3.343	2.65E-03	1.06E-02	8.16E-03	6p25.2	G|A	SLC22A23	intronic
	11	rs2953310	40094119	C	0.731	0.450	T	8.249	3.321	4.08E-03	1.63E-02	8.16E-03	11p12	T|C	LRRC4C	coding
	3	rs7613095	194294915	G	0.500	0.276	A	6.489	2.626	1.09E-02	4.34E-02	1.45E-02	3q29			

Play

Q4 (N = 9)	CHR	SNP	BP	A1	F_A	F_U	A2	CHISQ	OR	UNADJ	BONF	FDR_BH	Band	Alleles	Gene	Role
	12	rs17483048	118415784	G	0.556	0.096	A	42.72	11.71	6.32E-11	3.16E-10	3.16E-10	12q24.23	A|G	CCDC60/A	non-coding intronic
	3	rs1563981	28722162	C	0.375	0.107	A	11.86	4.998	5.74E-04	2.87E-03	1.43E-03	3p24.1	T|G	LINC00693	non-coding intronic
	10	rs17692734	54841584	C	0.444	0.164	T	10.29	4.093	1.33E-03	6.67E-03	2.22E-03	10q21.1			
	10	rs2133470	54807327	G	0.389	0.138	A	9.508	3.993	2.05E-03	1.02E-02	2.56E-03	10q21.1			
	16	rs17772954	76243191	A	0.389	0.165	G	6.546	3.233	1.05E-02	5.26E-02	1.05E-02	16q23.1			

Social

Q1 (N = 9)	CHR	SNP	BP	A1	F_A	F_U	A2	CHISQ	OR	UNADJ	BONF	FDR_BH	Band	Alleles	Gene	Role
	11	rs2953310	40094119	C	0.167	0.450	T	5.809	0.2447	1.59E-02	3.19E-02	2.49E-02	11p12	T/C	LRRC4C	coding
	14	rs12894797	79861843	T	0.722	0.458	C	5.029	3.073	2.49E-02	4.99E-02	2.49E-02	14q31.1	C/T	DIO2-AS1	

Q4 (N = 12)	CHR	SNP	BP	A1	F_A	F_U	A2	CHISQ	OR	UNADJ	BONF	FDR_BH	Band	Alleles	Gene	Role
	11	rs2953310	40094119	C	0.750	0.450	T	8.696	3.671	3.19E-03	6.38E-03	6.38E-03	11p12	T/C	LRRC4C	coding
	14	rs12894797	79861843	T	0.250	0.458	C	4.177	0.3939	4.10E-02	8.20E-02	4.10E-02	14q31.1	C/T	DIO2-AS1	

Perseverative Behaviors

Q1 (N = 19)	CHR	SNP	BP	A1	F_A	F_U	A2	CHISQ	OR	UNADJ	BONF	FDR_BH	Band	Alleles	Gene	Role
	6	rs6454752	90075080	T	0.053	0.237	C	7.124	0.1787	7.61E-03	5.33E-02	2.24E-02	6q15	C/T	GABRR2	intronic
	10	rs12570126	75086795	G	0.026	0.196	A	6.919	0.1109	8.53E-03	5.97E-02	2.24E-02	10q22.2	A|G	SYNPO2L	5upstream/noncoding intronic
	17	rs1010045	67801683	T	0.000	0.150	C	6.712	0	9.58E-03	6.70E-02	2.24E-02	17q24.3			
	10	rs11818084	125976888	A	0.132	0.286	G	4.41	0.3784	3.57E-02	2.50E-01	6.25E-02	10q26.13			

Q4 (N = 6)	CHR	SNP	BP	A1	F_A	F_U	A2	CHISQ	OR	UNADJ	BONF	FDR_BH	Band	Alleles	Gene	Role
	18	rs8084650	36009793	G	0.417	0.045	A	38.22	15.33	6.31E-10	4.42E-09	4.42E-09	18q12.3			
	11	rs11219808	124225108	T	0.417	0.067	C	23.1	9.934	1.54E-06	1.08E-05	5.38E-06	11q24.2			
	10	rs12570126	75086795	G	0.583	0.196	A	11.37	5.747	7.48E-04	5.24E-03	1.75E-03	10q22.2	A|G	SYNPO2L	5upstream/noncoding intronic
	3	rs2679593	157706326	G	0.000	0.494	A	9.725	0	1.82E-03	1.27E-02	3.18E-03				
	10	rs11818084	125976888	A	0.667	0.286	G	8.483	4.995	3.59E-03	2.51E-02	5.02E-03	10q26.13			
	17	rs1010045	67801683	T	0.417	0.150	C	6.628	4.038	1.00E-02	7.03E-02	1.17E-02	17q24.3			
	6	rs6454752	90075080	T	0.500	0.237	C	4.565	3.217	3.26E-02	2.29E-01	3.26E-02	6q15	C/T	GABRR2	intronic
