# Supplementary material for: ASD Phenotype—Genotype Associations in Concordant and Discordant Monozygotic and Dizygotic Twins Stratified by Severity of Autistic Traits
Source: Int J Mol Sci. 2019 Aug 3;20(15):3804. doi: 10.3390/ijms20153804 (PMC6696087; doi:10.3390/ijms20153804)
Supplement: Supplementary file 1 [file ijms-20-03804-s001.zip › Supplementary Materials/Table S12. Case-control by quartile analysis of dNA.DZ twins.pdf]

Summary of case-control by quartile analysis of dNA.DZ twins

Language

Q4 (N = 17)	CHR	SNP	BP	A1	F_A	F_U	A2	CHISQ	OR	UNADJ	BONF	FDR_BH	Band	Alleles	Gene(s)	Role
	8	rs17595454	18377451	T	0.281	0.081	C	16.8	4.429	4.16E-05	3.70E-03	3.70E-03	p22	C/T		

Nonverbal

Q4 (N = 15)	CHR	SNP	BP	A1	F_A	F_U	A2	CHISQ	OR	UNADJ	BONF	FDR_BH	Band	Alleles	Gene(s)	Role
	3	rs17564921	148372345	C	0.233	0.069	A	12.39	4.111	4.32E-04	3.93E-02	1.81E-02	q24	A|C	RP11-649A	intronic
	5	rs17211823	128158012	C	0.233	0.069	T	12.31	4.096	4.49E-04	4.09E-02	1.81E-02	q23.3	T|C	SLC27A6	intronic
	7	rs4719155	70745160	A	0.533	0.258	G	11.78	3.29	5.99E-04	5.45E-02	1.81E-02	q11.22	A|G	WBSCR17	intronic
	22	rs6005657	26565087	T	0.333	0.128	C	11.25	3.42	7.97E-04	7.25E-02	1.81E-02	q12.1			
	9	rs6479471	95084239	T	0.300	0.114	G	10.04	3.316	1.53E-03	1.39E-01	2.75E-02	q22.31	G|T	WNK2	intronic
	4	rs2342654	156369261	T	0.333	0.141	C	9.086	3.053	2.58E-03	2.34E-01	2.75E-02	q32.1			
	9	rs7022051	95042804	T	0.300	0.120	C	9.073	3.142	2.59E-03	2.36E-01	2.75E-02	q22.31	C|T	WNK2	intronic
	9	rs7857220	95064873	C	0.300	0.120	T	9.068	3.141	2.60E-03	2.37E-01	2.75E-02	q22.31	T|C	WNK2	intronic (5upstream)
	18	rs8084568	57190720	T	0.233	0.082	C	8.988	3.409	2.72E-03	2.47E-01	2.75E-02	q21.33	C|T	CDH20	intronic
	7	rs10224816	9678184	C	0.267	0.106	T	8.147	3.085	4.31E-03	3.93E-01	3.93E-02	p21.3			
	10	rs497204	80306297	A	0.167	0.052	G	7.935	3.668	4.85E-03	4.41E-01	4.01E-02	q22.3			
	10	rs772822	80304750	C	0.133	0.037	T	7.606	3.991	5.82E-03	5.29E-01	4.41E-02	q22.3			
	17	rs4387653	68659971	T	0.367	0.177	C	7.291	2.686	6.93E-03	6.31E-01	4.85E-02	q25.1			
	20	rs17805193	59138980	T	0.167	0.055	C	6.996	3.416	8.17E-03	7.43E-01	5.31E-02	q13.33			
	8	rs11984453	4491939	A	0.200	0.079	G	5.969	2.92	1.46E-02	1.00E+00	8.83E-02	p23.2	G|A	CSMD1	intronic

Play

Q4 (N = 16)	CHR	SNP	BP	A1	F_A	F_U	A2	CHISQ	OR	UNADJ	BONF	FDR_BH	Band	Alleles	Gene(s)	Role
	20	rs2427386	60794328	T	0.625	0.346	C	10.92	3.151	9.53E-04	1.67E-01	9.03E-02	q13.33			
	16	rs1801255	56247306	C	0.469	0.229	A	10.28	2.968	1.35E-03	2.35E-01	9.03E-02	q21	A|C	GPR56	coding
	6	rs853884	36576111	T	0.219	0.073	C	9.949	3.574	1.61E-03	2.82E-01	9.03E-02	p21.31	A|G	STK38	intronic
	1	rs2255340	230002773	A	0.469	0.243	G	8.738	2.744	3.12E-03	5.45E-01	9.03E-02	q42.2	T|C	DISC1	intronic
	13	rs2876807	61019136	G	0.188	0.062	A	8.576	3.508	3.41E-03	5.96E-01	9.03E-02	q21.2			
	2	rs10490706	19355330	C	0.406	0.199	T	8.512	2.752	3.53E-03	6.17E-01	9.03E-02	p24.1	T|C	AC092594.	intronic
	8	rs11984453	4491939	A	0.219	0.079	G	8.47	3.27	3.61E-03	6.32E-01	9.03E-02	p23.2	G|A	CSMD1	intronic
	2	rs11690021	239301967	C	0.219	0.080	T	8.191	3.212	4.21E-03	7.37E-01	9.21E-02	q37.3	T|C	AC113618.	intronic

Perseverative Behaviors

Q1 (N = 20)	CHR	SNP	BP	A1	F_A	F_U	A2	CHISQ	OR	UNADJ	BONF	FDR_BH	Band	Alleles	Gene(s)	Role
	12	rs2708079	41202340	A	0.125	0.408	G	13.19	0.2072	2.82E-04	7.32E-03	6.48E-03	q12	T|C	PRICKLE1	intronic
	9	rs7042145	8394095	T	0.750	0.474	C	12.12	3.33	4.99E-04	1.30E-02	6.48E-03	p24.1	T|C	PTPRD	intronic
	5	rs149473	123648735	C	0.000	0.169	T	8.145	0	4.32E-03	1.12E-01	3.74E-02	q23.2			
	4	rs2871491	186937857	A	0.000	0.153	G	7.218	0	7.22E-03	1.88E-01	4.20E-02	q35.1	G|A	SORBS2	intronic
	7	rs4337997	22351942	G	0.025	0.189	A	7.016	0.1098	8.08E-03	2.10E-01	4.20E-02	p15.3	A|G	RAPGEF5	intronic
	6	rs7756298	40808750	T	0.125	0.315	C	6.638	0.3111	9.98E-03	2.60E-01	4.33E-02	p21.1			
	6	rs12529308	40797708	A	0.125	0.301	C	5.877	0.3312	1.53E-02	3.99E-01	5.68E-02	p21.1			
	9	rs3780457	99962228	T	0.000	0.124	C	5.649	0	1.75E-02	4.54E-01	5.68E-02	q22.33	G|A	CORO2A	intronic
	7	rs12333620	22324730	T	0.075	0.218	G	4.772	0.291	2.89E-02	7.52E-01	7.26E-02	p15.3	G|T	RAPGEF5	intronic
	7	rs1568571	22341449	G	0.075	0.218	A	4.751	0.2917	2.93E-02	7.61E-01	7.26E-02	p15.3	T|C	RAPGEF5	intronic
	2	rs6745182	190533471	C	0.000	0.105	T	4.668	0	3.07E-02	7.99E-01	7.26E-02	q32.2	T|C	C2orf88	intronic
	5	rs10059061	95806928	C	0.000	0.097	A	4.313	0	3.78E-02	9.84E-01	8.20E-02	q15	A|C	CTD-2337A12.1	
	6	rs6912147	22371817	G	0.000	0.094	A	4.164	0	4.13E-02	1.00E+00	8.26E-02	p22.3	G|A	RP3-404K8	intronic
	5	rs1870658	73612235	C	0.000	0.086	T	3.738	0	5.32E-02	1.00E+00	9.22E-02	q13.3			
	5	rs471405	73613440	A	0.000	0.086	G	3.738	0	5.32E-02	1.00E+00	9.22E-02	q13.3			

Q4 (N = 15)	CHR	SNP	BP	A1	F_A	F_U	A2	CHISQ	OR	UNADJ	BONF	FDR_BH	Band	Alleles	Gene(s)	Role
	6	rs12529308	40797708	A	0.600	0.301	C	12.59	3.477	3.89E-04	1.01E-02	5.26E-03	p21.1			
	7	rs4337997	22351942	G	0.433	0.189	A	11.49	3.275	7.00E-04	1.82E-02	5.26E-03	p15.3	A|G	RAPGEF5	intronic
	9	rs7042145	8394095	T	0.167	0.474	C	11.3	0.222	7.77E-04	2.02E-02	5.26E-03	p24.1	T|C	PTPRD	intronic
	6	rs7756298	40808750	T	0.600	0.315	C	11.22	3.266	8.10E-04	2.11E-02	5.26E-03	p21.1			
	12	rs2708079	41202340	A	0.700	0.408	G	10.5	3.384	1.19E-03	3.10E-02	6.21E-03	q12	T|C	PRICKLE1	intronic
	16	rs17261378	81231342	A	0.200	0.066	G	8.679	3.559	3.22E-03	8.37E-02	1.05E-02	q23.3	G|A	CDH13	intronic
	2	rs6745182	190533471	C	0.267	0.105	T	8.293	3.113	3.98E-03	1.03E-01	1.05E-02	q32.2	T|C	C2orf88	intronic
	5	rs1870658	73612235	C	0.233	0.086	T	8.251	3.254	4.07E-03	1.06E-01	1.05E-02	q13.3			
	5	rs471405	73613440	A	0.233	0.086	G	8.251	3.254	4.07E-03	1.06E-01	1.05E-02	q13.3			
	7	rs1568571	22341449	G	0.433	0.218	A	8.128	2.751	4.36E-03	1.13E-01	1.05E-02	p15.3	T|C	RAPGEF5	intronic
	7	rs12333620	22324730	T	0.433	0.218	G	8.086	2.745	4.46E-03	1.16E-01	1.05E-02	p15.3	G|T	RAPGEF5	intronic
	8	rs11784372	121589419	C	0.167	0.054	T	7.384	3.521	6.58E-03	1.71E-01	1.34E-02	q24.12	T|C	MTBP	intronic
	17	rs9894565	17047909	A	0.533	0.305	G	7.355	2.611	6.69E-03	1.74E-01	1.34E-02	p11.2	A|G	PLD6	intronic
	6	rs1233386	29666169	A	0.321	0.143	G	7.171	2.835	7.41E-03	1.93E-01	1.38E-02	p22.1	C|T	GABBR1	intronic
	3	rs6438175	115134904	C	0.167	0.063	T	5.46	2.997	1.95E-02	5.06E-01	3.37E-02	q13.31	T|C	GRAMD1C	intronic
	13	rs12874523	105487642	C	0.333	0.174	A	5.213	2.368	2.24E-02	5.83E-01	3.64E-02	q33.2			
	3	rs9310756	24767428	C	0.233	0.119	T	3.718	2.258	5.38E-02	1.00E+00	8.23E-02	p24.2	T|C	AC133680.	intronic
