# Supplementary material for: ASD Phenotype—Genotype Associations in Concordant and Discordant Monozygotic and Dizygotic Twins Stratified by Severity of Autistic Traits
Source: Int J Mol Sci. 2019 Aug 3;20(15):3804. doi: 10.3390/ijms20153804 (PMC6696087; doi:10.3390/ijms20153804)
Supplement: Supplementary file 1 [file ijms-20-03804-s001.zip › Supplementary Materials/Table S13. Case-control analysis using dA.DZ as cases and dNA.DZ as controls.pdf]

Case-control analysis using dA.DZ as cases and dNA.DZ as controls

CHR	SNP	BP	Band	A1	F_A	F_U	A2	CHISQ	P	OR	Symbol	Role
14	rs12883250	87516629	q31.3	A	0.5395	0.2778	C	10.45	1.23E-03	3.046	GALC	intronic
1	rs2480767	181523841	q25.3	T	0.3816	0.1528	C	9.814	1.73E-03	3.422	NMNAT2	intronic
1	rs4652795	181532805	q25.3	C	0.4474	0.2083	T	9.539	2.01E-03	3.076	NMNAT2	intronic
1	rs2494462	180534188	q25.3	C	0.3553	0.1389	T	9.232	2.38E-03	3.416	GS1_122H1.2	non-coding intronic
1	rs2494465	180531396	q25.3	G	0.3553	0.1389	A	9.232	2.38E-03	3.416	GS1_122H1.2	non-coding intronic
1	rs2985431	180536324	q25.3	C	0.3553	0.1389	A	9.232	2.38E-03	3.416	GS1_122H1.2	non-coding intronic
1	rs3001275	180535260	q25.3	T	0.3553	0.1389	C	9.232	2.38E-03	3.416	GS1_122H1.2	non-coding intronic
14	rs17124022	87601279	q31.3	C	0.4079	0.1806	T	9.147	2.49E-03	3.126	LOC283587	non-coding intronic
10	rs17415112	99194781	q24.1	A	0.1974	0.0417	G	8.39	3.77E-03	5.656	EXOSC1/ZDDGGC16	intronic/5UPSTREAM
1	rs2477185	180533140	q25.3	A	0.3429	0.1389	C	8.109	4.41E-03	3.235	GS1_122H1.2	non-coding intronic
5	rs2887029	39470981	p13.1	C	0.3378	0.1389	T	7.925	4.88E-03	3.163		
3	rs4401377	20621490	p24.3	A	0.2368	0.0694	G	7.893	4.96E-03	4.159		
22	rs4822872	26109272	q12.1	G	0.3158	0.1250	T	7.768	5.32E-03	3.231		
5	rs13184924	108130246	q21.3	G	0.2143	0.0556	A	7.716	5.47E-03	4.636	FER	intronic
2	rs6706314	82740073	p12	T	0.1351	0.0139	C	7.701	5.52E-03	11.09		
5	rs13169624	108099815	q21.3	T	0.2105	0.0556	G	7.597	5.85E-03	4.533		
23	rs2748316	32684964	p21.1	A	0.4222	0.1698	G	7.598	5.85E-03	3.573	DMD	intronic
2	rs13432506	47078591	p21	A	0.1579	0.0278	C	7.309	6.86E-03	6.562	TTC7A	intronic
14	rs7159781	98087040	q32.2	A	0.1579	0.0278	C	7.309	6.86E-03	6.562		
5	rs2398721	143568607	q31.3	T	0.2237	0.0694	C	6.95	8.38E-03	3.861	KCTD16	intronic
3	rs6774516	5277341	p26.1	G	0.3026	0.1250	T	6.884	8.70E-03	3.038		
14	rs7153606	90380798	q32.11	T	0.3026	0.1250	C	6.884	8.70E-03	3.038		
2	rs2160337	36818255	p22.2	C	0.2632	0.0972	A	6.826	8.99E-03	3.316	VIT	intronic
5	rs7728260	174361691	q35.2	C	0.2632	0.0972	T	6.826	8.99E-03	3.316		
5	rs10050395	64203306	q12.3	T	0.1974	0.0556	C	6.645	9.94E-03	4.18	CWC27	intronic
5	rs10059206	64142674	q12.3	A	0.1974	0.0556	G	6.645	9.94E-03	4.18	CWC27	intronic
23	rs2855699	32684449	p21.1	A	0.4000	0.1698	G	6.46	1.10E-02	3.259	DMD	intronic
11	rs16928480	36158286	p13	C	0.1711	0.0417	T	6.419	1.13E-02	4.746	LDLRAD3	intronic
13	rs2806283	99120688	q32.3	C	0.1711	0.0417	T	6.419	1.13E-02	4.746	CLYBL	intronic
5	rs4585482	120518563	q23.1	C	0.1711	0.0417	A	6.419	1.13E-02	4.746		
3	rs6768081	30503493	p24.1	A	0.1711	0.0417	G	6.419	1.13E-02	4.746		
2	rs2698189	61121796	p16.1	C	0.1216	0.0143	T	6.413	1.13E-02	9.554	PEX13	intronic
11	rs12360927	124709463	q24.2	A	0.1184	0.0139	G	6.413	1.13E-02	9.537	PKNOX2	intronic
13	rs12861751	60217996	q21.2	A	0.1184	0.0139	G	6.413	1.13E-02	9.537		
13	rs1572023	60232512	q21.2	A	0.1184	0.0139	G	6.413	1.13E-02	9.537		
1	rs17017931	107255126	p13.3	G	0.1184	0.0139	A	6.413	1.13E-02	9.537		
13	rs3119877	60279082	q21.2	G	0.1184	0.0139	A	6.413	1.13E-02	9.537		
10	rs353207	6823268	p14	C	0.1184	0.0139	T	6.413	1.13E-02	9.537		
10	rs353208	6823114	p14	C	0.1184	0.0139	T	6.413	1.13E-02	9.537		
8	rs437707	17909112	p22	G	0.1184	0.0139	A	6.413	1.13E-02	9.537	PCM1	intronic
1	rs4506448	229229266	q42.2	T	0.1184	0.0139	C	6.413	1.13E-02	9.537	FAM89A	intronic
2	rs6728021	32975008	p22.3	G	0.1184	0.0139	A	6.413	1.13E-02	9.537	LINC00486	intronic
5	rs6884996	168509507	q35.1	T	0.2368	0.0833	C	6.413	1.13E-02	3.414	SLIT3	intronic
18	rs12456004	64738587	q22.1	C	0.2763	0.1111	A	6.405	1.14E-02	3.055	CCDC102B	intronic
5	rs1445803	13931843	p15.2	A	0.2763	0.1111	C	6.405	1.14E-02	3.055	DNAH5	intronic
4	rs975477	139759112	q31.1	A	0.2763	0.1111	G	6.405	1.14E-02	3.055		
10	rs10998853	70976314	q22.1	A	0.1714	0.0417	G	6.326	1.19E-02	4.759		
11	rs11031538	31925200	p13	G	0.1447	0.0278	T	6.312	1.20E-02	5.923	RCN1	intronic
3	rs11130783	60736055	p14.2	T	0.1447	0.0278	C	6.312	1.20E-02	5.923	FHIT	intronic
4	rs1398043	34422303	p15.1	A	0.1447	0.0278	C	6.312	1.20E-02	5.923		
4	rs1511862	34434019	p15.1	C	0.1447	0.0278	T	6.312	1.20E-02	5.923		
11	rs547008	121523071	q24.1	T	0.1447	0.0278	C	6.312	1.20E-02	5.923	MIRLET7A2/MIR100HG	5upstream/intronic
4	rs6815830	14516033	p15.33	C	0.1447	0.0278	T	6.312	1.20E-02	5.923		
9	rs9919066	106766642	q31.1	T	0.1447	0.0278	C	6.312	1.20E-02	5.923		
13	rs884318	60219685	q21.2	C	0.1184	0.0143	T	6.193	1.28E-02	9.269		
4	rs976057	29802859	p15.1	G	0.1711	0.0429	A	6.137	1.32E-02	4.608		
12	rs10859997	94909490	q23.1	T	0.2105	0.0694	C	6.044	1.40E-02	3.573	HAL	intronic
5	rs10940065	65889320	q12.3	G	0.2105	0.0694	A	6.044	1.40E-02	3.573		
13	rs1478654	54079192	q14.3	A	0.2105	0.0694	G	6.044	1.40E-02	3.573		
3	rs17040736	4490191	p26.1	T	0.2105	0.0694	G	6.044	1.40E-02	3.573		
1	rs1766807	119208283	p12	G	0.2105	0.0694	A	6.044	1.40E-02	3.573		
7	rs2529015	20293111	p21.1	A	0.2105	0.0694	G	6.044	1.40E-02	3.573		
12	rs2576956	102796319	q23.3	A	0.2105	0.0694	G	6.044	1.40E-02	3.573	GNN	intronic
10	rs2767699	7447340	p14	C	0.2105	0.0694	T	6.044	1.40E-02	3.573	SFMBT2	intronic
5	rs340697	8475435	p15.31	T	0.2105	0.0694	C	6.044	1.40E-02	3.573	LOC729506	non-coding intronic
4	rs6833118	6884695	p16.1	T	0.2105	0.0694	C	6.044	1.40E-02	3.573	KIAA0232	intronic
13	rs9527234	54080824	q14.3	C	0.2105	0.0694	T	6.044	1.40E-02	3.573		
13	rs9536797	54140619	q14.3	C	0.2105	0.0694	A	6.044	1.40E-02	3.573		
12	rs9669708	102786200	q23.3	T	0.2105	0.0694	G	6.044	1.40E-02	3.573	GNN	5upstream/intronic
11	rs4638337	111777399	q23.1	C	0.2500	0.0972	A	5.959	1.46E-02	3.095		
12	rs701039	123570315	q24.31	C	0.2500	0.0972	T	5.959	1.46E-02	3.095	NCOR2	intronic/5upstream

11	rs7932795	111786436	q23.1	G	0.2500	0.0972	A	5.959	1.46E-02	3.095		
14	rs6575129	89913545	q32.11	T	0.2297	0.0833	G	5.893	1.52E-02	3.281		
8	rs10505201	114295819	q23.3	A	0.1622	0.0417	G	5.748	1.65E-02	4.452	CSMD3	intronic
5	rs13158904	65572109	q12.3	A	0.1842	0.0556	G	5.729	1.67E-02	3.839		
3	rs17040776	4505278	p26.1	C	0.1842	0.0556	A	5.729	1.67E-02	3.839	ITPR1_AS1	3downstream
7	rs2348558	7808086	p21.3	C	0.1842	0.0556	T	5.729	1.67E-02	3.839	LOC729852	intronic
11	rs511514	121498790	q24.1	G	0.1842	0.0556	A	5.729	1.67E-02	3.839	MIR100HG	non-coding intronic
12	rs636299	51238751	q13.13	T	0.1842	0.0556	G	5.729	1.67E-02	3.839		
10	rs604772	13096593	p13	C	0.2105	0.0714	T	5.725	1.67E-02	3.467	CCDC3	non-coding intronic
22	rs715001	42075141	q13.2	A	0.2105	0.0714	C	5.725	1.67E-02	3.467		
5	rs16888617	21726343	p14.3	A	0.1081	0.0139	C	5.601	1.80E-02	8.606	BC038535	non-coding intronic
11	rs7945210	26673637	p14.2	C	0.1389	0.0286	T	5.583	1.81E-02	5.484	SLC5A12	intronic
3	rs11917422	142505177	q23	C	0.2237	0.0833	T	5.549	1.85E-02	3.169		
19	rs1476592	3630740	p13.3	G	0.2237	0.0833	A	5.549	1.85E-02	3.169	PIP5K1C	intronic
2	rs17032466	66768195	p14	G	0.2237	0.0833	A	5.549	1.85E-02	3.169	AC007392.3	non-coding intronic
14	rs17833053	58251677	q23.1	A	0.2237	0.0833	G	5.549	1.85E-02	3.169		
1	rs10159449	107577827	p13.3	G	0.1579	0.0417	A	5.484	1.92E-02	4.312	NTNG1	intronic
12	rs11609754	124798180	q24.32	C	0.1579	0.0417	T	5.484	1.92E-02	4.312		
15	rs11638359	96460308	q26.3	G	0.1579	0.0417	A	5.484	1.92E-02	4.312		
16	rs11646232	53648412	q12.2	T	0.1579	0.0417	G	5.484	1.92E-02	4.312		
22	rs13057831	41203476	q13.2	C	0.1579	0.0417	T	5.484	1.92E-02	4.312		
10	rs7903957	28502025	p12.1	A	0.1579	0.0417	G	5.484	1.92E-02	4.312	MPP7	intronic
6	rs9342947	73222052	q13	C	0.1579	0.0417	T	5.484	1.92E-02	4.312		
13	rs9536319	52394664	q14.3	T	0.1579	0.0417	C	5.484	1.92E-02	4.312		
3	rs12637065	145748530	q24	T	0.2027	0.0694	G	5.482	1.92E-02	3.407		
14	rs3784039	73559488	q24.3	T	0.2027	0.0694	C	5.482	1.92E-02	3.407	CCDC176	coding
3	rs10490826	145745200	q24	C	0.1053	0.0139	T	5.405	2.01E-02	8.353		
5	rs10514254	82669707	q14.2	A	0.1053	0.0139	G	5.405	2.01E-02	8.353	XRCC4	intronic
13	rs12429374	69292832	q21.33	G	0.1053	0.0139	T	5.405	2.01E-02	8.353	KLHL1	intronic
12	rs12819069	31281817	p11.21	C	0.1053	0.0139	T	5.405	2.01E-02	8.353		
1	rs17301783	60847990	p32.1	T	0.1053	0.0139	C	5.405	2.01E-02	8.353		
5	rs17439022	32525961	p13.3	A	0.1053	0.0139	C	5.405	2.01E-02	8.353		
2	rs17481321	47078431	p21	A	0.1053	0.0139	C	5.405	2.01E-02	8.353	TTC7A	intronic
8	rs17504762	38589163	p11.22	G	0.1053	0.0139	A	5.405	2.01E-02	8.353		
13	rs1890046	69262301	q21.33	A	0.1053	0.0139	G	5.405	2.01E-02	8.353	KLHL1	intronic
12	rs7139314	109408254	q24.11	A	0.1053	0.0139	G	5.405	2.01E-02	8.353	FAM216A	intronic
8	rs960609	120294053	q24.12	A	0.1053	0.0139	G	5.405	2.01E-02	8.353	MAL2	intronic
7	rs17704630	95076579	q21.3	A	0.1351	0.0286	G	5.348	2.08E-02	5.312		
8	rs2688266	3778770	p23.2	G	0.1351	0.0286	A	5.348	2.08E-02	5.312	CSMD1	intronic
6	rs1040939	44621319	p21.1	T	0.1316	0.0278	C	5.347	2.08E-02	5.303		
10	rs12569831	33807553	p11.22	A	0.1316	0.0278	G	5.347	2.08E-02	5.303		
5	rs1390663	21725594	p14.3	C	0.1316	0.0278	A	5.347	2.08E-02	5.303	BC038535	non-coding intronic
11	rs17309356	26664973	p14.2	A	0.1316	0.0278	G	5.347	2.08E-02	5.303	SLC5A12	non-coding intronic
1	rs17680745	110825891	p13.3	T	0.1316	0.0278	C	5.347	2.08E-02	5.303	CYMP	non-coding intronic
10	rs1925260	119570720	q26.11	A	0.1316	0.0278	G	5.347	2.08E-02	5.303		
10	rs2997203	28485509	p12.1	T	0.1316	0.0278	C	5.347	2.08E-02	5.303	MPP7	intronic
2	rs3820700	73570318	p13.1	T	0.1316	0.0278	C	5.347	2.08E-02	5.303	ALMS1	coding
8	rs417994	17735510	p22	C	0.1316	0.0278	T	5.347	2.08E-02	5.303		
16	rs7196605	72145309	q22.3	C	0.1316	0.0278	T	5.347	2.08E-02	5.303		
12	rs10850906	116743249	q24.23	T	0.1053	0.0143	C	5.214	2.24E-02	8.118	KSR2	intronic
15	rs5006363	20398953	q11.2	A	0.1081	0.0147	C	5.208	2.25E-02	8.121	TUBGCP5	intronic/5UPSTREAM
4	rs187196	21113711	p15.2	T	0.1111	0.0152	C	5.201	2.26E-02	8.125	KCNIP4	intronic
20	rs6133438	7519155	p12.3	G	0.2027	0.0714	A	5.183	2.28E-02	3.305		
14	rs10873266	73755366	q24.3	T	0.1974	0.0694	C	5.177	2.29E-02	3.295		
7	rs11762933	33888281	p14.3	A	0.1974	0.0694	G	5.177	2.29E-02	3.295	AK025321	5upstream
11	rs11827471	119627604	q23.3	G	0.1974	0.0694	A	5.177	2.29E-02	3.295	POU2F3	intronic
5	rs12520189	143546081	q31.3	A	0.1974	0.0694	G	5.177	2.29E-02	3.295	KCTD16	intronic
9	rs1538581	71081576	q21.11	T	0.1974	0.0694	C	5.177	2.29E-02	3.295		
3	rs1574130	126255281	q21.2	A	0.1974	0.0694	G	5.177	2.29E-02	3.295	HEG1	intronic
17	rs16959281	9918192	p13.1	T	0.1974	0.0694	C	5.177	2.29E-02	3.295	GAS7	intronic
5	rs17101605	143516897	q31.3	A	0.1974	0.0694	C	5.177	2.29E-02	3.295	YIPF5	3downstream
14	rs2300193	73637489	q24.3	G	0.1974	0.0694	A	5.177	2.29E-02	3.295	LIN52	intronic
1	rs4276859	24288720	p36.11	T	0.1974	0.0694	C	5.177	2.29E-02	3.295	MYOM3	coding
5	rs7704225	143508167	q31.3	A	0.1974	0.0694	G	5.177	2.29E-02	3.295		
7	rs7787218	33904913	p14.3	C	0.1974	0.0694	T	5.177	2.29E-02	3.295		
14	rs848049	36388208	q13.3	T	0.1974	0.0694	C	5.177	2.29E-02	3.295	SLC25A21	intronic
11	rs10500905	21333247	p15.1	G	0.1316	0.0286	A	5.125	2.36E-02	5.152	NELL1	intronic
4	rs1355161	126487591	q28.1	G	0.1316	0.0286	A	5.125	2.36E-02	5.152	FAT4	intronic
2	rs1560251	227362192	q36.3	T	0.1757	0.0556	G	5.118	2.37E-02	3.623	IRS1	intronic
12	rs17730330	91522645	q22	G	0.1757	0.0556	A	5.118	2.37E-02	3.623		
7	rs7789161	53588859	p12.1	T	0.1757	0.0556	C	5.118	2.37E-02	3.623		
13	rs646063	29440630	q12.3	C	0.1286	0.0278	T	5.046	2.47E-02	5.164		
1	rs2594683	104755888	p21.1	T	0.1053	0.0147	C	5.023	2.50E-02	7.882		

13	rs2147151	107957928	q33.3	T	0.1000	0.0139	G	4.95	2.61E-02	7.889		
11	rs578193	56240622	q12.1	T	0.1000	0.0139	C	4.95	2.61E-02	7.889	OR8U8	intronic
2	rs6758645	76424948	p12	C	0.1316	0.0294	T	4.904	2.68E-02	5		
8	rs12676965	37800584	p11.23	C	0.1974	0.0714	T	4.889	2.70E-02	3.197	GPR124	intronic
1	rs7528206	18679704	p36.13	C	0.1974	0.0714	T	4.889	2.70E-02	3.197	KLHDC7A	5upstream
23	rs4829124	32673682	p21.1	A	0.2045	0.0566	G	4.854	2.76E-02	4.286	DMD	intronic
4	rs10010385	82583986	q21.21	C	0.1711	0.0556	T	4.851	2.76E-02	3.508	RASGEF1B	intronic
9	rs1032474	14765853	p22.3	A	0.1711	0.0556	G	4.851	2.76E-02	3.508	FREM1	coding/5upstream/intronic
12	rs10492340	91501595	q22	C	0.1711	0.0556	T	4.851	2.76E-02	3.508		
5	rs1422687	164787735	q34	T	0.1711	0.0556	C	4.851	2.76E-02	3.508		
7	rs1880740	48244761	p12.3	A	0.1711	0.0556	G	4.851	2.76E-02	3.508	ABCA13	intronic
20	rs2232081	6008395	p12.3	A	0.1711	0.0556	G	4.851	2.76E-02	3.508	FERMT1	intronic
15	rs275178	37235472	q14	T	0.1711	0.0556	C	4.851	2.76E-02	3.508		
9	rs4415414	14768244	p22.3	T	0.1711	0.0556	G	4.851	2.76E-02	3.508	FREM1	intronic
2	rs4468781	19946372	p24.1	C	0.1711	0.0556	T	4.851	2.76E-02	3.508	LINC00954	non-coding intronic
4	rs6448609	28518653	p15.1	T	0.1711	0.0556	G	4.851	2.76E-02	3.508		
7	rs705358	90515611	q21.13	T	0.1711	0.0556	C	4.851	2.76E-02	3.508	CDK14	intronic
13	rs7336718	89280044	q31.3	G	0.1711	0.0556	A	4.851	2.76E-02	3.508		
2	rs7579437	20591570	p24.1	A	0.1711	0.0556	G	4.851	2.76E-02	3.508		
6	rs9465946	21117867	p22.3	G	0.1711	0.0556	A	4.851	2.76E-02	3.508	CDKAL1	intronic
13	rs9522755	89275755	q31.3	T	0.1711	0.0556	C	4.851	2.76E-02	3.508		
13	rs9522767	89301151	q31.3	C	0.1711	0.0556	T	4.851	2.76E-02	3.508		
13	rs9538881	60180632	q21.2	C	0.1711	0.0556	T	4.851	2.76E-02	3.508		
8	rs2688304	3781284	p23.2	G	0.2143	0.0833	T	4.83	2.80E-02	3	CSMD1	intronic
11	rs7108738	15666660	p15.2	G	0.2143	0.0833	T	4.83	2.80E-02	3		
8	rs11987577	54049972	q11.23	T	0.1486	0.0417	C	4.818	2.82E-02	4.016		
7	rs12234501	8738675	p21.3	T	0.1486	0.0417	C	4.818	2.82E-02	4.016	NXPH1	intronic
12	rs2277383	50600655	q13.13	G	0.1486	0.0417	T	4.818	2.82E-02	4.016	ACVRL1	intronic
23	rs195268	79125114	q21.1	G	0.1333	0.0189	A	4.808	2.83E-02	8		
23	rs929228	8781555	p22.31	C	0.1333	0.0189	T	4.808	2.83E-02	8		
23	rs2795289	136667419	q26.3	T	0.2000	0.0566	C	4.657	3.09E-02	4.167		
13	rs3106557	60224141	q21.2	A	0.1892	0.0694	G	4.622	3.16E-02	3.127		
5	rs3805608	127508897	q23.3	A	0.1892	0.0694	G	4.622	3.16E-02	3.127	SLC12A2	intronic
17	rs7224163	109700	p13.3	G	0.1216	0.0278	A	4.613	3.17E-02	4.846	RPH3AL	intronic
12	rs9783406	94565342	q22	T	0.1216	0.0278	C	4.613	3.17E-02	4.846	PGAM1P5	5upstream
9	rs2809841	82979213	q21.31	T	0.1711	0.0571	G	4.596	3.21E-02	3.405		
17	rs4290532	68530900	q25.1	G	0.1711	0.0571	A	4.596	3.21E-02	3.405	SLC39A11	intronic
1	rs946819	176261095	q25.2	A	0.1711	0.0571	G	4.596	3.21E-02	3.405	SEC16B	intronic
4	rs13150558	61870241	q13.1	A	0.0972	0.0143	G	4.592	3.21E-02	7.431	LPHN3	intronic
3	rs6766872	32720800	p22.3	G	0.1757	0.0588	T	4.591	3.21E-02	3.41	CNOT10	intronic/5UPSTREAM
3	rs11715062	54534143	p14.3	T	0.0946	0.0139	C	4.589	3.22E-02	7.418	CACNA2D3	intronic
2	rs17013391	33652246	p22.3	G	0.0946	0.0139	A	4.589	3.22E-02	7.418		
4	rs7661156	125574490	q28.1	G	0.0946	0.0139	A	4.589	3.22E-02	7.418		
8	rs10505571	132586517	q24.22	T	0.1447	0.0417	C	4.586	3.22E-02	3.892		
5	rs10512747	40877498	p13.1	T	0.1447	0.0417	C	4.586	3.22E-02	3.892	CARD6	coding
4	rs10517601	155853294	q32.1	G	0.1447	0.0417	A	4.586	3.22E-02	3.892	DQ266889	non-coding intronic
12	rs10770674	20646893	p12.2	C	0.1447	0.0417	T	4.586	3.22E-02	3.892	PDE3A	intronic
7	rs1108845	151153648	q36.1	A	0.1447	0.0417	G	4.586	3.22E-02	3.892	PRKAG2	intronic
11	rs11226195	103511271	q22.3	T	0.1447	0.0417	C	4.586	3.22E-02	3.892	PDGFD	intronic
11	rs12277818	103507652	q22.3	T	0.1447	0.0417	C	4.586	3.22E-02	3.892	PDGFD	intronic
2	rs13414599	179928297	q31.2	A	0.1447	0.0417	G	4.586	3.22E-02	3.892		
18	rs1395813	71869875	q23	T	0.1447	0.0417	C	4.586	3.22E-02	3.892		
4	rs16875239	23787325	p15.2	A	0.1447	0.0417	G	4.586	3.22E-02	3.892	PPARGC1A	non-coding intronic
8	rs17605272	114181979	q23.3	T	0.1447	0.0417	C	4.586	3.22E-02	3.892	CSMD3	intronic
10	rs2254849	70985384	q22.1	T	0.1447	0.0417	C	4.586	3.22E-02	3.892		
21	rs2269139	42977018	q22.3	T	0.1447	0.0417	C	4.586	3.22E-02	3.892	PDE9A	intronic
15	rs4777161	67648593	q23	A	0.1447	0.0417	G	4.586	3.22E-02	3.892	LOC145837	non-coding intronic
15	rs6494808	67655995	q23	T	0.1447	0.0417	C	4.586	3.22E-02	3.892		
8	rs6996697	114343451	q23.3	G	0.1447	0.0417	A	4.586	3.22E-02	3.892	CSMD3	intronic
8	rs736726	132587879	q24.22	A	0.1447	0.0417	G	4.586	3.22E-02	3.892		
12	rs7956157	81836159	q21.31	C	0.1447	0.0417	A	4.586	3.22E-02	3.892	TMTC2	intronic
14	rs8021398	75916679	q24.3	T	0.1447	0.0417	C	4.586	3.22E-02	3.892	ESRRB	intronic
11	rs924561	103504269	q22.3	G	0.1447	0.0417	A	4.586	3.22E-02	3.892	PDGFD	intronic
6	rs9503907	4002541	p25.2	G	0.1447	0.0417	T	4.586	3.22E-02	3.892	PRPF4B	intronic
13	rs9522712	89239579	q31.3	T	0.1447	0.0417	C	4.586	3.22E-02	3.892		
2	rs7559158	33690426	p22.3	C	0.1667	0.0556	A	4.5	3.39E-02	3.4		
5	rs10058813	27423142	p14.1	T	0.0921	0.0139	G	4.424	3.54E-02	7.203		
2	rs10496905	142352930	q22.2	G	0.0921	0.0139	A	4.424	3.54E-02	7.203	LRP1B	intronic
10	rs10732804	112785011	q25.2	A	0.0921	0.0139	G	4.424	3.54E-02	7.203		
11	rs1117170	96709050	q22.1	T	0.0921	0.0139	G	4.424	3.54E-02	7.203		
17	rs11651058	8828830	p13.1	G	0.0921	0.0139	A	4.424	3.54E-02	7.203		
2	rs11693228	17756958	p24.2	A	0.0921	0.0139	G	4.424	3.54E-02	7.203	SMC6	intronic
1	rs11801137	104699371	p21.1	G	0.0921	0.0139	A	4.424	3.54E-02	7.203		

2	rs12104712	82498459	p12	T	0.0921	0.0139	G	4.424	3.54E-02	7.203		
1	rs12123337	19734998	p36.13	C	0.0921	0.0139	T	4.424	3.54E-02	7.203		
9	rs12345817	29823969	p21.1	G	0.0921	0.0139	T	4.424	3.54E-02	7.203		
4	rs12501459	46918110	p12	G	0.0921	0.0139	A	4.424	3.54E-02	7.203	GABRB1	intronic
1	rs12564848	172542992	q25.1	C	0.0921	0.0139	T	4.424	3.54E-02	7.203	RABGAP1L	intronic
5	rs13177718	108141243	q21.3	T	0.0921	0.0139	C	4.424	3.54E-02	7.203	FER	non-coding intronic
6	rs1537590	150224322	q25.1	C	0.0921	0.0139	A	4.424	3.54E-02	7.203	LRP11	intronic
4	rs1567111	178434231	q34.3	G	0.0921	0.0139	A	4.424	3.54E-02	7.203		
10	rs1621751	118547458	q25.3	G	0.0921	0.0139	A	4.424	3.54E-02	7.203	HSPA12A	intronic
1	rs16847402	172981674	q25.1	C	0.0921	0.0139	T	4.424	3.54E-02	7.203	RABGAP1L	intronic
1	rs16847563	173075477	q25.1	C	0.0921	0.0139	T	4.424	3.54E-02	7.203	RABGAP1L	intronic
5	rs16873241	71256017	q13.2	G	0.0921	0.0139	A	4.424	3.54E-02	7.203		
5	rs16896657	27359503	p14.1	C	0.0921	0.0139	T	4.424	3.54E-02	7.203		
11	rs16924357	7262671	p15.4	C	0.0921	0.0139	A	4.424	3.54E-02	7.203	SYT9	intronic
16	rs16966503	62328476	q21	C	0.0921	0.0139	T	4.424	3.54E-02	7.203		
12	rs17682012	109578837	q24.11	T	0.0921	0.0139	C	4.424	3.54E-02	7.203	HVCN1	intronic
1	rs1890881	172443546	q25.1	C	0.0921	0.0139	T	4.424	3.54E-02	7.203	RABGAP1L	intronic
9	rs1927880	82363174	q21.31	T	0.0921	0.0139	C	4.424	3.54E-02	7.203		
14	rs1957283	91837433	q32.12	T	0.0921	0.0139	C	4.424	3.54E-02	7.203		
1	rs2072758	172792540	q25.1	A	0.0921	0.0139	G	4.424	3.54E-02	7.203	RABGAP1L	intronic
17	rs210834	29802339	q12	G	0.0921	0.0139	A	4.424	3.54E-02	7.203		
1	rs2430080	186050561	q31.1	A	0.0921	0.0139	C	4.424	3.54E-02	7.203		
1	rs2430084	186047873	q31.1	C	0.0921	0.0139	T	4.424	3.54E-02	7.203		
12	rs2471495	80715574	q21.31	A	0.0921	0.0139	G	4.424	3.54E-02	7.203		
1	rs2506986	47878679	p33	C	0.0921	0.0139	A	4.424	3.54E-02	7.203		
9	rs2891333	29781089	p21.1	G	0.0921	0.0139	T	4.424	3.54E-02	7.203		
18	rs359731	71854650	q23	C	0.0921	0.0139	T	4.424	3.54E-02	7.203		
11	rs4313593	67194771	q13.2	T	0.0921	0.0139	C	4.424	3.54E-02	7.203	ALDH3B2	intronic
1	rs4651180	182421203	q25.3	A	0.0921	0.0139	G	4.424	3.54E-02	7.203		
8	rs4873647	53551855	q11.23	G	0.0921	0.0139	A	4.424	3.54E-02	7.203		
8	rs4873666	53659338	q11.23	A	0.0921	0.0139	G	4.424	3.54E-02	7.203		
11	rs7107048	11974796	p15.3	T	0.0921	0.0139	C	4.424	3.54E-02	7.203	DKK3	intronic
5	rs717163	31893190	p13.3	C	0.0921	0.0139	A	4.424	3.54E-02	7.203	PDZD2	intronic
12	rs7311376	109089942	q24.11	T	0.0921	0.0139	C	4.424	3.54E-02	7.203	IFT81	intronic
2	rs7596891	45899087	p21	T	0.0921	0.0139	C	4.424	3.54E-02	7.203	PRKCE	intronic
12	rs7966591	109128428	q24.11	A	0.0921	0.0139	G	4.424	3.54E-02	7.203	IFT81	intronic
14	rs8018748	42191841	q21.1	T	0.0921	0.0139	G	4.424	3.54E-02	7.203		
12	rs916973	111931486	q24.13	C	0.0921	0.0139	T	4.424	3.54E-02	7.203	OAS2	3utr/intronic
3	rs9882385	156035277	q25.2	C	0.0921	0.0139	A	4.424	3.54E-02	7.203		
5	rs10515395	108272703	q21.3	C	0.1184	0.0278	T	4.415	3.56E-02	4.701	FER	non-coding intronic
12	rs11108364	94905106	q23.1	G	0.1184	0.0278	A	4.415	3.56E-02	4.701	HAL	coding
12	rs11169945	50561776	q13.13	C	0.1184	0.0278	T	4.415	3.56E-02	4.701		
1	rs11577033	23910818	p36.11	C	0.1184	0.0278	T	4.415	3.56E-02	4.701		
1	rs11587587	233625329	q42.3	T	0.1184	0.0278	C	4.415	3.56E-02	4.701	TBCE	non-coding intronic
1	rs12135902	153022728	q21.3	A	0.1184	0.0278	G	4.415	3.56E-02	4.701	KCNN3	intronic
5	rs12186507	20994575	p14.3	A	0.1184	0.0278	C	4.415	3.56E-02	4.701		
14	rs12433929	91533374	q32.12	G	0.1184	0.0278	A	4.415	3.56E-02	4.701	TRIP11	intronic
1	rs12745648	233605643	q42.3	G	0.1184	0.0278	A	4.415	3.56E-02	4.701	TBCE	intronic
1	rs12757197	233657182	q42.3	A	0.1184	0.0278	G	4.415	3.56E-02	4.701	TBCE	intronic
15	rs12902244	99789578	q26.3	C	0.1184	0.0278	T	4.415	3.56E-02	4.701	PCSK6	intronic
18	rs1431381	6802509	p11.31	A	0.1184	0.0278	C	4.415	3.56E-02	4.701	ARHGAP28	intronic
2	rs16862382	15198595	p24.3	G	0.1184	0.0278	A	4.415	3.56E-02	4.701		
6	rs16900468	158181346	q25.3	A	0.1184	0.0278	G	4.415	3.56E-02	4.701	SNX9	intronic
11	rs16906047	20306246	p15.1	T	0.1184	0.0278	G	4.415	3.56E-02	4.701		
9	rs16928802	128031443	q33.3	T	0.1184	0.0278	C	4.415	3.56E-02	4.701		
13	rs17532490	40581061	q14.11	A	0.1184	0.0278	G	4.415	3.56E-02	4.701		
6	rs17712040	153452788	q25.2	C	0.1184	0.0278	T	4.415	3.56E-02	4.701	RGS17	intronic
14	rs17807815	91625808	q32.12	C	0.1184	0.0278	T	4.415	3.56E-02	4.701	ATXN3	intronic
8	rs2410584	18646634	p22	G	0.1184	0.0278	A	4.415	3.56E-02	4.701	PSD3	intronic
7	rs288758	155319096	q36.3	A	0.1184	0.0278	G	4.415	3.56E-02	4.701		
13	rs3119878	60282057	q21.2	A	0.1184	0.0278	G	4.415	3.56E-02	4.701		
17	rs3743990	71102780	q25.1	T	0.1184	0.0278	C	4.415	3.56E-02	4.701	MYO15B	intronic
21	rs3819895	42970809	q22.3	C	0.1184	0.0278	T	4.415	3.56E-02	4.701	PDE9A	intronic
15	rs4442780	71006306	q24.1	C	0.1184	0.0278	T	4.415	3.56E-02	4.701		
13	rs561605	29461154	q12.3	G	0.1184	0.0278	A	4.415	3.56E-02	4.701		
20	rs6135817	1663448	p13	T	0.1184	0.0278	C	4.415	3.56E-02	4.701		
10	rs684903	7438806	p14	C	0.1184	0.0278	T	4.415	3.56E-02	4.701	SFMBT2	intronic
9	rs7042583	137244189	q34.3	T	0.1184	0.0278	C	4.415	3.56E-02	4.701		
8	rs799887	117322902	q23.3	G	0.1184	0.0278	A	4.415	3.56E-02	4.701	LINC00536	non-coding intronic
22	rs8139020	18008737	q11.21	C	0.1184	0.0278	A	4.415	3.56E-02	4.701		
17	rs9915405	65867276	q24.3	G	0.1184	0.0278	A	4.415	3.56E-02	4.701		
2	rs8179874	1027731	p25.3	G	0.1429	0.0417	A	4.37	3.66E-02	3.833	SNTG2	intronic
19	rs16027	13258560	p13.2	A	0.1447	0.0429	G	4.363	3.67E-02	3.779	CACNA1A	coding

5	rs10059139	64258718	q12.3	C	0.1842	0.0694	T	4.352	3.70E-02	3.026	CWC27	intronic
3	rs10510230	2257130	p26.3	C	0.1842	0.0694	A	4.352	3.70E-02	3.026	CNTN4	intronic
1	rs10911031	180440334	q25.3	A	0.1842	0.0694	G	4.352	3.70E-02	3.026	GS1_122H1.2	non-coding intronic
9	rs10970061	31020836	p21.1	A	0.1842	0.0694	G	4.352	3.70E-02	3.026		
8	rs12155549	119255578	q24.11	C	0.1842	0.0694	T	4.352	3.70E-02	3.026		
15	rs12440772	68091211	q23	A	0.1842	0.0694	G	4.352	3.70E-02	3.026		
4	rs1349683	124618510	q28.1	G	0.1842	0.0694	A	4.352	3.70E-02	3.026		
5	rs16901644	31902960	p13.3	A	0.1842	0.0694	G	4.352	3.70E-02	3.026	PDZD2	intronic
9	rs1765372	33689373	p13.3	A	0.1842	0.0694	C	4.352	3.70E-02	3.026		
17	rs17823987	4511439	p13.2	C	0.1842	0.0694	A	4.352	3.70E-02	3.026		
9	rs2151414	70562820	q21.11	G	0.1842	0.0694	A	4.352	3.70E-02	3.026	PIP5K1B	non-coding intronic
12	rs2293094	50602870	q13.13	G	0.1842	0.0694	A	4.352	3.70E-02	3.026	ACVRL1	3utr
9	rs2416864	124293834	q33.2	A	0.1842	0.0694	G	4.352	3.70E-02	3.026	OR1J2	intronic
6	rs3846814	67116910	q12	T	0.1842	0.0694	C	4.352	3.70E-02	3.026		
4	rs4561891	165388371	q32.3	C	0.1842	0.0694	A	4.352	3.70E-02	3.026	MARCH1	intronic
4	rs4689665	7294301	p16.1	T	0.1842	0.0694	C	4.352	3.70E-02	3.026	SORCS2	intronic
16	rs4889428	80482495	q23.3	T	0.1842	0.0694	C	4.352	3.70E-02	3.026	PLCG2	intronic
20	rs6045924	199494	p13	G	0.1842	0.0694	T	4.352	3.70E-02	3.026	C20orf96	3downstream
6	rs6927917	73214183	q13	A	0.1842	0.0694	G	4.352	3.70E-02	3.026		
10	rs7076334	25497906	p12.1	A	0.1842	0.0694	G	4.352	3.70E-02	3.026	GPR158-AS1	intronic
10	rs7081721	4586561	p15.1	A	0.1842	0.0694	G	4.352	3.70E-02	3.026		
14	rs7159883	58347513	q23.1	A	0.1842	0.0694	C	4.352	3.70E-02	3.026		
17	rs7211876	75925955	q25.3	T	0.1842	0.0694	C	4.352	3.70E-02	3.026	RNF213	intronic/3downstream
14	rs735148	93392382	q32.12	T	0.1842	0.0694	C	4.352	3.70E-02	3.026		
7	rs7455906	79996797	q21.11	T	0.1842	0.0694	C	4.352	3.70E-02	3.026	CD36	intronic
12	rs7956222	99730699	q23.1	C	0.1842	0.0694	T	4.352	3.70E-02	3.026	ANO4	intronic
9	rs855438	33693234	p13.3	C	0.1842	0.0694	T	4.352	3.70E-02	3.026		
3	rs931317	59903891	p14.2	G	0.1842	0.0694	A	4.352	3.70E-02	3.026	FHIT	intronic
3	rs10510621	29491643	p24.1	A	0.1622	0.0556	G	4.25	3.92E-02	3.29	RBMS3	intronic
11	rs1942849	128037597	q24.3	C	0.1622	0.0556	T	4.25	3.92E-02	3.29		
2	rs4467294	102724201	q12.1	G	0.1622	0.0556	T	4.25	3.92E-02	3.29	TMEM182	intronic
10	rs7091469	52742174	q21.1	T	0.1622	0.0556	C	4.25	3.92E-02	3.29	PRKG1	intronic
23	rs2106831	9137044	p22.31	T	0.2500	0.0943	C	4.229	3.97E-02	3.2		
15	rs3812920	20419703	q11.2	T	0.1184	0.0286	C	4.223	3.99E-02	4.567	TUBGCP5	intronic
18	rs9807656	40600954	q12.3	C	0.1184	0.0286	T	4.223	3.99E-02	4.567	SETBP1	intronic
12	rs12371626	65102768	q14.3	T	0.1389	0.0417	C	4.143	4.18E-02	3.71	GRIP1	intronic
14	rs1168414	37817897	q21.1	A	0.1143	0.0278	G	4.057	4.40E-02	4.516		
3	rs1546038	79518922	p12.3	T	0.1143	0.0278	C	4.057	4.40E-02	4.516	ROBO1	intronic
8	rs2440445	6330927	p23.1	G	0.1143	0.0278	A	4.057	4.40E-02	4.516	MCPH1	intronic
16	rs8063568	17646047	p12.3	A	0.1143	0.0278	C	4.057	4.40E-02	4.516		
4	rs2178095	185846784	q35.1	A	0.1184	0.0294	G	4.03	4.47E-02	4.433	CCDC111	intronic
8	rs11136072	29400345	p12	G	0.1579	0.0556	A	4.016	4.51E-02	3.188		
6	rs11153496	115112039	q22.1	A	0.1579	0.0556	G	4.016	4.51E-02	3.188		
10	rs11592670	114074341	q25.2	A	0.1579	0.0556	C	4.016	4.51E-02	3.188	GUCY2GP	non-coding intronic
10	rs11815261	33778050	p11.22	G	0.1579	0.0556	T	4.016	4.51E-02	3.188		
15	rs11853146	48195316	q21.2	T	0.1579	0.0556	C	4.016	4.51E-02	3.188	ATP8B4	intronic
3	rs11915214	54616060	p14.3	A	0.1579	0.0556	G	4.016	4.51E-02	3.188	CACNA2D3	intronic
5	rs11958845	170208539	q35.1	A	0.1579	0.0556	G	4.016	4.51E-02	3.188		
1	rs12128858	34496515	p34.3	C	0.1579	0.0556	T	4.016	4.51E-02	3.188		
12	rs12318442	20637274	p12.2	T	0.1579	0.0556	C	4.016	4.51E-02	3.188	PDE3A	intronic
18	rs12606713	48433842	q21.2	G	0.1579	0.0556	A	4.016	4.51E-02	3.188	DCC	intronic
4	rs12643033	170000887	q32.3	G	0.1579	0.0556	T	4.016	4.51E-02	3.188	PALLD	intronic
5	rs12659543	71173487	q13.2	A	0.1579	0.0556	G	4.016	4.51E-02	3.188		
10	rs12776250	11423030	p14	C	0.1579	0.0556	T	4.016	4.51E-02	3.188		
5	rs13161696	66046930	q12.3	A	0.1579	0.0556	C	4.016	4.51E-02	3.188	MAST4	intronic
4	rs1518000	18398373	p15.31	G	0.1579	0.0556	T	4.016	4.51E-02	3.188		
15	rs16975846	94280228	q26.2	C	0.1579	0.0556	T	4.016	4.51E-02	3.188		
4	rs17005729	141450708	q31.1	T	0.1579	0.0556	C	4.016	4.51E-02	3.188	SCOC	intronic
3	rs17049174	8484497	p26.1	A	0.1579	0.0556	G	4.016	4.51E-02	3.188	LMCD1-AS1	non-coding intronic
12	rs17104186	66320139	q15	C	0.1579	0.0556	T	4.016	4.51E-02	3.188		
7	rs17141124	19320105	p21.1	T	0.1579	0.0556	G	4.016	4.51E-02	3.188		
9	rs17583891	137436405	q34.3	A	0.1579	0.0556	G	4.016	4.51E-02	3.188		
5	rs17794637	11358217	p15.2	T	0.1579	0.0556	C	4.016	4.51E-02	3.188	CTNND2	intronic
14	rs17799268	89858687	q32.11	A	0.1579	0.0556	C	4.016	4.51E-02	3.188	NRDE2	intronic
5	rs2162804	63660182	q12.3	A	0.1579	0.0556	G	4.016	4.51E-02	3.188	RNF180	intronic
5	rs2216588	170214294	q35.1	G	0.1579	0.0556	T	4.016	4.51E-02	3.188		
7	rs2390081	19326307	p21.1	G	0.1579	0.0556	A	4.016	4.51E-02	3.188		
6	rs2397008	50616436	p12.3	C	0.1579	0.0556	A	4.016	4.51E-02	3.188		
9	rs2809845	82966078	q21.31	A	0.1579	0.0556	C	4.016	4.51E-02	3.188		
10	rs2812562	70980738	q22.1	C	0.1579	0.0556	T	4.016	4.51E-02	3.188		
3	rs2863836	167943645	q26.1	G	0.1579	0.0556	A	4.016	4.51E-02	3.188		
4	rs4048413	174253216	q34.1	A	0.1579	0.0556	G	4.016	4.51E-02	3.188		
6	rs4074101	56670411	p12.1	T	0.1579	0.0556	C	4.016	4.51E-02	3.188	DST	intronic

15	rs4238465	91527661	q26.1	A	0.1579	0.0556	G	4.016	4.51E-02	3.188		
4	rs4697300	22058901	p15.2	G	0.1579	0.0556	T	4.016	4.51E-02	3.188	GPR125	intronic
7	rs4725033	7820533	p21.3	C	0.1579	0.0556	T	4.016	4.51E-02	3.188	LOC729852	intronic
14	rs4906523	43864397	q21.2	G	0.1579	0.0556	A	4.016	4.51E-02	3.188		
22	rs5750283	35424439	q12.3	G	0.1579	0.0556	A	4.016	4.51E-02	3.188	CACNG2	intronic
8	rs6474057	57372209	q12.1	C	0.1579	0.0556	T	4.016	4.51E-02	3.188		
15	rs6493395	48131276	q21.2	T	0.1579	0.0556	C	4.016	4.51E-02	3.188	ATP8B4	intronic
2	rs6548077	2697985	p25.3	A	0.1579	0.0556	C	4.016	4.51E-02	3.188		
10	rs6585021	112640045	q25.2	T	0.1579	0.0556	C	4.016	4.51E-02	3.188	PDCD4	intronic
10	rs6585023	112658371	q25.2	A	0.1579	0.0556	G	4.016	4.51E-02	3.188	BBIP1	intronic
8	rs6985430	75884685	q21.11	G	0.1579	0.0556	A	4.016	4.51E-02	3.188		
17	rs7222964	57081718	q23.2	C	0.1579	0.0556	T	4.016	4.51E-02	3.188		
21	rs7277065	43969714	q22.3	T	0.1579	0.0556	C	4.016	4.51E-02	3.188	PDXK	intronic
4	rs7693445	174252199	q34.1	T	0.1579	0.0556	C	4.016	4.51E-02	3.188		
8	rs7842758	75870217	q21.11	G	0.1579	0.0556	A	4.016	4.51E-02	3.188		
6	rs9349160	40708620	p21.1	A	0.1579	0.0556	G	4.016	4.51E-02	3.188		
22	rs9615753	46857005	q13.32	T	0.1579	0.0556	C	4.016	4.51E-02	3.188		
5	rs9687338	19237824	p14.3	A	0.1579	0.0556	G	4.016	4.51E-02	3.188		
3	rs9849092	101293099	q12.1	A	0.1579	0.0556	G	4.016	4.51E-02	3.188	CMSS1/FLIP1L	intronic
16	rs12103381	62341057	q21	C	0.1429	0.0441	T	3.941	4.71E-02	3.611		
17	rs12948783	72010995	q25.1	A	0.1351	0.0417	G	3.931	4.74E-02	3.594	RHBDF2	5upstream
2	rs2037814	73529177	p13.1	A	0.1351	0.0417	C	3.931	4.74E-02	3.594	ALMS1	coding
13	rs7318721	23318440	q12.12	A	0.1351	0.0417	C	3.931	4.74E-02	3.594	MIPEP	intronic
13	rs12871083	86458485	q31.1	C	0.0857	0.0139	T	3.907	4.81E-02	6.656		
13	rs3847988	29401277	q12.3	G	0.0857	0.0139	T	3.907	4.81E-02	6.656		
6	rs12204791	3345768	p25.2	C	0.1571	0.0556	T	3.877	4.90E-02	3.169	SLC22A23	non-coding intronic
3	rs12489048	115258840	q13.31	G	0.1571	0.0556	A	3.877	4.90E-02	3.169	KIAA1407/QRTRTD1	5upstream/intronic
