# Supplementary material for: ASD Phenotype—Genotype Associations in Concordant and Discordant Monozygotic and Dizygotic Twins Stratified by Severity of Autistic Traits
Source: Int J Mol Sci. 2019 Aug 3;20(15):3804. doi: 10.3390/ijms20153804 (PMC6696087; doi:10.3390/ijms20153804)
Supplement: Supplementary file 1 [file ijms-20-03804-s001.zip › Supplementary Materials/Table S14. Demographic info of all twin samples from AGRE.pdf]

Table S14. Demographic information of all twins from AGRE

	Monozygotic			Dizygotic		
Concordant	Twin Pairs	88		Twin Pairs	56	
	Age Range	2.99-26.26		Age Range	2.74-15.72	
	Average Age	8.96		Average Age	6.94	
			# Individuals			# Individuals
	Female Pairs	12	24	Female Pairs	3	6
	Male Pairs	76	152	Male Pairs	33	66
	Mixed Pairs	0	0	Mixed Pairs	20	40
	Race (by pair)	3 Asian		Race (by pair)	3 Asian	
		8 Unknown			3 Unknown	
		67 White			46 White	
		7 More than One race			4 More than One Race	
		3 Black or African American			0 Black or African American	
Discordant	Twin Pairs	25		Twin Pairs	115	
	Age Range	NA:3.19-31.08	A:3.32-30.87	Age Range	NA:3.16-18.49	A:3.48-18.27
	Average Age	NA:11.39	A:11.33	Average Age	NA:9.18	A:9.20
			# Individuals			# Individuals
	Female Pairs	6	12	Female Pairs	8	16
	Male Pairs	19	38	Male Pairs	42	84
	Mixed Pairs	0	0	Mixed Pairs	65	130
	Race (by pair)	2 Asian		Race (by pair)	4 Asian	
		2 Unknown			5 Unknown	
		19 White			81 White	
		0 More than One Race			18 More than One Race	
		2 Black or African American			6 Black or African American	
					1 American Indian/Alaskan Native	
