# Supplementary material for: ASD Phenotype—Genotype Associations in Concordant and Discordant Monozygotic and Dizygotic Twins Stratified by Severity of Autistic Traits
Source: Int J Mol Sci. 2019 Aug 3;20(15):3804. doi: 10.3390/ijms20153804 (PMC6696087; doi:10.3390/ijms20153804)
Supplement: Supplementary file 1 [file ijms-20-03804-s001.zip › Supplementary Materials/Table S15. ADI-R items used for quantitative trait severity profiles and analyses.pdf]

Table S15. ADI-R items used for quantitative trait severity profiles*

Language	Nonverbal communication	Perseverative behavior	Play skills	Social interactions
CARTIC	CCOMPSL	CUNPROC	CPLAY	GAZE5
ARTICF5	COMPSL5	EUNPROC	PLAY5	CSSMILE
CSTEREO	CUSEBOD	CCIRINT	CPEERPL	SSMILE5
ESTEREO	EUSEBOD	ECIRINT	PEERPL5	CSHOW
CCHAT	CPOINT	CUSEOBJ	CSOPLAY	SHOW5
CHAT5	POINT5	EUSEOBJ	SOPLAY5	COSHARE
CCONVER	CNOD	CCRIT	CINTCH	OSHARE5
CONVER5	NOD5	ECRIT	INTCH5	CSHARE
CINAPPQ	CHSHAKE	CUNSENS	CRESPCH	SHARE5
EINAPPQ	HSHAKE5	EUNSENS	RESPCH5	COCOMF
CPRON	CINSGES	CCHANGE	CGRPLAY	OCOMF5
EPRON	INSGES5	ECHANGE	GRPLAY5	CQUALOV
CNEOID	AVOICE5	CRESIS	CFRIEND	QUALOV5
ENEOID	CIMIT	ERESIS	FREND15	CRFACEX
CVERRIT	IMIT5	CUATT		RFACEX5
EVERRIT		EUATT		CINAPFE
CINR				EINAPFE
EINR				CQRESP
CSPEECH				QRESP5
SPEECH5				CINITIA
				INITIA5
				CSOCDIS
				SOCDIS5

*From Hu and Steinberg [17]
