# Supplementary material for: ASD Phenotype—Genotype Associations in Concordant and Discordant Monozygotic and Dizygotic Twins Stratified by Severity of Autistic Traits
Source: Int J Mol Sci. 2019 Aug 3;20(15):3804. doi: 10.3390/ijms20153804 (PMC6696087; doi:10.3390/ijms20153804)
Supplement: Supplementary file 1 [file ijms-20-03804-s001.zip › Supplementary Materials/Table S2. All trait QTNs for dA.MZ twins.pdf]

All trait QTNs for dA.MZ twins								
**Language**								
CHR	SNP	BP	P	Band	Genes	Role	aa Position	aa Change
7	rs10275192	85436760	6.10E-08	q21.11				
16	rs11076384	59070568	6.10E-08	q21				
16	rs16962092	59068132	6.10E-08	q21				
9	rs2809445	127988530	6.10E-08	q33.3				
7	rs7785694	85431498	6.10E-08	q21.11				
1	rs2774285	114443208	1.11E-06	p13.2	SYT6	intronic		
13	rs9594670	41529489	3.30E-06	q14.11	DGKH	intronic		
11	rs1809063	131636242	7.55E-06	q25	NTM	intronic		
17	rs756785	27836958	8.43E-06	q11.2	CDK5R1	5upstream		
**Nonverbal**								
CHR	SNP	BP	P	Band	Genes	Role	aa Position	aa Change
10	rs1836470	45099325	2.03E-06	q11.21				
17	rs11080180	27707111	3.11E-06	q11.2	ZNF207	intronic		
7	rs6462639	36019937	7.48E-06	p14.2				
**Play**								
CHR	SNP	BP	P	Band	Genes	Role	aa Position	aa Change
18	rs8088744	64685792	7.23E-06	q22.1	CCDC102B	intronic		
2	rs355810	165393892	9.63E-06	q24.3	COBLL1	intronic		
**Social**								
CHR	SNP	BP	P	Band	Genes	Role	aa Position	aa Change
19	rs3826861	50748460	2.20E-06	p12.2	OPA3	3utr		
19	rs1264226	50755007	2.47E-06	p13.2	OPA3	intronic		
4	rs11723176	187648291	2.55E-06	p13.2	F11_AS1	non-coding intronic		
3	rs4856450	83388138	5.52E-06	q35.2				
1	rs11102627	113761409	6.60E-06	q13.32	MAGI3	intronic		

1	rs11102647	113872742	6.60E-06	q26.3	MAGI3	intronic		
10	rs521331	8264856	6.60E-06	q14.1				
10	rs7070581	126159363	6.60E-06	q13.32	LHPP	intronic		
12	rs10505863	20228698	7.94E-06	p12.2				
23	rs5962427	103575337	8.99E-06	p14				
23	rs2097402	133945220	9.05E-06	q22.2				
5	rs2287672	79125284	9.29E-06	q26.13	CMYA5	intronic		
**Perseverative Behaviors**								
CHR	SNP	BP	P	Band	Genes	Role	aa Position	aa Change
15	rs10518761	52322448	1.37E-06	q21.3	UNC13C	intronic		
