# Supplementary material for: ASD Phenotype—Genotype Associations in Concordant and Discordant Monozygotic and Dizygotic Twins Stratified by Severity of Autistic Traits
Source: Int J Mol Sci. 2019 Aug 3;20(15):3804. doi: 10.3390/ijms20153804 (PMC6696087; doi:10.3390/ijms20153804)
Supplement: Supplementary file 1 [file ijms-20-03804-s001.zip › Supplementary Materials/Table S3. All trait QTNs for dNA.MZ twins.pdf]

All trait QTNs for dNA.MZ twins								
**Language**								
CHR	SNP	BP	P	Band	Genes	Role	aa Position	aa Change
2	rs10490230	50391831	1.22E-07	p16.3	NRXN1	intronic		
12	rs11057881	123937926	1.22E-07	q24.31				
7	rs1553841	47510767	1.22E-07	p12.3	TNS3	intronic		
8	rs604302	37123727	1.22E-07	p11.23				
2	rs3791289	134827909	7.96E-07	q21.3	MGAT5	intronic		
4	rs10033190	118435301	9.69E-07	q26				
5	rs3896348	18204313	1.02E-06	p15.1				
7	rs10238376	30940878	1.31E-06	p14.3				
8	rs2618806	2111558	1.31E-06	p23.3				
2	rs6742060	238234971	1.31E-06	q37.3	LRRFIP1	intronic		
2	rs1568854	149633823	1.50E-06	q23.2	LYPD6B	intronic		
12	rs1996749	102245143	1.65E-06	q23.2	C12orf42	intronic		
15	rs12915265	30196358	2.16E-06	q13.3	CHRNA7	non-coding intronic		
11	rs544978	62441806	2.16E-06	q12.3	CHRM1	intronic		
6	rs4443522	129211626	3.37E-06	q22.33				
6	rs4487603	129211602	3.37E-06	q22.33				
1	rs783036	66603958	3.37E-06	p31.3	PDE4B	coding	1451	E>E
11	rs11041537	7671750	4.04E-06	p15.4	OVCH2	intronic		
5	rs12521575	18187934	4.04E-06	p15.1				
3	rs4521229	181973610	4.04E-06	q26.33	LOC101928882	non-coding intronic		
5	rs923963	62075399	4.04E-06	q12.1				
6	rs12525297	107246561	4.21E-06	q21				
1	rs3766793	53363218	4.21E-06	p32.3	SLC1A7	non-coding intronic		
14	rs10151666	33007535	4.34E-06	q13.1	NPAS3	intronic		
8	rs10956504	131051089	5.25E-06	q24.21	FAM49B	non-coding intronic		
8	rs921693	131074193	5.72E-06	q24.21	FAM49B	non-coding intronic		
**Nonverbal**								

CHR	SNP	BP	P	Band	Genes	Role	aa Position	aa Change
6	rs1414743	142319462	5.21E-07	q24.1	AK097143	intronic		
6	rs2185503	8294510	5.21E-07	p24.3				
19	rs4808521	16646721	5.21E-07	p13.11	TMEM38A	intronic		
11	rs7951632	110358395	5.21E-07	q23.1				
4	rs10017860	16203977	7.66E-07	p15.32	LDB2/MIR548AX	intronic/3downstream		
20	rs2300216	3894057	8.15E-07	p13	RNF24	intronic		
3	rs3872649	245521	8.15E-07	p26.3	CHL1	intronic		
19	rs7250000	56578734	8.15E-07	q13.41	LIM2	intronic		
10	rs1931901	7695358	9.43E-07	p14	ITIH5	intronic		
12	rs10777986	98353626	9.62E-07	q23.1	ANKS1B	intronic		
11	rs11215438	114616921	1.26E-06	q23.3	CADM1	intronic		
22	rs739140	36445502	1.26E-06	q13.1	TRIOBP	intronic		
4	rs10937638	5428143	1.47E-06	p16.2	STK32B	intronic		
1	rs11120063	211141648	1.80E-06	q32.3				
1	rs1284851	211139989	1.80E-06	q32.3	FLVCR1	3downstream		
5	rs42914	36771041	1.80E-06	p13.2				
3	rs7628531	72684645	1.80E-06	p13				
6	rs2256028	31487177	2.11E-06	p21.33	MICA	intronic		
17	rs11079427	56810004	3.92E-06	q23.2	BCAS3	intronic		
1	rs16833356	234358516	3.92E-06	q42.3				
3	rs2010479	127860189	3.92E-06	q21.3	NUP210P1	5upstream		
2	rs6745492	237172556	3.92E-06	q37.3				
4	rs737601	9762331	3.92E-06	p16.1				
3	rs7628233	127878243	3.92E-06	q21.3				
13	rs7320823	67575838	4.55E-06	q21.33				
2	rs2072474	102005641	4.85E-06	q11.2	IL1R2	non-coding intronic		
8	rs6471603	58517186	6.43E-06	q12.1				
20	rs6062894	61425120	9.83E-06	q13.33	COL20A1	intronic		
6	rs663614	87491867	9.83E-06	q14.3				
**Play**								

CHR	SNP	BP	P	Band	Genes	Role	aa Position	aa Change
3	rs333327	125813434	2.67E-06	q21.2	KALRN	intronic		
1	rs485874	18945513	3.81E-06	p36.13	PAX7	3utr		
10	rs2505956	27225292	4.24E-06	p12.1				
11	rs713065	86335168	5.06E-06	q14.2	FZD4	3utr		
12	rs4523751	89842084	6.06E-06	q21.33	LINC00615	non-coding intronic		
4	rs2725769	105975618	9.03E-06	q24	RP11-556I14.2	non-coding intronic		
4	rs2725771	105978188	9.03E-06	q24	RP11-556I14.2	non-coding intronic		

Social

CHR	SNP	BP	P	Band	Genes	Role	aa Position	aa Change
2	rs6717613	299480	3.07E-07	p25.3	AC079779.5	5upstream		
6	rs6902999	105866239	5.43E-07	q21	PREP	intronic		
8	rs11987687	123131375	5.83E-07	q24.13				
13	rs1421296	105057211	5.83E-07	q33.2				
7	rs2941543	47492265	1.33E-06	p12.3	TNS3	intronic		
14	rs10131032	32319832	2.00E-06	q12	AKAP6	intronic		
7	rs10252217	53387975	2.00E-06	p12.1				
7	rs1034784	82062116	2.00E-06	q21.11				
12	rs10507071	95280908	2.00E-06	q23.1	CDK17	intronic		
11	rs10830430	89541802	2.00E-06	q14.3	NAALAD2	coding	446	I>V
7	rs10953849	117174344	2.00E-06	q31.2	CTTNBP2	intronic		
4	rs11097637	99963878	2.00E-06	q23				
7	rs11767812	117206428	2.00E-06	q31.31	CTTNBP2	intronic		
6	rs12179534	18572627	2.00E-06					
1	rs1329465	99155952	2.00E-06	p21.3	LPPR5	intronic		
6	rs1408267	18530528	2.00E-06	p22.3	RNF144B	intronic		
12	rs1440092	95273987	2.00E-06	q23.1	CDK17	intronic		
2	rs16844650	196653630	2.00E-06	q32.3				
20	rs16989457	39011296	2.00E-06	q12				
19	rs2075604	1169523	2.00E-06	p13.3	STK11	intronic		
3	rs2371121	27825729	2.00E-06	p24.1	AC098973.2	non-coding intronic		

3	rs2618111	27886915	2.00E-06	p24.1	AC098973.2	non-coding intronic		
9	rs328012	107077538	2.00E-06	q31.1	SLC44A1	intronic		
3	rs3907388	2009933	2.00E-06	p26.3				
2	rs4395260	196675582	2.00E-06	q32.3				
4	rs4401453	99993206	2.00E-06	q23				
12	rs4608180	95165979	2.00E-06	q23.1	ELK3	intronic		
6	rs567698	18562822	2.00E-06	p22.3	RNF144B	intronic		
18	rs663830	10594362	2.00E-06	p11.22				
15	rs7183453	69641001	2.00E-06	q23	THSD4	intronic		
17	rs7208605	11222570	2.00E-06	p12	SHISA6	intronic		
12	rs7976881	95315077	2.00E-06	q23.1	CDK17	intronic		
17	rs8068118	11256344	2.00E-06	p12	SHISA6	intronic		
6	rs9501714	135248	2.00E-06	p25.3	RP3-416J7.4	non-coding intronic		
14	rs1599965	94944160	2.67E-06	q32.13	LINC00341	non-coding		
10	rs771874	36266482	3.54E-06	p11.21				
3	rs717939	18601746	4.63E-06	p24.3	AC144521.1	non-coding intronic		
9	rs2007744	78044132	7.68E-06	q21.13	PCSK5	intronic		
16	rs2911174	62214791	8.60E-06	q21				
8	rs1044783	416685	9.25E-06	p23.3	RP11-91J19.4	non-coding		
**Perseverative Behaviors**								
CHR	SNP	BP	P	Band	Genes	Role	aa Position	aa Change
2	rs10191517	73411911	1.35E-06	p13.1				
2	rs4641966	73444314	1.35E-06	p13.1				
2	rs6546824	73440839	1.35E-06	p13.1				
23	rs1974522	3248733	1.72E-06	p22.33	MXRA5	coding	1665	P>S|A
11	rs2509094	95360556	1.76E-06	q21	MAML2	intronic		
11	rs10891615	113381063	2.80E-06	q23.2				
16	rs410059	78212573	4.25E-06	q23.2				
12	rs10735937	65340149	5.17E-06	q14.3	GRIP1	intronic		
11	rs4938066	113390622	6.37E-06	q23.2				
9	rs7031476	15047624	7.73E-06	p22.3	RP11-54D18.4	3downstream		

8	rs7828540	72258791	8.12E-06	q13.3	RP11-326E22.1	non-coding intronic		
13	rs10508006	92952411	8.33E-06	q31.3	GPC6	intronic		
10	rs10749565	89882080	8.33E-06	q23.31	RNLS	3utr		
8	rs184487	26404795	9.59E-06	p21.2	BNIP3L	intronic		
8	rs189025	26393432	9.59E-06	p21.2	BNIP3L	intronic		
11	rs7395845	70920892	9.59E-06	q13.4				
