# Supplementary material for: ASD Phenotype—Genotype Associations in Concordant and Discordant Monozygotic and Dizygotic Twins Stratified by Severity of Autistic Traits
Source: Int J Mol Sci. 2019 Aug 3;20(15):3804. doi: 10.3390/ijms20153804 (PMC6696087; doi:10.3390/ijms20153804)
Supplement: Supplementary file 1 [file ijms-20-03804-s001.zip › Supplementary Materials/Table S4. All trait QTNs for cDZ twins.pdf]

All trait QTNs for cDZ twins								
**Language**								
CHR	SNP	BP	P	Band	Genes	Role	aa Position	aa Change
16	rs4011585	71914015	2.89E-08	16q22.3				
16	rs4888646	71920088	2.89E-08	16q22.3				
7	rs2106740	89092149	9.57E-08	7q21.13				
18	rs4891775	65627430	1.49E-06					
5	rs2578539	5547739	1.55E-06	5p15.32	KIAA0947	Downstream	-	-
2	rs13425437	116550707	1.78E-06	2q14.1				
2	rs17543960	116586663	1.78E-06	2q14.1				
3	rs11917359	25673708	2.21E-06	3p24.2	TOP2B	Intron	-	-
14	rs17127374	91017796	2.25E-06	14q32.12	SMEK1	Coding exon	T/T	264
21	rs2837366	40301653	2.37E-06	21q22.2	DSCAM	Downstream	-	-
7	rs2058002	25406658	2.74E-06	7p15.3				
5	rs2578500	5515148	3.80E-06	5p15.32	KIAA0947	Coding exon	V/I	901
5	rs1559286	42787794	4.25E-06	5p12	CCDC152	Promoter	-	-
13	rs1887263	22381716	4.63E-06	13q12.12				
21	rs2026886	45655301	5.05E-06	21q22.3	NCRNA00175	Downstream	-	-
16	rs1886714	19970009	5.23E-06	16p12.3	GPR139	Intron	-	-
11	rs184267	1945152	5.79E-06	11p15.5				
4	rs10520554	184446191	6.17E-06	4q35.1	WWC2	Intron	-	-
2	rs2889606	105921422	6.84E-06	2q12.2				
15	rs7177755	60886781	7.51E-06	15q22.2	TLN2	Intron	-	-
7	rs11975137	88988845	8.15E-06	7q21.13				
7	rs7794184	89052607	8.15E-06	7q21.13				
10	rs10827492	35469831	9.36E-06	10p11.21	CREM	Intron	-	-
10	rs4934540	35514705	9.36E-06	10p11.21	CREM	Intron	-	-
1	rs357044	178506096	9.50E-06	1q25.2	LHX4	Intron	-	-
15	rs7162186	30973508	9.83E-06	15q13.3	FMN1	Intron	-	-
**Nonverbal**								
CHR	SNP	BP	P	Band	Genes	Role	aa Position	aa Change
14	rs12881439	36175604	7.96E-06	14q13.3				
14	rs11848377	36178269	7.96E-06	14q13.3				

**Play**								
CHR	SNP	BP	P	Band	Genes	Role	aa Position	aa Change
10	rs1171582	61164043	5.84E-07	q21.2	SLC16A9	intronic		
6	rs4707485	89294410	1.48E-06	q15	LOC101928936	non-coding		
7	rs1180774	97475759	1.56E-06	q21.3				
6	rs2610763	89313184	2.52E-06	q15				
2	rs6717613	299480	4.00E-06	p25.3	AC079779.5	5upstream/intronic/3downstream		
6	rs976145	81463977	4.71E-06	q14.1				
8	rs6982697	29938457	5.68E-06	p12				
16	rs8060975	15784912	6.80E-06	p13.11	MYH11	intronic/5upstream/3dowmstream		
6	rs1407220	123934131	7.82E-06	q22.31	TRDN	intronic		
6	rs9295192	162900893	9.11E-06	q26	PARK2	intronic		
5	rs9329125	177334115	9.84E-06	q35.3	RP11-1252I4.2	non-coding intronic		
**Social**								
CHR	SNP	BP	P	Band	Genes	Role	aa Position	aa Change
10	rs1171582	61164043	5.84E-07	10q21.2	SLC16A9	intronic		
6	rs4707485	89294410	1.48E-06	89294410				
7	rs1180774	97475759	1.56E-06	97475759				
6	rs2610763	89313184	2.52E-06	89313184				
2	rs6717613	299480	4.00E-06	2p25.3				
6	rs976145	81463977	4.71E-06	81463977				
8	rs6982697	29938457	5.68E-06	29938457				
16	rs8060975	15784912	6.80E-06	15784912	MYH11	intronic		
6	rs1407220	123934131	7.82E-06	1.24E+08	TRDN	intronic		
6	rs9295192	162900893	9.11E-06	1.63E+08	PARK2	intronic		
5	rs9329125	177334115	9.84E-06	1.77E+08				
**Perseverative Behavior**								
CHR	SNP	BP	P	Band	Genes	Role	aa Position	aa Change
18	rs662397	42574834	7.48E-08	18q21.1	ST8SIA5	Intron	-	-
11	rs12146477	102130166	3.01E-07	11q22.2				
11	rs2846714	102031820	3.37E-07	11q22.2				

20	rs6039104	8164697	7.57E-07	20p12.3	PLCB1	Intron	-	-
18	rs9675544	42570717	1.17E-06	18q21.1	ST8SIA5	Intron	-	-
14	rs2331779	68151670	3.11E-06	14q24.1				
14	rs8014901	68159378	3.37E-06	14q24.1				
13	rs9562157	113999458	4.17E-06	13q34				
8	rs4875505	5149306	5.22E-06	8p23.2				
4	rs12500644	138941802	5.86E-06	4q28.3				
21	rs7281374	34423844	7.12E-06	21q22.11	MRPS6	Intron	-	-
1	rs7547507	15494295	8.44E-06	1p36.21	FHAD1	Intron	-	-
9	rs1340007	1314127	8.83E-06	9p24.3				
6	rs1040521	2984608	9.15E-06	6p25.2	FAM136B	Promoter	-	-
6	rs7774283	2986199	9.15E-06	6p25.2	FAM136B	Promoter	-	-
