# Supplementary material for: ASD Phenotype—Genotype Associations in Concordant and Discordant Monozygotic and Dizygotic Twins Stratified by Severity of Autistic Traits
Source: Int J Mol Sci. 2019 Aug 3;20(15):3804. doi: 10.3390/ijms20153804 (PMC6696087; doi:10.3390/ijms20153804)
Supplement: Supplementary file 1 [file ijms-20-03804-s001.zip › Supplementary Materials/Table S5. All trait QTNs for dA.DZ twins.pdf]

All trait QTNs for dA.DZ twins								
**Language**								
CHR	SNP	BP	P	Band	Genes	Role	aa Position	aa Change
15	rs1823718	71934297	2.01E-06	q24.1				
1	rs17587765	117372602	3.53E-06	p13.1	CD101	intronic		
12	rs249047	112562237	9.96E-06	q24.13				
**Nonverbal**								
CHR	SNP	BP	P	Band	Genes	Role	aa Position	aa Change
6	rs6923735	3362642	1.03E-06	p25.2	SLC22A23	intronic		
11	rs2953310	40094119	6.01E-06	p12	LRRC4C	coding	96	R>R
3	rs7613095	194294915	7.35E-06	q29				
6	rs807302	119968450	7.68E-06	q22.31				
**Play**								
CHR	SNP	BP	P	Band	Genes	Role	aa Position	aa Change
12	rs17483048	118415784	8.70E-08	q24.23	CCDC60/AF086288	non-coding intronic		
10	rs17692734	54841584	3.90E-07	q21.1				
10	rs2133470	54807327	3.48E-06	q21.1				
3	rs1563981	28722162	6.95E-06	p24.1	LINC00693	non-coding intronic		
16	rs17772954	76243191	9.87E-06	q23.1				
**Social**								
CHR	SNP	BP	P	Band	Genes	Role	aa Position	aa Change
11	rs2953310	40094119	1.37E-06	p12	LRRC4C	coding	96/100	R>R
14	rs12894797	79861843	7.21E-06	q31.1	DIO2-AS1			
**Perseverative Behaviors**								
CHR	SNP	BP	P	Band	Genes	Role	aa Position	aa Change
11	rs11219808	124225108	3.98E-07	q24.2				

10	rs11818084	125976888	1.50E-06	q26.13				
17	rs1010045	67801683	2.37E-06	q24.3				
18	rs8084650	36009793	3.90E-06	q12.3				
6	rs6454752	90075080	4.82E-06	q15	GABRR2	intronic		
10	rs12570126	75086795	5.25E-06	q22.2	SYNPO2L	5upstream/noncoding intronic		
3	rs2679593	157706326	5.90E-06	q25.31	KCNAB1	intronic		
