# Supplementary material for: ASD Phenotype—Genotype Associations in Concordant and Discordant Monozygotic and Dizygotic Twins Stratified by Severity of Autistic Traits
Source: Int J Mol Sci. 2019 Aug 3;20(15):3804. doi: 10.3390/ijms20153804 (PMC6696087; doi:10.3390/ijms20153804)
Supplement: Supplementary file 1 [file ijms-20-03804-s001.zip › Supplementary Materials/Table S6. All trait QTNs for dNA.DZ twins.pdf]

All trait QTNs for dNA.DZ twins								
**Language**								
CHR	SNP	BP	P	Band	Genes	Role	aa Position	aa Change
18	rs7232149	10937468	8.19E-12	p11.21	PIEZO2	intronic		
18	rs9952954	10939093	8.19E-12	p11.21	PIEZO2	intronic		
1	rs1555026	23759792	4.66E-09	p36.12	ID3	5upstream		
1	rs877518	23767741	4.66E-09	p36.12				
3	rs1822395	158755076	1.80E-08	q25.32	C3orf55	intronic		
13	rs10507627	58485722	1.80E-08	q21.1				
2	rs384180	6483994	2.50E-08	p25.2				
2	rs10498133	222812549	2.50E-08	q36.1	PAX3	intronic		
4	rs17005123	82534494	2.50E-08	q21.21				
5	rs16902290	34522293	2.50E-08	p13.2				
6	rs17287803	42039979	2.50E-08	p21.1	CCND3	intronic		
8	rs16868879	103095400	2.50E-08	q22.3	NCALD	intronic		
12	rs7135813	85718641	2.50E-08	q21.32	MGAT4C	intronic		
14	rs1957173	45476666	2.50E-08	q21.2				
18	rs401127	6305088	2.50E-08	p11.31	L3MBTL4	intronic		
18	rs236035	6320209	2.50E-08	p11.31	L3MBTL4	intronic		
12	rs12812815	85758126	3.97E-08	q21.32	MGAT4C	5upstream		
8	rs11984453	4491939	5.48E-08	p23.2	CSMD1	intronic		
5	rs7444875	34520901	6.58E-08	p13.2				
3	rs17035408	36662904	7.19E-08	p22.2				
2	rs2216328	56260472	8.74E-08	p16.1	AC007743.1/RP1	non-coding		
2	rs2138380	202877469	1.48E-07	q33.1	NOP58	3downstream		
2	rs2350807	203004035	1.48E-07	q33.1	BMPR2	intronic		
2	rs12987781	203078111	1.48E-07	q33.2	BMPR2	intronic		
2	rs17199249	203134913	1.48E-07	q33.2	BMPR2	3utr		
12	rs2053629	39429400	1.78E-07	q12	CNTN1	intronic		
12	rs310830	75943724	4.16E-07	q21.2	E2F7	coding	770	S>S
7	rs10487202	103791913	6.02E-07	q22.2	LHFPL3	intronic		
13	rs17087794	70445442	6.81E-07	q21.33				

6	rs228139	55736242	8.25E-07	p12.1	BMP5	intronic		
21	rs2032314	34276393	9.07E-07	q22.11				
2	rs1861773	56256352	1.18E-06	p16.1	AC007743.1/RP1[illegible]	non-coding		
2	rs934374	23619790	1.27E-06	p24.1	KLHL29	intronic		
4	rs13110248	166331362	1.27E-06	q32.3	TMEM192	intronic		
8	rs10095586	78887533	1.27E-06	q21.12				
11	rs634416	117085025	1.27E-06	q23.3	DSCAML1	intronic		
12	rs7302057	39469139	1.27E-06	q12	CNTN1	intronic		
12	rs6581959	39470317	1.27E-06	q12	CNTN1	intronic		
12	rs1965340	67599196	1.27E-06	q15	CPM	intronic		
17	rs9913935	25876414	1.27E-06	q11.2	GOSR1	3utr		
17	rs11650766	25886393	1.27E-06	q11.2	ALOX12P1	intronic		
17	rs11653789	25892085	1.27E-06	q11.2	ALOX12P1	intronic		
17	rs8081187	26040486	1.27E-06	q11.2				
1	rs704787	39972441	1.48E-06	p34.2	PPIE	intronic		
2	rs6756156	56285098	1.85E-06	p16.1	CCDC85A	intronic		
2	rs6733298	56304360	1.85E-06	p16.1	CCDC85A	intronic		
2	rs6545561	56310240	1.85E-06	p16.1	CCDC85A	intronic		
16	rs17806253	23576791	1.88E-06	p12.2	DCTN5	intronic		
1	rs347273	160584137	2.40E-06	q23.3	NOS1AP	intronic		
14	rs1042897	99818376	2.60E-06	q32.2	YY1/AL157871.2	3utr/intronic		
7	rs6972034	103804953	2.77E-06	q22.2	LHFPL3	intronic		
6	rs12197404	107416824	2.80E-06	q21				
6	rs9398330	113500683	2.80E-06	q21				
8	rs1283703	108472658	2.98E-06	q23.1	ANGPT1	intronic		
12	rs17680262	108838919	3.87E-06	q24.11	TCHP	3utr		
18	rs12963818	47283465	3.87E-06	q21.2	RP11_267C16.1	intronic		
2	rs11676424	239338075	4.36E-06	q37.3				
12	rs1444204	39369149	4.53E-06	q12				
12	rs1584093	39408555	4.53E-06	q12	CNTN1	intronic		
23	rs6629669	23454170	4.95E-06	p22.11				
12	rs310803	75966542	5.04E-06	q21.2	E2F7	intronic		

4	rs4308418	188450860	5.09E-06	q35.2				
2	rs2869528	56312931	5.18E-06	p16.1	CCDC85A	intronic		
2	rs214047	56344184	5.18E-06	p16.1	CCDC85A	intronic		
8	rs17595454	18377451	5.72E-06	p22				
10	rs10823960	53446370	5.80E-06	q21.1	PAX3	intronic		
10	rs12777779	127850057	5.98E-06	q26.2	ADAM12	intronic		
2	rs13413035	40189400	6.12E-06	p22.1	SLC8A1_AS1	intronic		
2	rs11679833	121023152	6.12E-06	q14.2				
3	rs11921464	2220103	6.12E-06	p26.3	CNTN4	intronic		
6	rs3129727	32787668	6.12E-06	p21.32				
8	rs11990353	118113463	6.12E-06	q24.11	SLC30A8	intronic		
8	rs16905064	135133983	6.12E-06	q24.22				
12	rs2701815	126984005	6.12E-06	q24.32	LINC00507	intronic		
12	rs12812393	131026648	6.12E-06	q24.33	EP400	intronic		
23	rs4459029	67773144	6.12E-06	q13.1				
20	rs2427386	60794328	6.27E-06	q13.33				
7	rs16873582	138246541	6.34E-06	q34	KIAA1549	coding	1012	S>S
5	rs10042047	125338510	7.18E-06	q23.2				
9	rs16921254	103885104	7.18E-06	q31.1				
17	rs2314640	9242675	8.06E-06	p13.1	STX8	intronic		
23	rs6648168	72391114	8.25E-06	q13.2				
2	rs7426302	231237153	8.92E-06	q37.1				
22	rs6519702	25883046	9.39E-06	q12.1	CTA-992D9.7	intronic		
7	rs10281008	27844062	9.66E-06	p15.2	JAZF1	intronic		
9	rs10970527	31781211	9.73E-06	p21.1				
8	rs1584220	117997118	9.87E-06	q24.11				
8	rs17744529	118000035	9.87E-06	q24.11				
8	rs4319130	118002365	9.87E-06	q24.11				
**Nonverbal**								
CHR	SNP	BP	P	Band	Genes	Role	aa Position	aa Change
2	rs12987781	203078111	6.96E-11	q33.2	BMPR2	intronic		

2	rs17199249	203134913	6.96E-11	q33.2	BMPR2	3utr		
2	rs2138380	202877469	6.96E-11	q33.1	NOP58	3downstream		
2	rs2350807	203004035	6.96E-11	q33.1	BMPR2	intronic		
2	rs6545561	56310240	3.50E-08	p16.1	CCDC85A	intronic		
2	rs6733298	56304360	3.50E-08	p16.1	CCDC85A	intronic		
2	rs6756156	56285098	3.50E-08	p16.1	CCDC85A	intronic		
9	rs6479471	95084239	5.27E-08	q22.31	WNK2	intronic		
9	rs7022051	95042804	5.27E-08	q22.31	WNK2	intronic		
23	rs1128863	154010056	6.69E-08	q28	MTCP1	intronic		
5	rs17097187	140692037	6.69E-08	q31.3	PCDHGA1	coding	534	A>A
23	rs237536	142819240	6.69E-08	q27.3				
23	rs5909473	18329671	6.69E-08	p22.13				
22	rs6005657	26565087	1.09E-07	q12.1				
6	rs11753940	64500431	1.10E-07	q12	EYS	intronic		
19	rs11882691	36774875	1.10E-07	q12	THEG5	5upstream/intronic		
4	rs16895825	17435354	1.10E-07	p15.31	NCAPG	non-coding intronic		
4	rs16895939	17488259	1.10E-07	p15.31	LCORL	intronic		
12	rs17041392	107206906	1.10E-07	q23.3	CMKLR1	3utr		
4	rs17051351	122562025	1.10E-07	q27				
7	rs17148818	24122943	1.10E-07	p15.3				
3	rs17526839	25580105	1.10E-07	p24.2	RARB	intronic		
7	rs3025153	24226447	1.10E-07	p15.3	AC004485.3	non-coding intronic		
2	rs3914642	45257987	1.10E-07	p21	LINC01121	non-coding intronic		
3	rs6782829	136438993	1.10E-07	q22.2	EPHB1	intronic		
4	rs7690457	17414477	1.10E-07	p15.31	DCAF16	coding	129	T>I
7	rs10224816	9678184	1.12E-07	p21.3				
5	rs13169647	41468881	1.57E-07	p13.1	PLCXD3	intronic		
4	rs1355161	126487591	1.73E-07	q28.1	FAT4	intronic		
11	rs12804520	83368254	1.88E-07	q14.1	DLG2	intronic		
11	rs2242312	129780556	1.88E-07	q24.3	ADAMTS8	3utr		
10	rs772822	80304750	1.99E-07	q22.3				
12	rs12812815	85758126	3.89E-07	q21.32	MGAT4C	5upstream		

2	rs11676424	239338075	5.14E-07	q37.3				
2	rs10498133	222812549	6.31E-07	q36.1	PAX3	intronic		
8	rs16868879	103095400	6.31E-07	q22.3	NCALD	intronic		
5	rs16902290	34522293	6.31E-07	p13.2				
4	rs17005123	82534494	6.31E-07	q21.21				
6	rs17287803	42039979	6.31E-07	p21.1	CCND3	intronic		
14	rs1957173	45476666	6.31E-07	q21.2				
18	rs236035	6320209	6.31E-07	p11.31	L3MBTL4	intronic		
2	rs384180	6483994	6.31E-07	p25.2				
18	rs401127	6305088	6.31E-07	p11.31	L3MBTL4	intronic		
12	rs7135813	85718641	6.31E-07	q21.32	MGAT4C	intronic		
2	rs6728727	165977043	6.93E-07	q24.3				
4	rs17005129	82543901	7.68E-07	q21.21				
17	rs9897203	59960605	7.68E-07	q23.3	CEP95	intronic		
22	rs6519702	25883046	8.09E-07	q12.1	CTA-992D9.7	non-coding intronic		
18	rs7232149	10937468	8.71E-07	p11.21	PIEZO2	intronic		
18	rs9952954	10939093	8.71E-07	p11.21	PIEZO2	intronic		
2	rs7426302	231237153	8.74E-07	q37.1				
9	rs7857220	95064873	8.75E-07	q22.31	WNK2	intronic		
9	rs7874101	95031716	9.95E-07	q22.31	WNK2	intronic		
9	rs12216907	78225879	1.24E-06	q21.13	RP11-66C24.1	3downstream		
9	rs12216907	78225879	1.24E-06	q21.13	GCNT1	non-coding intronic		
5	rs7444875	34520901	1.29E-06	p13.2				
3	rs17564921	148372345	1.63E-06	q24	RP11-649A16.1	non-coding intronic		
8	rs11984453	4491939	1.67E-06	p23.2	CSMD1	intronic		
5	rs17211823	128158012	1.67E-06	q23.3	SLC27A6	intronic		
23	rs6629669	23454170	1.80E-06	p22.11				
10	rs497204	80306297	1.89E-06	q22.3				
2	rs11883890	231230076	1.94E-06	q37.1				
9	rs10992681	95018086	2.21E-06	q22.31	WNK2	intronic		
11	rs7123143	80683168	2.42E-06	q14.1				
2	rs7423933	56269275	2.44E-06	p16.1	CCDC85A	intronic		

22	rs17806754	32417553	2.85E-06	q12.3	LARGE	intronic		
7	rs10263571	15421858	3.25E-06	p21.2	AGMO	intronic		
7	rs4721438	15422074	3.25E-06	p21.2	AGMO	intronic		
4	rs11098654	123128865	4.68E-06	q27				
2	rs1437871	197540123	4.68E-06	q33.1	ANKRD44	3utr		
3	rs17195143	25042378	4.68E-06	p24.2	RNA5SP125	3downstream		
3	rs17195143	25042378	4.68E-06	p24.2	AC133680.1	non-coding intronic		
2	rs2376103	139430374	4.68E-06	q22.1				
2	rs4954642	139425722	4.68E-06	q22.1				
13	rs1475201	44347321	4.88E-06	q14.12				
1	rs1854077	199819461	4.88E-06	q32.1				
3	rs2314042	155736835	4.88E-06	q25.2	RP11-656A15.1	non-coding intronic		
11	rs4567442	42610618	4.88E-06	p12				
3	rs7636993	36421322	4.88E-06	p22.3	STAC	intronic		
4	rs2342654	156369261	4.98E-06	q32.1				
2	rs7559811	231231148	5.61E-06	q37.1				
18	rs8084568	57190720	6.31E-06	q21.33	CDH20	intronic		
17	rs4387653	68659971	6.57E-06	q25.1				
20	rs17805193	59138980	6.71E-06	q13.33				
2	rs1861773	56256352	6.99E-06	p16.1	AC007743.1	non-coding		
2	rs1861773	56256352	6.99E-06	p16.1	RP11-481J13.1	non-coding intronic		
3	rs10935663	148442795	8.98E-06	q24	RP11-649A16.1	non-coding intronic		
9	rs16928804	128033773	9.32E-06	q33.3				
7	rs4719155	70745160	9.55E-06	q11.22	WBSCR17	intronic		
6	rs6454694	89230477	9.87E-06	q15				
13	rs1220582	23639868	9.90E-06	q12.12	SPATA13	intronic		
9	rs7025467	137199567	9.90E-06	q34.3				
4	rs1465039	123187512	9.96E-06	q27				
4	rs1965582	123167389	9.96E-06	q27				

Play

CHR	SNP	BP	P	Band	Genes	Role	aa Position	aa Change

2	rs12987781	203078111	5.76E-11	q33.2	BMPR2	intronic		
2	rs17199249	203134913	5.76E-11	q33.2	BMPR2	3utr		
2	rs2138380	202877469	5.76E-11	q33.1	NOP58	3downstream		
2	rs2350807	203004035	5.76E-11	q33.1	BMPR2	intronic		
2	rs6545561	56310240	1.50E-10	p16.1	CCDC85A	intronic		
2	rs6733298	56304360	1.50E-10	p16.1	CCDC85A	intronic		
2	rs6756156	56285098	1.50E-10	p16.1	CCDC85A	intronic		
11	rs7123143	80683168	3.10E-09	q14.1				
2	rs11676424	239338075	9.75E-09	q37.3				
2	rs11673727	239311421	4.00E-08	q37.3	AC113618.1	non-coding intronic		
3	rs4488776	196013731	4.00E-08	q29				
2	rs17356755	183290415	5.97E-08	q32.1	DNAJC10	non-coding intronic		
12	rs12812815	85758126	1.10E-07	q21.32	MGAT4C	5upstream		
4	rs1391758	54429268	1.30E-07	q12	FIP1L1	intronic		
20	rs4809499	60788088	1.30E-07	q13.33	SLCO4A1	3downstream		
10	rs772822	80304750	1.30E-07	q22.3				
13	rs2876807	61019136	2.11E-07	q21.2				
5	rs13169647	41468881	2.22E-07	p13.1	PLCXD3	intronic		
5	rs10474916	11346094	2.68E-07	p15.2	CTNND2	intronic		
3	rs2673505	21845421	3.62E-07	p24.3	AC023798.1	5upstream		
3	rs2673505	21845421	3.62E-07	p24.3	ZNF385D	non-coding intronic		
3	rs2673529	21852365	3.62E-07	p24.3	ZNF385D	non-coding intronic		
3	rs1471659	128295267	4.03E-07	q21.3				
21	rs9978194	34176163	4.04E-07	q22.11	ITSN1	intronic		
6	rs11753940	64500431	5.11E-07	q12	EYS	intronic		
19	rs11882691	36774875	5.11E-07	q12	THEG5	intronic		
4	rs16895825	17435354	5.11E-07	p15.31	NCAPG	intronic		
4	rs16895939	17488259	5.11E-07	p15.31	LCORL	intronic		
12	rs17041392	107206906	5.11E-07	q23.3	CMKLR1	3utr		
4	rs17051351	122562025	5.11E-07	q27				
7	rs17148818	24122943	5.11E-07	p15.3				
3	rs17526839	25580105	5.11E-07	p24.2	RARB	intronic		

7	rs3025153	24226447	5.11E-07	p15.3	AC004485.3	non-coding intronic		
2	rs3914642	45257987	5.11E-07	p21	LINC01121	non-coding intronic		
3	rs6782829	136438993	5.11E-07	q22.2	EPHB1	intronic		
4	rs7690457	17414477	5.11E-07	p15.31	DCAF16	coding	129	T>I
9	rs10989224	102525990	5.70E-07	q31.1				
17	rs2676801	45048685	5.82E-07	q21.33	SPOP	intronic		
17	rs4794061	45131084	5.82E-07	q21.33				
2	rs2216328	56260472	5.89E-07	p16.1	non-coding intronic			
2	rs2216328	56260472	5.89E-07	p16.1	AC007743.1	non-coding		
3	rs17035408	36662904	6.15E-07	p22.2				
23	rs1128863	154010056	6.17E-07	q28	MTCP1	non-coding intronic		
5	rs17097187	140692037	6.17E-07	q31.3	PCDHGA1	coding	534	A>A
23	rs237536	142819240	6.17E-07	q27.3				
23	rs5909473	18329671	6.17E-07	p22.13				
2	rs10498133	222812549	6.40E-07	q36.1	PAX3	intronic		
1	rs12064875	108006971	6.40E-07	p13.3	VAV3	intronic		
1	rs12563935	230263968	6.40E-07	q42.2				
7	rs1636410	148725957	6.40E-07	q36.1	AC004941.3	5upstream		
1	rs16856351	230247195	6.40E-07	q42.2				
1	rs16856377	230258335	6.40E-07	q42.2				
8	rs16868879	103095400	6.40E-07	q22.3	NCALD	intronic		
5	rs16880156	52084715	6.40E-07	q11.2	CTD-2288O8.1	non-coding intronic		
5	rs16902290	34522293	6.40E-07	p13.2				
4	rs17005123	82534494	6.40E-07	q21.21				
4	rs17046561	166639887	6.40E-07	q32.3	CPE	3downstream		
6	rs17287803	42039979	6.40E-07	p21.1	CCND3	intronic		
19	rs17706051	12345585	6.40E-07	p13.2	CTD-3105H18.5	3downstream		
14	rs1957173	45476666	6.40E-07	q21.2				
18	rs236035	6320209	6.40E-07	p11.31	L3MBTL4	intronic		
5	rs2879468	171739982	6.40E-07	q35.1	SH3PXD2B	intronic		
5	rs3777105	139230428	6.40E-07	q31.2	NRG2	intronic		
4	rs3810840	41816531	6.40E-07	p13	BEND4	intronic		

2	rs384180	6483994	6.40E-07	p25.2				
2	rs3893079	85192530	6.40E-07	p11.2				
18	rs401127	6305088	6.40E-07	p11.31	L3MBTL4	intronic		
17	rs4968211	7399786	6.40E-07	p13.1	TNFSF12_TNFSF1	intronic		
12	rs7135813	85718641	6.40E-07	q21.32	MGAT4C	intronic		
1	rs7537301	107998682	6.40E-07	p13.3	VAV3	intronic		
15	rs8042040	86729196	6.40E-07	q25.3				
17	rs8079544	7520777	6.40E-07	p13.1	TP53	intronic		
6	rs9355427	163699662	6.40E-07	q26				
13	rs9536258	52197146	6.40E-07	q14.3	LECT1	intronic		
1	rs9727625	230261992	6.40E-07	q42.2				
6	rs12197404	107416824	6.90E-07	q21				
3	rs2314042	155736835	6.90E-07	q25.2	RP11-656A15.1	non-coding intronic		
7	rs2526413	25060830	6.90E-07	p15.3				
4	rs282241	64889184	6.90E-07	q13.1	TECRL	intronic		
11	rs4567442	42610618	6.90E-07	p12				
6	rs9398330	113500683	6.90E-07	q21				
4	rs965016	64876557	6.90E-07	q13.1	TECRL	intronic		
4	rs11098654	123128865	7.48E-07	q27				
8	rs11984453	4491939	7.51E-07	p23.2	CSMD1	intronic		
3	rs2323206	74033842	7.79E-07	p13	RP11-20B7.1	non-coding intronic		
4	rs1355161	126487591	7.88E-07	q28.1	FAT4	intronic		
8	rs17468779	18835962	8.26E-07	p22	PSD3	intronic		
2	rs10490706	19355330	9.30E-07	p24.1	AC092594.1	non-coding intronic		
11	rs10891973	115793119	9.40E-07	q23.3				
11	rs11823029	115788726	9.40E-07	q23.3				
9	rs10970527	31781211	1.06E-06	p21.1				
13	rs12874087	67683706	1.29E-06	q21.33				
9	rs16928804	128033773	1.36E-06	q33.3				
6	rs853884	36576111	1.36E-06	p21.31	STK38	intronic		
20	rs17805193	59138980	1.41E-06	q13.33				
20	rs2427386	60794328	1.46E-06	q13.33				

2	rs7426302	231237153	1.46E-06	q37.1				
3	rs9869897	74035041	1.67E-06	p13	RP11-20B7.1	3downstream		
7	rs12539762	136175466	1.71E-06	q33	hsa-mir-490	non-coding intronic		
7	rs12539762	136175466	1.71E-06	q33	snoU13	3downstream		
16	rs17325839	56234402	1.72E-06	q21	GPR56	intronic		
23	rs6629669	23454170	1.85E-06	p22.11				
2	rs11883890	231230076	1.94E-06	q37.1				
2	rs214047	56344184	2.11E-06	p16.1	CCDC85A	intronic		
2	rs2869528	56312931	2.11E-06	p16.1	CCDC85A	intronic		
17	rs11655629	76524437	2.16E-06	q25.3	RPTOR	intronic		
1	rs2255340	230002773	2.21E-06	q42.2	DISC1	intronic		
1	rs2255340	230002773	2.21E-06	q42.2	TSNAX_DISC1	non-coding intronic		
9	rs880403	80616009	2.36E-06	q21.31				
9	rs10970502	31704812	2.37E-06	p21.1				
11	rs11219032	122527802	2.37E-06	q24.1	CLMP	intronic		
1	rs1555026	23759792	2.37E-06	p36.12	ID3	5upstream		
1	rs877518	23767741	2.37E-06	p36.12				
14	rs12897976	102962763	2.91E-06	q32.32	MARK3	intronic		
15	rs12915554	30810778	2.91E-06	q13.3	GREM1	3utr		
11	rs2040339	126346829	2.98E-06	q24.2	KIRREL3	intronic		
16	rs9928941	53302769	2.99E-06	q12.2				
1	rs11118119	216760495	3.03E-06	q41	C1orf143	intronic		
1	rs6691685	216753628	3.03E-06	q41	C1orf143	intronic		
2	rs472161	30783669	3.28E-06	p23.1				
22	rs17806754	32417553	3.42E-06	q12.3	LARGE	intronic		
18	rs11877750	68072361	3.46E-06	q22.3				
2	rs13028485	174213170	3.46E-06	q31.1				
11	rs7109274	59476992	3.46E-06	q12.1				
2	rs6707044	19354240	3.53E-06	p24.1	AC092594.1	non-coding intronic		
12	rs310830	75943724	3.67E-06	q21.2	E2F7	coding	770	S>S
14	rs765247	72261487	3.67E-06	q24.2	DPF3	intronic		
7	rs3094974	22451012	3.78E-06	p15.3	STEAP1B	intronic		

4	rs1465039	123187512	3.99E-06	q27				
4	rs1965582	123167389	3.99E-06	q27				
4	rs6554133	54391163	4.44E-06	q12	FIP1L1	intronic		
1	rs17103311	79746027	4.53E-06	p31.1				
1	rs3015045	79625651	4.53E-06	p31.1				
1	rs6665036	79638798	4.53E-06	p31.1				
1	rs6686663	79607654	4.53E-06	p31.1				
2	rs7423933	56269275	4.67E-06	p16.1	CCDC85A	intronic		
8	rs1445564	82044517	4.75E-06	q21.13	PAG1	3utr		
1	rs6667629	10447054	4.79E-06	p36.22	DFFA	intronic		
15	rs1520459	44730699	4.84E-06	q21.1				
1	rs16854967	229985969	4.84E-06	q42.2	DISC1	intronic		
1	rs16854967	229985969	4.84E-06	q42.2	TSNAX_DISC1	non-coding intronic		
5	rs585991	17246633	4.84E-06	p15.1	AC091878.1	non-coding intronic		
2	rs6432082	10331342	5.59E-06	p25.1				
11	rs10840415	10421488	5.68E-06	p15.4	AMPD3	intronic		
19	rs10412551	51605589	5.95E-06	q13.32	CCDC8	3utr		
16	rs1801255	56247306	5.98E-06	q21	GPR56	coding		
1	rs770713	5683616	6.01E-06	p36.31				
6	rs9387090	113062327	6.01E-06	q21				
13	rs7991325	113127674	6.98E-06	q34	ADPRHL1	intronic		
9	rs12216907	78225879	7.34E-06	q21.13	RP11-66C24.1	3downstream		
9	rs12216907	78225879	7.34E-06	q21.13	GCNT1	non-coding intronic		
7	rs10224816	9678184	7.36E-06	p21.3				
5	rs7444875	34520901	7.41E-06	p13.2				
22	rs4820778	26564347	7.43E-06	q12.1				
22	rs6005657	26565087	7.46E-06	q12.1				
13	rs17087794	70445442	7.64E-06	q21.33				
13	rs1475201	44347321	7.77E-06	q14.12				
3	rs7636993	36421322	7.77E-06	p22.3	STAC	intronic		
17	rs7216649	47522839	8.48E-06	q21.33	CA10	intronic		
16	rs12931123	86027384	8.63E-06	q24.2	ZCCHC14	intronic		

9	rs6479471	95084239	8.63E-06	q22.31	WNK2	intronic		
9	rs7022051	95042804	8.63E-06	q22.31	WNK2	intronic		
2	rs1437871	197540123	8.68E-06	q33.1	ANKRD44	3utr		
3	rs17195143	25042378	8.68E-06	p24.2	RNA5SP125	3downstream		
3	rs17195143	25042378	8.68E-06	p24.2	AC133680.1	non-coding intronic		
9	rs7875828	102499688	8.72E-06	q31.1				
7	rs1362361	29476911	8.75E-06	p14.3	CHN2	intronic		
15	rs1850348	44731046	8.93E-06	q21.1				
15	rs499567	44740689	8.93E-06	q21.1	RNU6-1014P	3downstream		
7	rs10263571	15421858	9.02E-06	p21.2	AGMO	intronic		
6	rs13192235	135388044	9.02E-06	q23.3	HBS1L	intronic		
7	rs4721438	15422074	9.02E-06	p21.2	AGMO	intronic		
4	rs1120767	126036233	9.07E-06	q28.1				
11	rs12804520	83368254	9.07E-06	q14.1	DLG2	intronic		
9	rs1360433	3739196	9.07E-06	p24.2				
11	rs2242312	129780556	9.07E-06	q24.3	ADAMTS8	3utr		
4	rs7686429	126034105	9.07E-06	q28.1				
12	rs1479449	23733078	9.63E-06	p12.1	SOX5	intronic		
2	rs11690021	239301967	9.71E-06	q37.3	AC113618.1	non-coding intronic		
4	rs13126054	159348081	9.75E-06	q32.1	TMEM144	intronic		
4	rs1928267	159362013	9.75E-06	q32.1	TMEM144	intronic		
16	rs9302496	13271756	9.76E-06	p13.12	U91319.1	non-coding intronic		
**Social**								
CHR	SNP	BP	P	Band	Genes	Role	aa Position	aa Change
23	rs1128863	154010056	2.65E-11	q28	MTCP1	non-coding intronic		
5	rs17097187	140692037	2.65E-11	q31.3	PCDHGA1	coding	534	A>A
23	rs237536	142819240	2.65E-11	q27.3				
23	rs5909473	18329671	2.65E-11	p22.13				
2	rs7426302	231237153	3.20E-11	q37.1				
2	rs12987781	203078111	1.75E-10	q33.2	BMPR2	intronic		
2	rs17199249	203134913	1.75E-10	q33.2	BMPR2	3utr		

2	rs2138380	202877469	1.75E-10	q33.1	NOP58	3downstream		
2	rs2350807	203004035	1.75E-10	q33.1	BMPR2	intronic		
6	rs11753940	64500431	3.58E-10	q12	EYS	intronic		
19	rs11882691	36774875	3.58E-10	q12	THEG5	5upstream		
4	rs16895825	17435354	3.58E-10	p15.31	NCAPG	intronic		
4	rs16895939	17488259	3.58E-10	p15.31	LCORL	intronic		
12	rs17041392	107206906	3.58E-10	q23.3	CMKLR1	3utr		
4	rs17051351	122562025	3.58E-10	q27				
7	rs17148818	24122943	3.58E-10	p15.3				
3	rs17526839	25580105	3.58E-10	p24.2	RARB	non-coding intronic		
7	rs3025153	24226447	3.58E-10	p15.3	AC004485.3	non-coding intronic		
2	rs3914642	45257987	3.58E-10	p21	LINC01121	non-coding intronic		
3	rs6782829	136438993	3.58E-10	q22.2	EPHB1	intronic		
4	rs7690457	17414477	3.58E-10	p15.31	DCAF16	coding	129	T>I
5	rs13169647	41468881	6.02E-10	p13.1	PLCXD3	intronic		
4	rs1355161	126487591	6.48E-10	q28.1	FAT4	intronic		
2	rs11883890	231230076	7.17E-10	q37.1				
11	rs12804520	83368254	8.27E-10	q14.1	DLG2	intronic		
11	rs2242312	129780556	8.27E-10	q24.3	ADAMTS8	3utr		
7	rs10263571	15421858	9.26E-10	p21.2	AGMO	intronic		
7	rs4721438	15422074	9.26E-10	p21.2	AGMO	intronic		
9	rs12216907	78225879	6.37E-09	q21.13	GCNT1	intronic		
10	rs772822	80304750	3.38E-08	q22.3				
18	rs7232149	10937468	4.98E-08	p11.21	PIEZO2	intronic		
18	rs9952954	10939093	4.98E-08	p11.21	PIEZO2	intronic		
13	rs1220582	23639868	5.01E-08	q12.12	SPATA13	intronic		
9	rs7025467	137199567	5.01E-08	q34.3				
2	rs11886946	21159092	1.09E-07	p24.1				
4	rs992253	89018302	1.09E-07	q22.1				
8	rs11984453	4491939	1.25E-07	p23.2	CSMD1	intronic		
2	rs7559811	231231148	1.72E-07	q37.1				
13	rs4145640	58077348	1.73E-07	q21.1				

6	rs1000308	26352559	1.77E-07	p22.2				
6	rs1341758	26308497	1.77E-07	p22.2	HIST1H2BF	3downstream/5upstream		
6	rs16891458	26342455	1.77E-07	p22.2	HIST1H1D	3utr		
6	rs6913828	26354643	1.77E-07	p22.2	HIST1H4G	3downstream		
6	rs989710	26314053	1.77E-07	p22.2	HIST1H4E	3downstream		
3	rs17564921	148372345	1.77E-07	q24	RP11-649A16.1	non-coding intronic		
16	rs8045917	17181496	2.03E-07	p12.3	XYLT1	intronic		
1	rs16841453	228161882	2.17E-07	q42.13				
3	rs10935663	148442795	2.22E-07	q24	RP11-649A16.1	non-coding intronic		
1	rs1002784	76597183	3.23E-07	p31.1	ST6GALNAC3	intronic		
5	rs10055858	89030846	3.23E-07	q14.3				
5	rs10063779	105861007	3.23E-07	q21.3				
7	rs10268591	119932159	3.23E-07	q31.31	KCND2	intronic		
6	rs10456309	24697541	3.23E-07	p22.3	KIAA0319	intronic		
5	rs10479370	105853569	3.23E-07	q21.3				
2	rs10490001	159847294	3.23E-07	q24.2	WDSUB1	intronic		
12	rs10507004	90798986	3.23E-07	q21.33	RP11-587P21.2	3downstream		
5	rs10520823	15819072	3.23E-07	p15.1	FBXL7	intronic		
5	rs10520825	15843676	3.23E-07	p15.1	FBXL7	intronic		
10	rs10887535	87652532	3.23E-07	q23.1	GRID1	intronic		
9	rs10979638	110784592	3.23E-07	q31.3	CTNNAL1	intronic		
9	rs11141680	88905129	3.23E-07	q21.33				
11	rs11217642	96195766	3.23E-07	q21				
16	rs11643685	61605744	3.23E-07	q21				
3	rs11706381	134557477	3.23E-07	q22.1	TMEM108	intronic		
3	rs11717365	134501970	3.23E-07	q22.1	TMEM108	intronic		
1	rs11805072	213051045	3.23E-07	q41				
16	rs11866237	9681936	3.23E-07	p13.2				
5	rs11950687	5742346	3.23E-07	p15.32				
7	rs11972142	47896705	3.23E-07	p12.3	PKD1L1	coding	879	T>A
9	rs12002251	134360990	3.23E-07	q34.13	C9orf171	intronic		
13	rs12018299	101947736	3.23E-07					

1	rs12043420	102471625	3.23E-07	p21.1	RP11-202K23.1	non-coding intronic		
1	rs12076827	157895588	3.23E-07	q23.2				
1	rs12083740	99497808	3.23E-07	p21.2				
10	rs1219510	123456933	3.23E-07	q26.13				
10	rs12261538	36057581	3.23E-07	p11.21				
9	rs12336476	89011803	3.23E-07	q21.33				
9	rs12337287	91945845	3.23E-07	q22.2				
3	rs12485617	60489702	3.23E-07	p14.2	FHIT	intronic		
1	rs12564848	172542992	3.23E-07	q25.1	RABGAP1L	intronic		
1	rs1275837	51734793	3.23E-07	p32.3	EPS15	intronic		
11	rs12793676	127364290	3.23E-07	q24.3				
20	rs13039715	31333951	3.23E-07	q11.21	BPIFB1	5upstream		
4	rs13126627	110542371	3.23E-07	q25	SEC24B-AS1	non-coding intronic		
7	rs13308082	67099810	3.23E-07	q11.22				
16	rs13330694	9698859	3.23E-07	p13.2				
16	rs13335480	61624033	3.23E-07	q21				
2	rs13394534	168216885	3.23E-07	q24.3				
2	rs13402616	201828012	3.23E-07	q33.1	CASP8	intronic		
1	rs1399437	18433075	3.23E-07	p36.13	IGSF21	intronic		
16	rs1461207	61895782	3.23E-07	q21				
8	rs1480693	14889670	3.23E-07	p22	SGCZ	intronic		
2	rs1527978	123421076	3.23E-07	q14.3				
13	rs1570885	23422626	3.23E-07	q12.12	ANKRD20A19P	5upstream		
12	rs1584091	39414082	3.23E-07	q12	CNTN1	intronic		
1	rs16847402	172981674	3.23E-07	q25.1	RABGAP1L	intronic		
1	rs16847563	173075477	3.23E-07	q25.1	RABGAP1L	intronic		
2	rs16859160	219223187	3.23E-07	q35	ZNF142	non-coding intronic		
10	rs1693680	123452831	3.23E-07	q26.13				
17	rs16948748	1404589	3.23E-07	p13.3	PITPNA	intronic		
16	rs16966127	9682827	3.23E-07	p13.2				
15	rs16974868	83450008	3.23E-07	q25.3	PDE8A	intronic		
12	rs1697664	129058768	3.23E-07	q24.33				

2	rs17009035	123448600	3.23E-07	q14.3				
13	rs17070878	47290134	3.23E-07	q14.2				
8	rs17077154	6338660	3.23E-07	p23.1	MCPH1	intronic		
14	rs17097056	98040463	3.23E-07	q32.2	RP11-1082A3.1	non-coding intronic		
16	rs17144475	7640614	3.23E-07	p13.3	RBFOX1	intronic		
7	rs17145007	67108027	3.23E-07	q11.22				
6	rs17209608	45164064	3.23E-07	p21.1	SUPT3H	intronic		
2	rs17269759	114269008	3.23E-07	q14.1				
18	rs17392470	5999234	3.23E-07	p11.31	L3MBTL4	intronic		
1	rs17489258	89446799	3.23E-07	p22.2				
12	rs17528736	54654785	3.23E-07	q13.2	CDK2	3downstream		
10	rs17598923	44025753	3.23E-07	q11.21				
4	rs17612737	111305670	3.23E-07	q25	ELOVL6	intronic		
17	rs17626856	48624506	3.23E-07	q22				
4	rs17627742	113458601	3.23E-07	q25	ALPK1	intronic		
15	rs17660734	51394095	3.23E-07	q21.3				
2	rs17763718	99403024	3.23E-07	q11.2	REV1	intronic		
1	rs1890881	172443546	3.23E-07	q25.1	RABGAP1L	intronic		
11	rs1938710	58290833	3.23E-07	q12.1				
1	rs2072758	172792540	3.23E-07	q25.1	RABGAP1L	intronic		
13	rs2166619	41542679	3.23E-07	q14.11	DGKH	intronic		
4	rs2343726	158099159	3.23E-07	q32.1	PDGFC	intronic		
5	rs2402181	15839586	3.23E-07	p15.1	FBXL7	intronic		
10	rs2483489	27566537	3.23E-07	p12.1	ACBD5	intronic		
3	rs2590446	189078365	3.23E-07	q27.3				
18	rs2621178	411412	3.23E-07	p11.32	COLEC12	intronic		
13	rs2793486	23632142	3.23E-07	q12.12	SPATA13	5upstream		
21	rs2831515	28407728	3.23E-07	q21.3	AL035610.1	non-coding intronic		
14	rs2899867	37680164	3.23E-07	q21.1	CTD-2058B24.2	non-coding intronic		
8	rs3020285	6321509	3.23E-07	p23.1	MCPH1	intronic		
5	rs37195	142337942	3.23E-07	q31.3	ARHGAP26	intronic		
17	rs3744453	42737869	3.23E-07	q21.32	ITGB3	intronic		

11	rs376813	8221633	3.23E-07	p15.4	LMO1	intronic		
5	rs392632	93979772	3.23E-07	q15	ANKRD32/KIAA08	5upstream		
21	rs406923	45362919	3.23E-07	q22.3	ADARB1	intronic		
14	rs4246989	20596530	3.23E-07	q11.2	RNASE8/NDRG2	3downstream		
2	rs4381764	201835108	3.23E-07	q33.1	CASP8	intronic		
4	rs4455441	80837831	3.23E-07	q21.21	RP11-452C8.1	3downstream		
1	rs4506448	229229266	3.23E-07	q42.2	FAM89A	intronic		
11	rs4528285	94025366	3.23E-07	q21	RP11-867G2.8	non-coding intronic		
8	rs4549773	14866331	3.23E-07	p22	SGCZ	intronic		
11	rs4575258	42689169	3.23E-07	p12				
4	rs4693893	89016789	3.23E-07	q22.1				
18	rs4799717	29581632	3.23E-07	q12.1	ASXL3	3downstream		
18	rs4941252	60033312	3.23E-07	q22.1	RP11_146N18.1	non-coding intronic		
8	rs532410	37157791	3.23E-07	p11.23				
1	rs582798	94753841	3.23E-07	p21.3	ABCD3	intronic		
23	rs5943189	110783611	3.23E-07	q23				
23	rs5953146	47771235	3.23E-07	p11.23	ZNF630	intronic		
20	rs6068063	50118387	3.23E-07	q13.2	ZFP64	intronic		
20	rs6111448	1718607	3.23E-07	p13				
5	rs640575	118164322	3.23E-07	q23.1				
5	rs6451105	34489338	3.23E-07	p13.2				
11	rs6590942	101362968	3.23E-07	q22.1	KIAA1377	coding	1077	S>N
2	rs6711579	32707160	3.23E-07	p22.3	TTC27	intronic		
2	rs6724815	71138848	3.23E-07	p13.3	AC007040.8	non-coding intronic		
2	rs6757356	231542832	3.23E-07	q37.1				
18	rs677592	64596256	3.23E-07	q22.1	CCDC102B	intronic		
3	rs6783318	163605873	3.23E-07	q26.1				
4	rs6857926	91615328	3.23E-07	q22.1	CCSER1	intronic		
6	rs6936924	34673137	3.23E-07	p21.31	C6orf106	intronic		
6	rs6938596	53124162	3.23E-07	p12.1				
6	rs6941744	53088262	3.23E-07	p12.1				
8	rs7010925	14882160	3.23E-07	p22	SGCZ	intronic		

9	rs7032191	89040606	3.23E-07	q21.33	BC093087	intronic		
9	rs7041667	88880438	3.23E-07	q21.33				
10	rs7076228	129660744	3.23E-07	q26.2	PTPRE	intronic		
1	rs712904	99496352	3.23E-07	p21.2				
15	rs7167692	83461188	3.23E-07	q25.3	PDE8A	intronic		
15	rs7182328	83451685	3.23E-07	q25.3	PDE8A	intronic		
16	rs7205559	80193831	3.23E-07	q23.2	CMIP	intronic		
17	rs7225354	76772657	3.23E-07	q25.3	AATK_AS1	3downstream		
17	rs7226359	76772601	3.23E-07	q25.3	AATK_AS1	3downstream		
4	rs729545	94120351	3.23E-07	q22.2	GRID2	intronic		
17	rs7342860	14473850	3.23E-07	p12				
6	rs7449883	94717168	3.23E-07	q16.1				
1	rs7531638	87967965	3.23E-07	p22.3				
11	rs765824	74580143	3.23E-07	q13.4	SLCO2B1	intronic		
4	rs768063	110839905	3.23E-07	q25	CASP6	intronic		
5	rs7715761	34445932	3.23E-07	p13.2				
6	rs7752412	18022783	3.23E-07	p22.3	KIF13A	intronic		
9	rs7855148	1506528	3.23E-07	p24.3				
9	rs7871216	36235759	3.23E-07	p13.3	GNE	intronic		
11	rs7932838	15634562	3.23E-07	p15.2	RP11-396O20.1	non-coding intronic		
13	rs7994396	42109344	3.23E-07	q14.11				
14	rs8014003	102230954	3.23E-07	q32.31	RCOR1	intronic		
5	rs8192207	6709161	3.23E-07	p15.31	SRD5A1	intronic		
1	rs819771	99500510	3.23E-07	p21.2	LPPR4	5upstream		
1	rs841166	245976637	3.23E-07	q44	RP11-634B7.4	non-coding		
9	rs869939	1043465	3.23E-07	p24.3	DMRT2	intronic		
13	rs9300818	102917124	3.23E-07	q33.1				
6	rs9488761	116158620	3.23E-07	q22.1				
17	rs951351	42719806	3.23E-07	q21.32	ITGB3	intronic		
13	rs9538062	57955513	3.23E-07	q21.1				
13	rs9598428	61669852	3.23E-07	q21.31				
13	rs9602747	84895132	3.23E-07	q31.1	LINC00351	non-coding intronic		

22	rs9613025	24563110	3.23E-07	q12.1	MYO18B	intronic		
2	rs9784032	219123706	3.23E-07	q35	USP37	intronic		
3	rs9819189	9226198	3.23E-07	p25.3	SRGAP3	intronic		
3	rs9829141	7333130	3.23E-07	p26.1	GRM7	intronic		
16	rs9932038	80172371	3.23E-07	q23.2	CMIP	intronic		
18	rs9948203	47828662	3.23E-07	q21.2				
18	rs10515975	53727976	3.26E-07	q21.31				
11	rs10734506	42616027	4.35E-07	p12				
5	rs11744289	53319086	4.35E-07	q11.2	ARL15	intronic		
8	rs11987866	14879967	4.35E-07	p22	SGCZ	intronic		
7	rs6946476	152651675	4.35E-07	q36.2				
17	rs11658255	34516027	4.51E-07	q12	PLXDC1	intronic		
6	rs4573107	128463455	4.51E-07	q22.33	PTPRK	intronic		
13	rs4770541	23468384	4.51E-07	q12.12	SPATA13	non-coding intronic		
14	rs11157351	42897456	4.66E-07	q21.2				
5	rs156030	131558816	4.67E-07	q31.1	P4HA2	intronic		
4	rs16871093	21078237	4.67E-07	p15.2	KCNIP4	intronic		
3	rs4955642	170466241	4.73E-07	q26.2	MECOM	intronic		
20	rs6092968	36802458	4.79E-07	q11.23				
2	rs11676424	239338075	4.90E-07	q37.3				
13	rs9600156	73170028	4.90E-07	q22.1	KLF12	intronic		
22	rs17806754	32417553	4.92E-07	q12.3	LARGE	intronic		
12	rs12812815	85758126	5.61E-07	q21.32	MGAT4C	5upstream		
21	rs7275508	25577166	6.45E-07	q21.2	AP000233.4	non-coding intronic		
9	rs6479471	95084239	7.13E-07	q22.31	WNK2	intronic		
9	rs7022051	95042804	7.13E-07	q22.31	WNK2	intronic		
4	rs17005129	82543901	7.41E-07	q21.21				
17	rs9897203	59960605	7.41E-07	q23.3	CEP95	intronic		
11	rs7123143	80683168	7.63E-07	q14.1				
6	rs522185	165552357	8.91E-07	q27				
20	rs17805193	59138980	9.35E-07	q13.33				
5	rs350042	40242561	9.49E-07	p13.1				

22	rs6005657	26565087	9.68E-07	q12.1				
7	rs10250704	150195351	1.05E-06	q36.1				
7	rs4725970	150189544	1.05E-06	q36.1	AOC1	3downstream		
10	rs16936610	80354231	1.14E-06	q22.3				
11	rs1879229	23938956	1.14E-06	p14.3				
20	rs3918278	44069061	1.14E-06	q13.12	MMP9	5upstream		
2	rs11903702	231229065	1.33E-06	q37.1				
2	rs12618382	231225449	1.33E-06	q37.1				
17	rs7216649	47522839	1.33E-06	q21.33	CA10	intronic		
2	rs10498133	222812549	1.34E-06	q36.1	PAX3	intronic		
8	rs16868879	103095400	1.34E-06	q22.3	NCALD	intronic		
5	rs16902290	34522293	1.34E-06	p13.2				
4	rs17005123	82534494	1.34E-06	q21.21				
6	rs17287803	42039979	1.34E-06	p21.1	CCND3	intronic		
14	rs1957173	45476666	1.34E-06	q21.2				
18	rs236035	6320209	1.34E-06	p11.31	L3MBTL4	intronic		
2	rs384180	6483994	1.34E-06	p25.2				
18	rs401127	6305088	1.34E-06	p11.31	L3MBTL4	intronic		
12	rs7135813	85718641	1.34E-06	q21.32	MGAT4C	intronic		
7	rs10234070	44504221	1.44E-06	p13				
4	rs11098654	123128865	1.54E-06	q27				
6	rs16870964	93937635	1.55E-06	q16.1				
14	rs765247	72261487	1.73E-06	q24.2	DPF3	intronic		
10	rs497204	80306297	1.89E-06	q22.3				
9	rs7049140	137201080	1.90E-06	q34.3				
9	rs3763646	110671791	2.46E-06	q31.3	IKBKAP	intronic		
9	rs3802431	110932446	2.46E-06	q31.3	FRRS1L	3utr		
9	rs7581	110819068	2.46E-06	q31.3	TMEM245	3utr		
23	rs6629669	23454170	2.48E-06	p22.11				
5	rs7444875	34520901	2.53E-06	p13.2				
13	rs1475201	44347321	2.60E-06	q14.12				
3	rs7636993	36421322	2.60E-06	p22.3	STAC	intronic		

13	rs7994080	89513893	2.60E-06	q31.3	LINC00559	non-coding intronic		
13	rs9583780	89516023	2.60E-06	q31.3	LINC00559	non-coding intronic		
7	rs4721433	15406633	3.20E-06	p21.2	AGMO	intronic		
8	rs2527724	5456598	3.49E-06	p23.2				
10	rs10444114	119650841	3.54E-06	q26.11				
12	rs11172790	57555007	3.54E-06	q14.1	LRIG3	intronic		
11	rs11220381	125529652	3.54E-06	q24.2				
19	rs11668338	7666624	3.54E-06	p13.2	FCER2	intronic		
4	rs11935585	30925362	3.54E-06	p15.1				
22	rs12160908	24258620	3.54E-06	q12.1	AL008721.1	3downstream		
7	rs13340481	109510205	3.54E-06	q31.1				
11	rs1488864	6298905	3.54E-06	p15.4	PRKCDBP	5upstream		
10	rs16932709	77349643	3.54E-06	q22.2	C10orf11	intronic		
23	rs16985316	8917394	3.54E-06	p22.31				
15	rs2242025	59810691	3.54E-06	q22.2	RP11-162I7.1	non-coding		
11	rs2682119	6307685	3.54E-06	p15.4				
23	rs3130866	72225071	3.54E-06	q13.2				
13	rs4773861	94704873	3.54E-06	q32.1	ABCC4	intronic		
23	rs5969912	151552128	3.54E-06	q28				
20	rs6099111	54370639	3.54E-06	q13.2	FAM210B	intronic		
13	rs7329514	94714801	3.54E-06	q32.1	ABCC4	intronic		
1	rs7544426	109977503	3.54E-06	p13.3	AMPD2	3downstream		
17	rs2676801	45048685	3.78E-06	q21.33	SPOP	intronic		
17	rs4794061	45131084	3.78E-06	q21.33				
11	rs10501635	86714986	4.08E-06	q14.2	TMEM135	3utr		
2	rs1437871	197540123	4.39E-06	q33.1	ANKRD44	3utr		
3	rs17195143	25042378	4.39E-06	p24.2	RNA5SP125/AC13	3downstream		
1	rs4373782	27722525	4.39E-06	p36.11				
2	rs6545561	56310240	4.39E-06	p16.1	CCDC85A	intronic		
2	rs6733298	56304360	4.39E-06	p16.1	CCDC85A	intronic		
2	rs6756156	56285098	4.39E-06	p16.1	CCDC85A	intronic		
8	rs11777289	131258480	4.44E-06	q24.21	ASAP1	intronic		

8	rs11778881	131360369	4.44E-06	q24.21	ASAP1	intronic		
8	rs4639572	131187840	4.44E-06	q24.21	ASAP1	intronic		
3	rs17038236	13515641	4.63E-06	p25.1	HDAC11	intronic		
9	rs10992681	95018086	4.65E-06	q22.31	WNK2	intronic		
17	rs2314640	9242675	4.71E-06	p13.1	STX8	non-coding intronic		
2	rs17039644	2377906	5.01E-06	p25.3				
3	rs17035408	36662904	5.11E-06	p22.2				
9	rs7857220	95064873	5.57E-06	q22.31	WNK2	intronic		
4	rs1120767	126036233	5.60E-06	q28.1				
9	rs1360433	3739196	5.60E-06	p24.2				
16	rs17248940	60324113	5.60E-06	q21	CDH8	intronic		
1	rs6701090	69273403	5.60E-06	p31.2				
4	rs7686429	126034105	5.60E-06	q28.1				
5	rs7720612	72490276	5.60E-06	q13.2	KIF13A	intronic		
6	rs13192235	135388044	5.68E-06	q23.3	HBS1L	intronic		
21	rs2242724	35663806	5.68E-06	q22.12	RUNX1	intronic		
11	rs963090	83356568	6.10E-06	q14.1	DLG2	intronic		
3	rs2116701	25416981	6.20E-06	p24.2	RARB	intronic		
17	rs3815269	11746991	6.30E-06	p12	DNAH9	intronic		
5	rs10070739	66580635	6.69E-06	q12.3				
3	rs12633552	73845979	6.69E-06	p13				
8	rs17468779	18835962	6.69E-06	p22	PSD3	intronic		
4	rs10002695	116780139	6.77E-06	q26				
4	rs4146409	116766549	6.77E-06	q26				
4	rs7673850	116805318	6.77E-06	q26				
4	rs7677317	116817988	6.77E-06	q26				
14	rs12897976	102962763	7.35E-06	q32.32	MARK3	intronic		
5	rs10940096	66586611	7.48E-06	q12.3				
7	rs10224816	9678184	7.68E-06	p21.3				
1	rs17044576	205701293	8.40E-06	q32.2	CR2	intronic		
11	rs290071	30962944	8.40E-06	p13	DCDC5	intronic		
16	rs7205322	18865263	8.40E-06	p12.3				

1	rs4660484	41141921	9.31E-06	p34.2				
7	rs12669535	150791535	9.46E-06	q36.1	RN7SL76P	3downstream		
16	rs9941146	12807987	9.67E-06	p13.12				
17	rs12951596	76494978	9.76E-06	q25.3	RPTOR	intronic		
**Perseverative Behaviors**								
CHR	SNP	BP	P	Band	Genes	Role	aa Position	aa Change
2	rs6745182	190533471	1.51E-08	q32.2	C2orf88	intronic		
7	rs4337997	22351942	1.40E-07	p15.3	RAPGEF5	intronic		
5	rs1870658	73612235	2.00E-07	q13.3				
5	rs471405	73613440	2.00E-07	q13.3				
6	rs1233386	29666169	5.28E-07	p22.1	OR2H2	3downstream		
5	rs10059061	95806928	1.23E-06	q15	CTD-2337A12.1	intronic		
13	rs9545030	78724117	1.42E-06	q31.1				
16	rs17261378	81231342	1.58E-06	q23.3	CDH13	intronic		
9	rs3780457	99962228	1.58E-06	q22.33	CORO2A	intronic		
4	rs2871491	186937857	1.81E-06	q35.1	SORBS2	intronic		
17	rs9894565	17047909	2.48E-06	p11.2	PLD6/RP11-45M2	intronic		
5	rs149473	123648735	3.96E-06	q23.2				
13	rs7339418	78718127	3.96E-06	q31.1				
13	rs7983960	78734451	3.96E-06	q31.1				
13	rs9545039	78740745	3.96E-06	q31.1				
6	rs12529308	40797708	4.37E-06	p21.1				
6	rs7756298	40808750	4.37E-06	p21.1				
3	rs9310756	24767428	5.17E-06	p24.2	AC133680.1	intronic		
12	rs2708079	41202340	6.77E-06	q12	PRICKLE1	intronic		
7	rs12333620	22324730	6.90E-06	p15.3	RAPGEF5	intronic		
7	rs1568571	22341449	6.90E-06	p15.3	RAPGEF5	intronic		
13	rs12874523	105487642	7.33E-06	q33.2				
9	rs7042145	8394095	8.14E-06	p24.1	PTPRD	intronic		
8	rs11784372	121589419	8.48E-06	q24.12	MTBP	intronic		
3	rs6438175	115134904	8.48E-06	q13.31	GRAMD1C	intronic		

6	rs6912147	22371817	8.48E-06	p22.3	RP3-404K8.2	intronic		
