# Supplementary material for: ASD Phenotype—Genotype Associations in Concordant and Discordant Monozygotic and Dizygotic Twins Stratified by Severity of Autistic Traits
Source: Int J Mol Sci. 2019 Aug 3;20(15):3804. doi: 10.3390/ijms20153804 (PMC6696087; doi:10.3390/ijms20153804)
Supplement: Supplementary file 1 [file ijms-20-03804-s001.zip › Supplementary Materials/Table S7. Case-control by quartile analysis of cMZ twins.pdf]

Summary of case-control analyses by quartiles of cMZ twins

Language

Q1 (N = 13)	CHR	SNP	BP	A1	F_A	F_U	A2	CHISQ	OR	UNADJ	BONF	FDR_BH	Band	Alleles	Gene(s)	Role
	6	rs7767901	9014658	C	0.03846	0.2117	T	4.666	0.1489	3.08E-02	1.23E-01	6.39E-02	6p24.3	C/T		
	6	rs6597379	9000801	T	0.03846	0.21	C	4.601	0.1505	3.20E-02	1.28E-01	6.39E-02	6p24.3	C/T		

Q4 (N = 18)	CHR	SNP	BP	A1	F_A	F_U	A2	CHISQ	OR	UNADJ	BONF	FDR_BH	Band	Alleles	Gene(s)	Role
	8	rs4481588	35609723	T	0.25	0.08764	C	11.64	3.47	6.46E-04	2.59E-03	2.59E-03	8p12	C/T	UNC5D	Intron
	5	rs2546481	1.15E+08	T	0.3056	0.1445	C	7.448	2.605	6.35E-03	2.54E-02	1.27E-02	5q22.3	C/T		
	6	rs6597379	9000801	T	0.3611	0.21	C	4.902	2.127	2.68E-02	1.07E-01	2.91E-02	6p24.3	C/T		
	6	rs7767901	9014658	C	0.3611	0.2117	T	4.762	2.105	2.91E-02	1.16E-01	2.91E-02	6p24.3	C/T		

Nonverbal

Q1 (N = 11)	CHR	SNP	BP	A1	F_A	F_U	A2	CHISQ	OR	UNADJ	BONF	FDR_BH	Band	Alleles	Gene(s)	Role
	8	rs359782	47302891	A	0.65	0.2483	G	17.14	5.623	3.47E-05	4.17E-04	4.17E-04	8q11.1	A/G		
	14	rs7145220	34193684	G	0.75	0.3704	A	12.28	5.099	4.57E-04	5.48E-03	2.74E-03	14q13.1	A/G		
	4	rs1313228	20972739	G	0.8182	0.4608	A	11.25	5.265	7.96E-04	9.55E-03	3.18E-03	4p15.2	A/G	KCNIP4	Intron
	22	rs2073454	20847479	A	0.75	0.4123	C	9.361	4.276	2.22E-03	2.66E-02	4.21E-03	22q11.22	A/C		
	5	rs2216904	51769238	T	0.7273	0.409	C	9.168	3.853	2.46E-03	2.96E-02	4.21E-03	5q11.2	C/T		
	7	rs1023568	26875121	C	0.3182	0.1133	T	9.087	3.652	2.58E-03	3.09E-02	4.21E-03	7p15.2	C/T	SKAP2	Promoter
	4	rs893929	1.44E+08	A	0.1364	0.4559	G	9.018	0.1885	2.67E-03	3.21E-02	4.21E-03	4q31.21	A/G		
	13	rs1886215	41847359	T	0.7273	0.4128	C	8.928	3.794	2.81E-03	3.37E-02	4.21E-03	13q14.11	C/T		
	1	rs1092530	2.35E+08	T	0.3182	0.1189	C	8.244	3.457	4.09E-03	4.91E-02	4.99E-03	1q43	C/T	RYR2	Intron
	3	rs1554125	58252541	A	0.5455	0.2724	C	8.215	3.205	4.16E-03	4.99E-02	4.99E-03	3p14.3	A/C	ABHD6	Intron
	13	rs2221406	88972527	A	0.4091	0.1783	G	7.928	3.191	4.87E-03	5.84E-02	5.31E-03	13q31.3	A/G		
	4	rs1768636	1.44E+08	A	0.1364	0.4017	G	6.423	0.2351	1.13E-02	1.35E-01	1.13E-02	4q31.21	A/G		

Q4 (N = 18)	CHR	SNP	BP	A1	F_A	F_U	A2	CHISQ	OR	UNADJ	BONF	FDR_BH	Band	Alleles	Gene(s)	Role
	4	rs1768636	1.44E+08	A	0.6667	0.4017	G	10.42	2.979	1.25E-03	1.50E-02	1.50E-02	4q31.21	A/G		
	22	rs2073454	20847479	A	0.1667	0.4123	C	8.911	0.285	2.84E-03	3.40E-02	1.70E-02	22q11.22	A/C		
	4	rs893929	1.44E+08	A	0.6667	0.4559	G	6.398	2.387	1.14E-02	1.37E-01	3.48E-02	4q31.21	A/G		
	14	rs7145220	34193684	G	0.1667	0.3704	A	6.372	0.3399	1.16E-02	1.39E-01	3.48E-02	14q13.1	A/G		
	13	rs2221406	88972527	A	0.02778	0.1783	G	5.552	0.1317	1.85E-02	2.22E-01	3.85E-02	13q31.3	A/G		
	8	rs359782	47302891	A	0.08333	0.2483	G	5.226	0.2752	2.23E-02	2.67E-01	3.85E-02	8q11.1	A/G		
	1	rs1092530	2.35E+08	T	0	0.1189	C	4.855	0	2.76E-02	3.31E-01	3.85E-02	1q43	C/T	RYR2	Intron
	4	rs1313228	20972739	G	0.2778	0.4608	A	4.821	0.45	2.81E-02	3.38E-01	3.85E-02	4p15.2	A/G	KCNIP4	Intron
	3	rs1554125	58252541	A	0.1111	0.2724	C	4.704	0.3339	3.01E-02	3.61E-01	3.85E-02	3p14.3	A/C	ABHD6	Intron
	7	rs1023568	26875121	C	0	0.1133	T	4.596	0	3.21E-02	3.85E-01	3.85E-02	7p15.2	C/T	SKAP2	Promoter
	13	rs1886215	41847359	T	0.25	0.4128	C	3.909	0.4742	4.80E-02	5.76E-01	5.24E-02	13q14.11	C/T		
	5	rs2216904	51769238	T	0.25	0.409	C	3.741	0.4817	5.31E-02	6.37E-01	5.31E-02	5q11.2	C/T		

Play

Q1 (N = 14)	CHR	SNP	BP	A1	F_A	F_U	A2	CHISQ	OR	UNADJ	BONF	FDR_BH	Band	Alleles	Gene(s)	Role
in Q1	1	rs497576	2.38E+08	T	0.1538	0.4754	C	10.73	0.2006	1.06E-03	5.28E-03	5.28E-03	1q43	C/T	CHRM3	Intron
	4	rs1313091	1.74E+08	T	0.4643	0.2463	C	7.103	2.652	7.70E-03	3.85E-02	1.10E-02	4q34.1	C/T	GALNTL6	Intron
	3	rs347134	32414789	C	0.1071	0.3456	T	7.012	0.2272	8.10E-03	4.05E-02	1.10E-02	3p22.3	C/T	CMTM7	Intron
	2	rs1866472	1.29E+08	T	0.1071	0.3426	C	6.863	0.2303	8.80E-03	4.40E-02	1.10E-02	2q14.3	C/T		
	5	rs357519	87546610	G	0	0.1385	T	4.177	0	4.10E-02	2.05E-01	4.10E-02	5q14.3	G/T	TMEM161	Intron

Q4 (N = 14)	CHR	SNP	BP	A1	F_A	F_U	A2	CHISQ	OR	UNADJ	BONF	FDR_BH	Band	Alleles	Gene(s)	Role
in Q4	5	rs357519	87546610	G	0.4286	0.1385	T	19.43	4.665	1.04E-05	5.21E-05	5.21E-05	5q14.3	G/T	TMEM161	Intron
	2	rs1866472	1.29E+08	T	0.625	0.3426	C	8.437	3.198	3.68E-03	1.84E-02	9.19E-03	2q14.3	C/T		
	4	rs1313091	1.74E+08	T	0.03571	0.2463	C	6.672	0.1133	9.79E-03	4.90E-02	1.23E-02	4q34.1	C/T	GALNTL6	Intron
	1	rs497576	2.38E+08	T	0.7143	0.4754	C	6.367	2.758	1.16E-02	5.81E-02	1.23E-02	1q43	C/T	CHRM3	Intron
	3	rs347134	32414789	C	0.5714	0.3456	T	6.268	2.525	1.23E-02	6.15E-02	1.23E-02	3p22.3	C/T	CMTM7	Intron

Social

Q1 (N = 15)	CHR	SNP	BP	A1	F_A	F_U	A2	CHISQ	OR	UNADJ	BONF	FDR_BH	Band	Alleles	Genes	Role
	6	rs1115371	1.18E+08	T	0.6333	0.2313	C	26.91	5.741	2.13E-07	1.51E-05	1.51E-05				
	3	rs9682960	11064345	C	0.3667	0.1142	T	18.57	4.491	1.64E-05	1.16E-03	3.29E-04	p25.3			
	8	rs7827943	73568186	A	0.5667	0.2327	G	18.52	4.313	1.68E-05	1.20E-03	3.29E-04	q13.3	A/G		
	6	rs3004002	77426060	T	0.7667	0.3847	C	18.34	5.255	1.85E-05	1.32E-03	3.29E-04	q14.1	G|A	RP11-354K	5upstream
	8	rs1010002	73553339	A	0.4667	0.1775	C	16.92	4.054	3.89E-05	2.76E-03	5.52E-04	q13.3	A/C		
	8	rs359782	47302891	A	0.5769	0.2482	G	14.9	4.131	1.13E-04	8.04E-03	1.34E-03	q11.1			
	3	rs4365667	1.67E+08	A	0.5667	0.2715	C	13.08	3.509	2.99E-04	2.12E-02	2.72E-03	q26.1			
	8	rs9650442	47224322	T	0.5333	0.2493	C	12.78	3.441	3.51E-04	2.49E-02	2.72E-03	q11.1			
	8	rs7836486	47062007	A	0.5333	0.2524	C	12.42	3.386	4.25E-04	3.02E-02	2.72E-03	q11.1	A/C		
	12	rs8186746	36633905	T	0.7667	0.4461	G	12.38	4.08	4.33E-04	3.08E-02	2.72E-03	q12			
	8	rs1009982	47711911	G	0.5333	0.254	A	12.22	3.357	4.73E-04	3.36E-02	2.72E-03	q11.1			
	8	rs7293777	47261663	T	0.5333	0.2547	C	12.13	3.344	4.97E-04	3.53E-02	2.72E-03	q11.1			
	8	rs990760	47859016	T	0.5333	0.257	C	11.88	3.305	5.69E-04	4.04E-02	2.72E-03	q11.1	A|G	MTND1P7,	non-coding intronic
	8	rs1916188	47915603	A	0.5333	0.2581	C	11.74	3.285	6.10E-04	4.33E-02	2.72E-03	q11.1			
	8	rs1113614	47240116	G	0.5333	0.2581	A	11.74	3.284	6.12E-04	4.35E-02	2.72E-03	q11.1	A/G		
	18	rs1768373	52800291	A	0.5	0.2338	G	11.74	3.277	6.12E-04	4.35E-02	2.72E-03	q21.31	G|A	WDR7	intronic
	8	rs359807	47309786	T	0.5333	0.2595	C	11.58	3.261	6.66E-04	4.73E-02	2.77E-03	q11.1	A/G		
	8	rs2354491	47901302	C	0.5333	0.2604	T	11.49	3.246	7.01E-04	4.98E-02	2.77E-03	q11.1	G|A	RP11-101E	3downstream

	CHR	SNP	BP	A1	F_A	F_U	A2	CHISQ	OR	UNADJ	BONF	FDR_BH	Band	Alleles	Gene(s)	Role
	8	rs4873076	47255301	C	0.5333	0.2633	T	11.17	3.198	8.32E-04	5.91E-02	3.03E-03	q11.1	C/T		
	1	rs4908488	7947179	C	0.4	0.1699	T	11.12	3.257	8.54E-04	6.06E-02	3.03E-03	p36.23	T|C	PARK7	intronic
	13	rs1417216	65202004	A	0.2	0.05763	G	10.97	4.088	9.25E-04	6.56E-02	3.09E-03	q21.32			
	8	rs7293734	47457400	T	0.5333	0.2662	C	10.85	3.15	9.88E-04	7.02E-02	3.09E-03	q11.1			
	12	rs1230693	36528296	T	0.7667	0.4673	C	10.73	3.745	1.06E-03	7.49E-02	3.09E-03	q12			
	8	rs1199170	77674728	A	0.4	0.173	G	10.68	3.188	1.09E-03	7.71E-02	3.09E-03	q21.11	G|A	ZFHX4-AS1	non-coding intronic
	10	rs3802739	1.21E+08	C	0.6667	0.3764	T	10.67	3.313	1.09E-03	7.71E-02	3.09E-03	q26.11	T|C	FAM45A	intronic
	8	rs4535748	47582366	T	0.5333	0.2688	C	10.58	3.109	1.15E-03	8.13E-02	3.13E-03	q11.1	G|A	HSPA8P13	3downstream
	6	rs9379030	6340921	G	0.4333	0.1972	A	10.44	3.113	1.23E-03	8.74E-02	3.20E-03	p25.1	A|G	LY86_AS1	non-coding intronic
	12	rs7966266	36912405	A	0.7667	0.4718	G	10.4	3.679	1.26E-03	8.95E-02	3.20E-03	q12			
	10	rs6602032	3159771	G	0.6	0.3249	A	10.26	3.117	1.36E-03	9.65E-02	3.30E-03	p15.2	G|A	PFKP	intronic
	10	rs915272	1.21E+08	T	0.6667	0.382	C	10.21	3.235	1.39E-03	9.89E-02	3.30E-03	q26.11	T|C	FAM45B	non-coding intronic
	1	rs2140682	34596209	C	0.1667	0.4549	T	9.995	0.2397	1.57E-03	1.11E-01	3.45E-03	p34.3			
	6	rs9374710	1.19E+08	A	0.06667	0.3391	C	9.9	0.1392	1.65E-03	1.17E-01	3.45E-03	q22.2	C|A	SLC35F1	intronic
	6	rs4245496	1.18E+08	T	0.06667	0.3387	C	9.877	0.1394	1.67E-03	1.19E-01	3.45E-03	q22.2	C|T	SLC35F1	intronic
	3	rs1332011	1.24E+08	C	0.7	0.4163	T	9.862	3.271	1.69E-03	1.20E-01	3.45E-03	q21.1	T|C	FAM162A	intronic
	1	rs2049420	34596147	C	0.1333	0.4165	T	9.847	0.2156	1.70E-03	1.21E-01	3.45E-03	p34.3			
	4	rs7664705	1.79E+08	T	0.06667	0.335	C	9.663	0.1418	1.88E-03	1.34E-01	3.71E-03	q34.3	C|T	RP11-130F	non-coding intronic
	8	rs359813	47339255	A	0.5	0.2472	G	9.516	3.046	2.04E-03	1.45E-01	3.91E-03	q11.1	C/T		
	5	rs6556348	1.58E+08	T	0.7	0.4231	C	9.357	3.182	2.22E-03	1.58E-01	4.15E-03	q33.3	C|T	RP11-542A	non-coding intronic
	8	rs1113615	47292439	T	0.5	0.2593	G	8.966	2.857	2.75E-03	1.95E-01	5.01E-03	q11.1	T|G	AC113134.	3downstream
	20	rs2209339	12607029	A	0.2	0.471	C	8.793	0.2808	3.02E-03	2.15E-01	5.37E-03	p12.1			
	5	rs253365	16850415	C	0.7	0.4372	T	8.359	3.003	3.84E-03	2.73E-01	6.62E-03	p15.1	C|T	MYO10	intronic
	7	rs1048690	88621188	T	0.06667	0.3109	C	8.321	0.1584	3.92E-03	2.78E-01	6.62E-03	q21.13	C|T	ZNF804B	intronic
	12	rs1601746	36912462	C	0.2333	0.4891	A	7.806	0.3179	5.21E-03	3.70E-01	8.60E-03	q12			
	10	rs4880592	3163581	G	0.1333	0.3794	A	7.677	0.2517	5.59E-03	3.97E-01	9.02E-03	p15.2	A|G	PFKP	intronic
	7	rs7787455	88588409	A	0.06667	0.2914	G	7.312	0.1737	6.85E-03	4.86E-01	1.08E-02	q21.13	A|G	ZNF804B	intronic
	8	rs1205636	47588382	A	0.5	0.2676	G	7.108	2.737	7.68E-03	5.45E-01	1.19E-02	q11.1			
	8	rs406800	88604177	G	0.7333	0.493	A	6.888	2.828	8.68E-03	6.16E-01	1.31E-02	q21.3	C|T	CNBD1	intronic
	9	rs1160246	1.19E+08	T	0.6333	0.4006	C	6.719	2.585	9.54E-03	6.77E-01	1.41E-02	q33.1	C|T	ASTN2	intronic
	9	rs871120	1.19E+08	A	0.6333	0.4025	G	6.598	2.564	1.02E-02	7.25E-01	1.48E-02	q33.1	C|T	ASTN2	intronic
	12	rs2138077	1.27E+08	A	0.1667	0.393	G	6.412	0.3089	1.13E-02	8.05E-01	1.61E-02	q24.32			
	2	rs6743414	49277575	A	0	0.1748	G	6.345	0	1.18E-02	8.36E-01	1.64E-02	p16.3			
	7	rs2189061	88577308	T	0.03333	0.2224	G	6.185	0.1206	1.29E-02	9.15E-01	1.74E-02	q21.13	T|G	ZNF804B	intronic
	13	rs9635033	79956028	T	0.2	0.4248	G	6.17	0.3385	1.30E-02	9.23E-01	1.74E-02	q31.1			
	5	rs7724820	1.58E+08	A	0.6333	0.4113	G	6.064	2.472	1.38E-02	9.80E-01	1.81E-02	q33.3	G|A	LOC10192	non-coding intronic
	13	rs9532449	39369698	T	0.1333	0.3443	C	5.888	0.293	1.52E-02	1.00E+00	1.97E-02	q14.11			
	17	rs1165610	73754653	T	0.1667	0.3796	C	5.747	0.3269	1.65E-02	1.00E+00	2.09E-02	q25.3	C|T	THA1P	3downstream
	11	rs479844	65308533	T	0.2667	0.4821	C	5.545	0.3906	1.85E-02	1.00E+00	2.31E-02	q13.1			
	18	rs1940979	23511166	T	0.1667	0.364	C	5.024	0.3494	2.50E-02	1.00E+00	3.06E-02	q12.1			
	13	rs2221406	88972527	A	0.3333	0.1782	G	4.879	2.306	2.72E-02	1.00E+00	3.23E-02	q31.3			
	5	rs1952651	1.58E+08	G	0.6333	0.4329	T	4.875	2.263	2.73E-02	1.00E+00	3.23E-02	q33.3	A|C	LOC10192	non-coding intronic
	7	rs7802386	88591218	A	0	0.1391	C	4.844	0	2.78E-02	1.00E+00	3.23E-02	q21.13	A|C	ZNF804B	intronic
	21	rs2822892	15044008	G	0.5667	0.3733	A	4.762	2.196	2.91E-02	1.00E+00	3.32E-02	q11.2	A|G	AF127936.	non-coding intronic
	3	rs1170873	72008678	C	0.4	0.2315	T	4.741	2.213	2.95E-02	1.00E+00	3.32E-02	p13			
	2	rs6433615	1.77E+08	G	0.3	0.158	A	4.501	2.284	3.39E-02	1.00E+00	3.76E-02	q31.1	A|G	AC092162.	non-coding intronic
	8	rs6473217	81360413	T	0.06667	0.2268	C	4.372	0.2436	3.65E-02	1.00E+00	3.99E-02	q21.13	T|C	RP11-941H	non-coding intronic
	5	rs4704936	1.58E+08	G	0.6333	0.4527	T	3.923	2.088	4.76E-02	1.00E+00	5.12E-02	q33.3	T|G	LOC10192	non-coding intronic
	3	rs1554125	58252541	A	0.4333	0.2723	C	3.893	2.044	4.85E-02	1.00E+00	5.14E-02	p14.3	A|C	ABHD6	intronic
	8	rs4872963	47950630	T	0.5333	0.3622	C	3.776	2.013	5.20E-02	1.00E+00	5.43E-02	q11.1			
	3	rs6445971	58289364	G	0.4333	0.281	T	3.419	1.957	6.44E-02	1.00E+00	6.63E-02	p14.3	G|T	RP11-80H1	5upstream
	7	rs9986865	36807127	C	0.3	0.1783	T	3.005	1.975	8.30E-02	1.00E+00	8.42E-02	p14.2	C/T		

Q4 (N = 14)	CHR	SNP	BP	A1	F_A	F_U	A2	CHISQ	OR	UNADJ	BONF	FDR_BH	Band	Alleles	Gene(s)	Role
	8	rs6473217	81360413	T	0.5714	0.2268	C	18.75	4.546	1.49E-05	1.06E-03	1.06E-03	q21.13	T|C	RP11-941H	non-coding intronic
	7	rs7802386	88591218	A	0.3929	0.1391	C	14.83	4.004	1.17E-04	8.33E-03	2.95E-03	q21.13	A|C	ZNF804B	intronic
	18	rs1940979	23511166	T	0.7143	0.364	C	14.72	4.368	1.25E-04	8.86E-03	2.95E-03	q12.1			
	13	rs9532449	39369698	T	0.6429	0.3443	C	10.97	3.428	9.27E-04	6.58E-02	1.59E-02	q14.11			
	10	rs4880592	3163581	G	0.6786	0.3794	A	10.57	3.454	1.15E-03	8.17E-02	1.59E-02	p15.2	A|G	PFKP	intronic
	9	rs871120	1.19E+08	A	0.1071	0.4025	G	10.11	0.1781	1.47E-03	1.05E-01	1.59E-02	q33.1	C|T	ASTN2	intronic
	9	rs1160246	1.19E+08	T	0.1071	0.4006	C	9.997	0.1796	1.57E-03	1.11E-01	1.59E-02	q33.1	C|T	ASTN2	intronic
	7	rs2189061	88577308	T	0.4643	0.2224	G	9.379	3.031	2.20E-03	1.56E-01	1.95E-02	q21.13	T|G	ZNF804B	intronic
	20	rs2209339	12607029	A	0.75	0.471	C	8.692	3.369	3.20E-03	2.27E-01	2.52E-02	p12.1			
	21	rs2822892	15044008	G	0.1071	0.3733	A	8.441	0.2015	3.67E-03	2.60E-01	2.58E-02	q11.2	A|G	AF127936.	non-coding intronic
	7	rs7787455	88588409	A	0.5357	0.2914	G	8.027	2.806	4.61E-03	3.27E-01	2.58E-02	q21.13	A|G	ZNF804B	intronic
	3	rs1554125	58252541	A	0.03571	0.2723	C	7.888	0.09898	4.98E-03	3.53E-01	2.58E-02	p14.3	A|C	ABHD6	intronic
	8	rs4872963	47950630	T	0.1071	0.3622	C	7.853	0.2113	5.07E-03	3.60E-01	2.58E-02	q11.1			
	13	rs9635033	79956028	T	0.7083	0.4248	G	7.847	3.288	5.09E-03	3.61E-01	2.58E-02	q31.1			
	5	rs6556348	1.58E+08	T	0.1786	0.4231	C	6.826	0.2964	8.99E-03	6.38E-01	4.04E-02	q33.3	C|T	RP11-542A	non-coding intronic
	10	rs915272	1.21E+08	T	0.1429	0.382	C	6.755	0.2696	9.35E-03	6.64E-01	4.04E-02	q26.11	T|C	FAM45B	non-coding intronic
	7	rs1048690	88621188	T	0.5357	0.3109	C	6.556	2.558	1.05E-02	7.42E-01	4.04E-02	q21.13	C|T	ZNF804B	intronic
	10	rs3802739	1.21E+08	C	0.125	0.3764	T	6.439	0.2366	1.12E-02	7.93E-01	4.04E-02	q26.11	T|C	FAM45A	intronic
	5	rs4704936	1.58E+08	G	0.2143	0.4527	T	6.392	0.3297	1.15E-02	8.14E-01	4.04E-02	q33.3	T|G	LOC10192	non-coding intronic
	7	rs7795594	36794360	G	0	0.1838	A	6.299	0	1.21E-02	8.58E-01	4.04E-02	p14.2			
	8	rs7836486	47062007	A	0.03846	0.2524	C	6.29	0.1185	1.21E-02	8.62E-01	4.04E-02	q11.1			
	5	rs7724820	1.58E+08	A	0.1786	0.4113	G	6.233	0.3112	1.25E-02	8.90E-01	4.04E-02	q33.3	G|A	LOC10192	non-coding intronic

CHR	SNP	BP	A1	F_A	F_U	A2	CHISQ	OR	UNADJ	BONF	FDR_BH	Band	Alleles	Gene(s)	Role
7	rs9986865	36807127	C	0	0.1783	T	6.068	0	1.38E-02	9.77E-01	4.04E-02	p14.2			
3	rs6445971	58289364	G	0.07143	0.281	T	6.066	0.1969	1.38E-02	9.78E-01	4.04E-02	p14.3	G|T	RP11-80H1	5upstream
11	rs479844	65308533	T	0.7143	0.4821	C	6.008	2.685	1.42E-02	1.00E+00	4.04E-02	q13.1			
8	rs1199170	77674728	A	0	0.173	G	5.849	0	1.56E-02	1.00E+00	4.26E-02	q21.11	G|A	ZFHX4-AS1	non-coding intronic
5	rs1952651	1.58E+08	G	0.2143	0.4329	T	5.424	0.3572	1.99E-02	1.00E+00	5.05E-02	q33.3	A|C	LOC10192	non-coding intronic
12	rs2138077	1.27E+08	A	0.6071	0.393	G	5.344	2.387	2.08E-02	1.00E+00	5.05E-02	q24.32			
8	rs4873076	47255301	C	0.07143	0.2633	T	5.296	0.2153	2.14E-02	1.00E+00	5.05E-02	q11.1			
2	rs6433615	1.77E+08	G	0	0.158	A	5.248	0	2.20E-02	1.00E+00	5.05E-02	q31.1	A|G	AC092162.	non-coding intronic
8	rs1010002	73553339	A	0	0.1775	C	5.174	0	2.29E-02	1.00E+00	5.05E-02	q13.3			
8	rs359807	47309786	T	0.07143	0.2595	C	5.14	0.2195	2.34E-02	1.00E+00	5.05E-02	q11.1			
8	rs1113615	47292439	T	0.07143	0.2593	G	5.128	0.2198	2.35E-02	1.00E+00	5.05E-02	q11.1	T|G	AC113134.	3downstream
8	rs1113614	47240116	G	0.07143	0.2581	A	5.082	0.2211	2.42E-02	1.00E+00	5.05E-02	q11.1			
6	rs4245496	1.18E+08	T	0.5357	0.3387	C	4.814	2.252	2.82E-02	1.00E+00	5.73E-02	q22.2	C|T	SLC35F1	intronic
8	rs359782	47302891	A	0.07143	0.2482	G	4.674	0.233	3.06E-02	1.00E+00	6.04E-02	q11.1			
6	rs9379030	6340921	G	0.03571	0.1972	A	4.602	0.1508	3.19E-02	1.00E+00	6.05E-02	p25.1	A|G	LY86_AS1	non-coding intronic
6	rs9374710	1.19E+08	A	0.5385	0.3391	C	4.578	2.274	3.24E-02	1.00E+00	6.05E-02	q22.2	C|A	SLC35F1	intronic
17	rs1165610	73754653	T	0.5714	0.3796	C	4.346	2.179	3.71E-02	1.00E+00	6.75E-02	q25.3	C|T	THA1P	3downstream
10	rs6602032	3159771	G	0.1429	0.3249	A	4.213	0.3463	4.01E-02	1.00E+00	7.05E-02	p15.2	G|A	PFKP	intronic
1	rs2049420	34596147	C	0.6071	0.4165	T	4.162	2.165	4.13E-02	1.00E+00	7.05E-02	p34.3			
2	rs6743414	49277575	A	0.3214	0.1748	G	4.137	2.237	4.20E-02	1.00E+00	7.05E-02	p16.3			
18	rs1768373	52800291	A	0.07143	0.2338	G	4.109	0.2521	4.27E-02	1.00E+00	7.05E-02	q21.31	G|A	WDR7	intronic
8	rs7827943	73568186	A	0.07143	0.2327	G	4.065	0.2537	4.38E-02	1.00E+00	7.06E-02	q13.3			
3	rs1170873	72008678	C	0.07143	0.2315	T	4.022	0.2553	4.49E-02	1.00E+00	7.08E-02	p13			
13	rs2221406	88972527	A	0.03571	0.1782	G	3.874	0.1708	4.91E-02	1.00E+00	7.57E-02	q31.3			
8	rs4535748	47582366	T	0.1071	0.2688	C	3.709	0.3264	5.41E-02	1.00E+00	7.74E-02	q11.1	G|A	HSPA8P13	3downstream
8	rs7293777	47261663	T	0.08333	0.2547	C	3.703	0.266	5.43E-02	1.00E+00	7.74E-02	q11.1			
12	rs1230693	36528296	T	0.2857	0.4673	C	3.689	0.456	5.48E-02	1.00E+00	7.74E-02	q12			
8	rs1009982	47711911	G	0.08333	0.254	A	3.677	0.267	5.52E-02	1.00E+00	7.74E-02	q11.1			
8	rs1205636	47588382	A	0.1071	0.2676	G	3.664	0.3285	5.56E-02	1.00E+00	7.74E-02	q11.1			
8	rs7293734	47457400	T	0.1071	0.2662	C	3.613	0.3308	5.73E-02	1.00E+00	7.83E-02	q11.1			
8	rs9650442	47224322	T	0.08333	0.2493	C	3.523	0.2737	6.05E-02	1.00E+00	8.11E-02	q11.1			
8	rs359813	47339255	A	0.08333	0.2472	G	3.452	0.2769	6.32E-02	1.00E+00	8.14E-02	q11.1			
6	rs3004002	77426060	T	0.2143	0.3847	C	3.419	0.4362	6.44E-02	1.00E+00	8.14E-02	q14.1	G|A	RP11-354K	5upstream
8	rs2354491	47901302	C	0.1071	0.2604	T	3.402	0.3409	6.51E-02	1.00E+00	8.14E-02	q11.1	G|A	RP11-101E	3downstream
4	rs7664705	1.79E+08	T	0.5	0.335	C	3.395	1.985	6.54E-02	1.00E+00	8.14E-02	q34.3	C|T	RP11-130F	non-coding intronic
8	rs1916188	47915603	A	0.1071	0.2581	C	3.321	0.3449	6.84E-02	1.00E+00	8.30E-02	q11.1			
8	rs990760	47859016	T	0.1071	0.257	C	3.28	0.347	7.01E-02	1.00E+00	8.30E-02	q11.1	A|G	MTND1P7,	non-coding intronic
8	rs406800	88604177	G	0.3214	0.493	A	3.28	0.4871	7.01E-02	1.00E+00	8.30E-02	q21.3	C|T	CNBD1	intronic
5	rs253365	16850415	C	0.2692	0.4372	T	2.968	0.4742	8.49E-02	1.00E+00	9.89E-02	p15.1	C|T	MYO10	intronic

Perseverative Behavior (Sameness)

Q1 (N = 15)	CHR	SNP	BP	A1	F_A	F_U	A2	CHISQ	OR	UNADJ	BONF	FDR_BH	Band	Alleles	Gene(s)	Role
	11	rs4930028	2889641	A	0.6	0.3487	C	8.271	2.801	4.03E-03	1.61E-02	1.38E-02	11p15.4	A/C	SLC22A18	Intron
	5	rs324454	55238308	A	0.6667	0.4222	G	7.299	2.737	6.90E-03	2.76E-02	1.38E-02	5q11.2	A/G	IL31RA	Intron
	11	rs7926034	90154444	A	0.6667	0.4407	G	6.172	2.538	1.30E-02	5.19E-02	1.73E-02	11q14.3	A/G		

Q4 (N = 18)	CHR	SNP	BP	A1	F_A	F_U	A2	CHISQ	OR	UNADJ	BONF	FDR_BH	Band	Alleles	Gene(s)	Role
	12	rs2058604	12841126	A	0.6944	0.3751	C	15.51	3.787	8.19E-05	3.28E-04	3.28E-04	12p13.1	A/C	APOLD1	Downstream
	11	rs4930028	2889641	A	0.08333	0.3487	C	11.11	0.1698	8.58E-04	3.43E-03	1.72E-03	11p15.4	A/C	SLC22A18	Intron
	11	rs7926034	90154444	A	0.25	0.4407	G	5.276	0.423	2.16E-02	8.65E-02	2.88E-02	11q14.3	A/G		
	5	rs324454	55238308	A	0.25	0.4222	G	4.346	0.4562	3.71E-02	1.48E-01	3.71E-02	5q11.2	A/G	IL31RA	Intron
