# Supplementary material for: ASD Phenotype—Genotype Associations in Concordant and Discordant Monozygotic and Dizygotic Twins Stratified by Severity of Autistic Traits
Source: Int J Mol Sci. 2019 Aug 3;20(15):3804. doi: 10.3390/ijms20153804 (PMC6696087; doi:10.3390/ijms20153804)
Supplement: Supplementary file 1 [file ijms-20-03804-s001.zip › Supplementary Materials/Table S8. Case-control by quartile analysis of dA.MZ twins.pdf]

Summary of case-control by quartile analyses of dA.MZ twins

Language

Q1 (N = 4)	CHR	SNP	BP	A1	F_A	F_U	A2	CHISQ	OR	UNADJ P	BONF	FDR_BH	Band	Alleles	Gene(s)	Role
	17	rs756785	27836958	A	0.875	0.4267	G	6.56	9.406	0.01043	0.09389	0.09389	17q11.2	A/G	CDK5R1	Promoter

Q4 (N = 7)	CHR	SNP	BP	A1	F_A	F_U	A2	CHISQ	OR	UNADJ P	BONF	FDR_BH	Band	Alleles	Gene(s)	Role
	7	rs1027519	85436760	A	0.5	0.1249	G	17.83	7.003	2.42E-05	0.000218	0.000119	7q21.11	A/G		
	7	rs7785694	85431498	A	0.5	0.1257	G	17.66	6.954	2.64E-05	0.000237	0.000119	7q21.11	A/G		
	11	rs1809063	131636242	C	0.9286	0.4592	T	12.38	15.31	0.000434	0.003904	0.001301	11q25	C/T	NTM	Intron
	13	rs9594670	41529489	C	0.5	0.1803	T	8.246	4.545	0.004085	0.03676	0.009191	13q14.11	C/T	DGKH	Intron
	17	rs756785	27836958	A	0.07143	0.4267	G	7.206	0.1034	0.007267	0.0654	0.01308	17q11.2	A/G	CDK5R1	Promoter
	1	rs2774285	114443208	T	0.5	0.2328	C	5.568	3.296	0.01829	0.1647	0.02744	1p13.2	C/T	SYT6	Intron (boundary)
	9	rs2809445	127988530	T	0.5	0.2489	C	4.701	3.018	0.03015	0.2714	0.03877	9q33.3	C/T		

Nonverbal

Q1 (N = 5)	CHR	SNP	BP	A1	F_A	F_U	A2	CHISQ	OR	UNADJ P	BONF	FDR_BH	Band	Alleles	Gene(s)	Role
	10	rs1836470	45099325	C	0	0.4107	T	6.959	0	0.008338	0.02502	0.02502	10q11.21	C/T		
	7	rs6462639	36019937	G	0	0.3302	A	4.925	0	0.02648	0.07943	0.03972	7p14.2	A/G		
	17	rs1108018	27707111	A	0.1	0.4042	G	3.836	0.1638	0.05015	0.1504	0.05015	17q11.2	A/G	ZNF207	Intron

Q4 (N = 6)	CHR	SNP	BP	A1	F_A	F_U	A2	CHISQ	OR	UNADJ	BONF	FDR_BH	Band	Alleles	Gene(s)	Role
	10	rs1836470	45099325	C	0.9167	0.4107	T	12.65	15.78	0.000376	0.001127	0.001127	10q11.21	C/T		
	17	rs1108018	27707111	A	0.8333	0.4042	G	9.146	7.37	0.002492	0.007476	0.003738	17q11.2	A/G	ZNF207	Intron
	7	rs6462639	36019937	G	0.6667	0.3302	A	6.12	4.057	0.01336	0.04009	0.01336	7p14.2	A/G		

Play

Q1 (N = 3)	CHR	SNP	BP	A1	F_A	F_U	A2	CHISQ	OR	UNADJ P	BONF	FDR_BH	Band	Alleles	Gene(s)	Role
	2	rs355810	165393892	C	1	0.3692	T	10.23	NA	0.00138	0.002761	0.002207	2q24.3	C/T	COBLL1	Intron
	18	rs8088744	64685792	T	1	0.3899	G	9.369	NA	0.002207	0.004414	0.002207	18q22.1	G/T	CCDC102B	Intron

Q4 (N = 5)	CHR	SNP	BP	A1	F_A	F_U	A2	CHISQ	OR	UNADJ	BONF	FDR_BH	Band	Alleles	Gene(s)	Role
	2	rs355810	165393892	C	0	0.3692	T	5.845	0	0.01562	0.03125	0.03125	2q24.3	C/T	COBLL1	Intron

Social

Q1 (N = 5)	CHR	SNP	BP	A1	F_A	F_U	A2	CHISQ	OR	UNADJ P	BONF	FDR_BH	Band	Alleles	Gene(s)	Role
	23	rs2097402	133945220	A	1	0.3131	G	13.12	NA	0.000293	0.003511	0.003511	Xq26.3	A/G	LOC64453	Promoter
	5	rs2287672	79125284	A	0.7	0.2818	G	8.609	5.947	0.003346	0.04015	0.01355	5q14.1	A/G	CMYA5	Intron (boundary)
	19	rs3826861	50748460	G	0.8	0.3557	A	8.586	7.245	0.003388	0.04066	0.01355	19q13.32	A/G	OPA3	Intron
	10	rs7070581	126159363	A	0.5	0.1763	G	7.181	4.673	0.007367	0.08841	0.0221	10q26.13	A/G	LHPP	Intron
	19	rs1264226	50755007	G	0.8	0.41	A	6.27	5.756	0.01228	0.1473	0.02947	19q13.32	A/G	OPA3	Intron
	4	rs1172317	187648291	A	0	0.3301	C	4.923	0	0.0265	0.318	0.053	4q35.2	A/C		
	3	rs4856450	83388138	A	0.1	0.4193	G	4.181	0.1539	0.04088	0.4906	0.07008	3p12.2	A/G		
	10	rs521331	8264856	T	0.5	0.2447	C	3.515	3.087	0.06082	0.7298	0.09123	10p14	C/T		

Q4 (N = 5)	CHR	SNP	BP	A1	F_A	F_U	A2	CHISQ	OR	UNADJ P	BONF	FDR_BH	Band	Alleles	Gene(s)	Role
	3	rs4856450	83388138	A	0.9	0.4193	G	9.463	12.46	0.002097	0.02516	0.02516	3p12.2	A/G		
	23	rs5962427	103575337	T	1	0.4583	C	7.077	NA	0.007808	0.0937	0.03372	Xq22.2	C/T		
	19	rs1264226	50755007	G	0	0.41	A	6.94	0	0.008429	0.1011	0.03372	19q13.32	A/G	OPA3	Intron
	19	rs3826861	50748460	G	0	0.3557	A	5.515	0	0.01886	0.2263	0.05657	19q13.32	A/G	OPA3	Intron
	5	rs2287672	79125284	A	0	0.2818	G	3.92	0	0.04771	0.5725	0.08638	5q14.1	A/G	CMYA5	Intron (boundary)
	12	rs1050586	20228698	A	0	0.2669	G	3.639	0	0.05645	0.6774	0.08638	12p12.2	A/G		
	1	rs1110264	113872742	C	0	0.26	T	3.51	0	0.061	0.732	0.08638	1p13.2	C/T	MAGI3	Intron
	1	rs1110262	113761409	A	0	0.2572	G	3.46	0	0.06287	0.7545	0.08638	1p13.2	A/G	MAGI3	Intron
	4	rs1172317	187648291	A	0.6	0.3301	C	3.284	3.044	0.06995	0.8394	0.08638	4q35.2	A/C		
	10	rs521331	8264856	T	0	0.2447	C	3.237	0	0.07198	0.8638	0.08638	10p14	C/T		

Perseverative Behavior (Sameness)

Q1 (N = 6)	CHR	SNP	BP	A1	F_A	F_U	A2	CHISQ	OR	UNADJ	BONF	FDR_BH	Band	Alleles	Gene(s)	Role
	10	rs2150768	34098174	C	0	0.3277	T	5.841	0	0.01566	0.03131	0.03131	10p11.22	C/T		

Q4 (N = 5)	CHR	SNP	BP	A1	F_A	F_U	A2	CHISQ	OR	UNADJ P	BONF	FDR_BH	Band	Alleles	Gene(s)	Role
	15	rs1051876	52322448	T	0.6	0.1701	G	13.01	7.317	0.00031	0.00062	0.00062	15q21.3	G/T	UNC13C	Intron
	10	rs2150768	34098174	C	0.6	0.3277	T	3.357	3.078	0.06692	0.1338	0.06692	10p11.22	C/T		
