# Supplementary material for: ASD Phenotype—Genotype Associations in Concordant and Discordant Monozygotic and Dizygotic Twins Stratified by Severity of Autistic Traits
Source: Int J Mol Sci. 2019 Aug 3;20(15):3804. doi: 10.3390/ijms20153804 (PMC6696087; doi:10.3390/ijms20153804)
Supplement: Supplementary file 1 [file ijms-20-03804-s001.zip › Supplementary Materials/Table S9. Case-control by quartile analysis of dNA.MZ twins.pdf]

Summary of case-control by quartile analysis of dNA.MZ twins (using genotypes of dA.MZ as proxy)

Language

Q4 (N = 6)	CHR	SNP	BP	A1	F_A	F_U	A2	CHISQ	OR	UNADJ	BONF	FDR_BH	Band	Alleles	Gene(s)	Role
	7	rs1553841	47510767	T	0.417	0.052	C	31.56	12.94	1.94E-08	5.04E-07	5.04E-07	7p12.3	C|T	TNS3	Intron
	2	rs6742060	238234971	G	0.500	0.105	A	19.71	8.52	9.00E-06	2.34E-04	1.17E-04	2q37.3	A|G	LRRFIP1	Intron
	5	rs3896348	18204313	G	0.667	0.211	A	14.93	7.50	1.11E-04	2.90E-03	8.54E-04	5p15.1			
	2	rs3791289	134827909	C	0.750	0.263	T	14.62	8.42	1.31E-04	3.42E-03	8.54E-04	2q21.3	C|T	MGAT5	Intron
	14	rs1015166	33007535	C	0.583	0.176	T	13.61	6.55	2.25E-04	5.85E-03	1.17E-03	14q13.1	T|C	NPAS3	Intron
	6	rs4443522	129211626	G	0.917	0.452	A	10.42	13.33	1.25E-03	3.24E-02	5.40E-03	6q22.33			
	6	rs4487603	129211602	T	0.917	0.463	C	9.931	12.79	1.63E-03	4.23E-02	6.04E-03	6q22.33			
	12	rs1996749	102245143	A	0.833	0.398	G	9.446	7.55	2.12E-03	5.50E-02	6.88E-03	12q23.2	G|A	C12orf42	Intron
	1	rs3766793	53363218	C	0.500	0.173	T	8.937	4.79	2.79E-03	7.26E-02	8.07E-03	1p32.3	T|C	SLC1A7	Intron
	2	rs1049023	50391831	T	0.417	0.136	C	8.002	4.54	4.67E-03	1.22E-01	1.22E-02	2p16.3	G|A	NRXN1	Intron
	1	rs783036	66603958	T	0.083	0.481	C	7.581	0.10	5.90E-03	1.53E-01	1.32E-02	1p31.3	G|A	PDE4B	coding
	8	rs2618806	2111558	C	0.500	0.189	T	7.527	4.29	6.08E-03	1.58E-01	1.32E-02	8p23.3			
	5	rs923963	62075399	G	0.000	0.351	T	6.481	0.00	1.09E-02	2.84E-01	2.18E-02	5q12.1			
	8	rs921693	131074193	T	0.583	0.263	C	6.334	3.93	1.18E-02	3.08E-01	2.20E-02	8q24.21	G|A	FAM49B	Intron
	8	rs1095650	131051089	A	0.600	0.256	C	6.179	4.36	1.29E-02	3.36E-01	2.24E-02	8q24.21	C|A	FAM49B	Intron
	12	rs1105788	123937926	A	0.417	0.159	C	5.925	3.78	1.49E-02	3.88E-01	2.43E-02	12q24.31			
	7	rs1023837	30940878	A	0.500	0.223	G	5.305	3.49	2.13E-02	5.53E-01	3.12E-02	7p14.3			
	3	rs4521229	181973610	A	0.500	0.223	G	5.277	3.48	2.16E-02	5.62E-01	3.12E-02	3q26.33	G|A	CCDC39	Intron
	11	rs1104153	7671750	C	0.000	0.281	T	4.68	0.00	3.05E-02	7.94E-01	4.18E-02	11p15.4	T|C	OVCH2	Intron
	5	rs1252157	18187934	G	0.500	0.239	A	4.47	3.18	3.45E-02	8.97E-01	4.49E-02	5p15.1			
	6	rs1252529	107246561	G	0.500	0.246	T	4.166	3.07	4.13E-02	1.00E+00	5.11E-02	6q21			
	15	rs1291526	30196358	C	0.417	0.201	T	3.448	2.84	6.33E-02	1.00E+00	7.49E-02	15q13.3	T|C	CHRNA7	Intron
	8	rs604302	37123727	A	0.417	0.209	G	3.126	2.71	7.70E-02	1.00E+00	8.71E-02	8p11.23			

Nonverbal

Q4 (N = 4)	CHR	SNP	BP	A1	F_A	F_U	A2	CHISQ	OR	UNADJ	BONF	FDR_BH	Band	Alleles	Gene(s)	Role
	11	rs7951632	110358395	A	0.625	0.074	G	34.75	20.72	3.75E-09	1.09E-07	1.09E-07	11q23.1			
	2	rs6745492	237172556	T	0.500	0.055	C	30.34	17.33	3.62E-08	1.05E-06	5.25E-07	2q37.3			
	22	rs739140	36445502	G	0.625	0.106	A	22.68	14.13	1.91E-06	5.55E-05	1.85E-05	22q13.1	G|A	TRIOBP	Intron
	1	rs1683335	234358516	G	0.500	0.073	A	21.18	12.62	4.19E-06	1.21E-04	2.69E-05	1q42.3			
	3	rs7628531	72684645	G	0.625	0.112	A	20.93	13.18	4.75E-06	1.38E-04	2.69E-05	3p13			
	19	rs4808521	16646721	G	0.625	0.114	A	20.63	13.02	5.56E-06	1.61E-04	2.69E-05	19p13.11	A|G	TMEM38A	Intron
	3	rs2010479	127860189	C	0.500	0.080	T	18.93	11.47	1.36E-05	3.94E-04	4.01E-05	3q21.3	T|C	NUP210P1	5upstream
	6	rs1414743	142319462	T	0.625	0.121	C	18.88	12.06	1.39E-05	4.04E-04	4.01E-05	6q24.1	G|A	AK097143	Intron
	2	rs2072474	102005641	C	0.750	0.171	T	18.8	14.54	1.45E-05	4.21E-04	4.01E-05	2q11.2	A|G	IL1R2	Intron
	6	rs2256028	31487177	T	0.750	0.172	G	18.73	14.49	1.51E-05	4.38E-04	4.01E-05	6p21.33	C|A	MICA	Intron
	3	rs7628233	127878243	A	0.500	0.081	G	18.71	11.36	1.52E-05	4.41E-04	4.01E-05	3q21.3			
	1	rs1112006	211141648	T	0.625	0.125	C	18.2	11.69	1.98E-05	5.76E-04	4.52E-05	1q32.3			
	1	rs1284851	211139989	T	0.625	0.125	C	18.16	11.67	2.03E-05	5.88E-04	4.52E-05	1q32.3	G|A	FLVCR1	3downstream
	5	rs42914	36771041	A	0.625	0.131	C	17.1	11.10	3.54E-05	1.03E-03	7.33E-05	5p13.2			
	12	rs1077798	98353626	T	0.750	0.194	C	15.79	12.50	7.09E-05	2.06E-03	1.37E-04	12q23.1	C|T	ANKS1B	Intron
	3	rs3872649	245521	C	0.625	0.140	T	15.59	10.27	7.85E-05	2.28E-03	1.42E-04	3p26.3	G|A	CHL1	Intron
	20	rs2300216	3894057	C	0.625	0.142	T	15.2	10.06	9.65E-05	2.80E-03	1.65E-04	20p13	A|G	RNF24	Intron
	17	rs1107942	56810004	A	0.500	0.096	G	14.92	9.42	1.12E-04	3.24E-03	1.80E-04	17q23.2	A|G	BCAS3	Intron
	20	rs6062894	61425120	C	0.500	0.107	T	12.86	8.36	3.36E-04	9.73E-03	5.12E-04	20q13.33	C|T	COL20A1	Intron
	4	rs737601	9762331	G	0.500	0.111	A	12.24	8.04	4.68E-04	1.36E-02	6.79E-04	4p16.1			
	6	rs663614	87491867	C	0.500	0.119	A	11.05	7.43	8.86E-04	2.57E-02	1.22E-03	6q14.3			
	4	rs1093763	5428143	C	0.625	0.184	T	10.31	7.39	1.32E-03	3.84E-02	1.75E-03	4p16.2	T|C	STK32B	Intron
	8	rs6471603	58517186	C	0.667	0.195	T	8.472	8.26	3.61E-03	1.05E-01	4.55E-03	8q12.1			
	11	rs1121543	114616921	A	0.625	0.221	G	7.578	5.89	5.91E-03	1.71E-01	7.14E-03	11q23.3	G|A	CADM1	Intron
	4	rs1001786	16203977	C	0.667	0.213	A	7.373	7.41	6.62E-03	1.92E-01	7.68E-03	4p15.32	A|C	LDB2	Intron
	19	rs7250000	56578734	A	0.625	0.270	G	5.083	4.50	2.42E-02	7.01E-01	2.70E-02	19q13.41	G|A	LIM2	Intron
	13	rs7320823	67575838	C	0.667	0.274	A	4.637	5.30	3.13E-02	9.08E-01	3.36E-02	13q21.33			
	6	rs2185503	8294510	C	0.625	0.315	T	3.545	3.62	5.97E-02	1.00E+00	6.19E-02	6p24.3			
	10	rs1931901	7695358	A	0.625	0.339	G	2.908	3.25	8.81E-02	1.00E+00	8.81E-02	10p14	G|A	ITIH5	Intron

Play

Q1 (N = 5)	CHR	SNP	BP	A1	F_A	F_U	A2	CHISQ	OR	UNADJ	BONF	FDR_BH	Band	Alleles	Gene(s)	Role
	3	rs333327	125813434	A	0.000	0.459	G	8.475	0.00	3.60E-03	2.52E-02	2.52E-02	3q21.2	G|A	KALRN	Intron
	11	rs713065	86335168	A	0.000	0.387	G	6.307	0.00	1.20E-02	8.42E-02	2.81E-02	11q14.2	A|G	FZD4 (PRS	3utr (intron)
	1	rs485874	18945513	A	0.800	0.427	G	5.687	5.38	1.71E-02	1.20E-01	2.81E-02	1p36.13	A|G	PAX7	3utr
	4	rs2725769	105975618	A	0.000	0.354	G	5.48	0.00	1.92E-02	1.35E-01	2.81E-02	4q24	G|A	RP11-556I14.2	
	4	rs2725771	105978188	A	0.000	0.351	C	5.408	0.00	2.00E-02	1.40E-01	2.81E-02	4q24			
	10	rs2505956	27225292	T	0.000	0.275	C	3.78	0.00	5.19E-02	3.63E-01	6.05E-02	10p12.1			

Q4 (N = 5)	CHR	SNP	BP	A1	F_A	F_U	A2	CHISQ	OR	UNADJ	BONF	FDR_BH	Band	Alleles	Gene(s)	Role
	12	rs4523751	89842084	G	0.800	0.226	A	18.77	13.73	1.47E-05	1.03E-04	1.03E-04	12q21.33	G|A	LINC00615	Intron
	10	rs2505956	27225292	T	0.875	0.275	C	14.43	18.50	1.45E-04	1.02E-03	5.09E-04	10p12.1			
	3	rs333327	125813434	A	0.900	0.459	G	7.807	10.60	5.20E-03	3.64E-02	1.04E-02	3q21.2	G|A	KALRN	Intron
	1	rs485874	18945513	A	0.000	0.427	G	7.427	0.00	6.43E-03	4.50E-02	1.04E-02	1p36.13	A|G	PAX7	3utr
	11	rs713065	86335168	A	0.800	0.387	G	7.167	6.33	7.43E-03	5.20E-02	1.04E-02	11q14.2	A|G	FZD4 (PRS	3utr (intron)
	4	rs2725771	105978188	A	0.700	0.351	C	5.321	4.31	2.11E-02	1.48E-01	2.25E-02	4q24			
	4	rs2725769	105975618	A	0.700	0.354	G	5.21	4.25	2.25E-02	1.57E-01	2.25E-02	4q24	G|A	RP11-556I14.2	

Social

Q4 (N = 4)	CHR	SNP	BP	A1	F_A	F_U	A2	CHISQ	OR	UNADJ	BONF	FDR_BH	Band	Alleles	Gene(s)	Role
	20	rs1698945	39011296	G	0.500	0.061	A	26.4	15.30	2.78E-07	1.11E-05	1.11E-05	20q12			
	9	rs2007744	78044132	A	0.750	0.144	C	23.62	17.81	1.17E-06	4.69E-05	2.35E-05	9q21.13	T|G	PCSK5	intronic
	7	rs1095384	117174344	T	0.500	0.073	C	21.24	12.65	4.05E-06	1.62E-04	4.48E-05	7q31.2	C|T	CTTNBP2	intronic
	7	rs1176781	117206428	C	0.500	0.074	A	21.05	12.55	4.48E-06	1.79E-04	4.48E-05	7q31.31	A|C	CTTNBP2	intronic
	3	rs3907388	2009933	C	0.500	0.076	T	20.16	12.10	7.13E-06	2.85E-04	5.71E-05	3p26.3			
	7	rs1034784	82062116	C	0.500	0.080	T	18.93	11.47	1.36E-05	5.43E-04	9.05E-05	7q21.11			
	13	rs1421296	105057211	A	0.500	0.082	C	18.33	11.16	1.86E-05	7.43E-04	1.06E-04	13q33.2			
	7	rs1025221	53387975	A	0.500	0.084	G	17.86	10.92	2.38E-05	9.53E-04	1.19E-04	7p12.1			
	6	rs6902999	105866239	T	0.500	0.090	C	16.22	10.09	5.63E-05	2.25E-03	2.50E-04	6q21	C|T	PREP	intronic
	14	rs1013103	32319832	A	0.500	0.091	G	15.93	9.93	6.56E-05	2.62E-03	2.62E-04	14q12	G|A	AKAP6	intronic
	3	rs717939	18601746	G	0.875	0.268	A	15	19.17	1.07E-04	4.29E-03	3.90E-04	3p24.3	T|C	AC144521.	intronic
	8	rs1044783	416685	G	0.625	0.170	A	11.67	8.13	6.36E-04	2.54E-02	2.12E-03	8p23.3	A|G	AK056623	3utr
	11	rs1083043	89541802	G	0.500	0.118	A	11.16	7.48	8.35E-04	3.34E-02	2.57E-03	11q14.3	A|G	NAALAD2	coding
	16	rs2911174	62214791	C	0.875	0.329	T	10.77	14.28	1.03E-03	4.13E-02	2.95E-03	16q21			
	10	rs771874	36266482	A	0.500	0.106	G	9.826	8.47	1.72E-03	6.88E-02	4.32E-03	10p11.21			
	12	rs1440092	95273987	T	0.500	0.130	C	9.649	6.70	1.89E-03	7.58E-02	4.32E-03	12q23.1	A|G	CDK17	intronic
	12	rs1050707	95280908	T	0.500	0.130	C	9.644	6.70	1.90E-03	7.60E-02	4.32E-03	12q23.1	T|C	CDK17	intronic
	12	rs7976881	95315077	G	0.500	0.130	A	9.603	6.68	1.94E-03	7.77E-02	4.32E-03	12q23.1	G|A	CDK17	intronic
	19	rs2075604	1169523	T	0.500	0.132	G	9.417	6.59	2.15E-03	8.60E-02	4.50E-03	19p13.3	G|T	STK11	intronic
	2	rs6717613	299480	A	0.875	0.357	G	9.333	12.62	2.25E-03	9.00E-02	4.50E-03	2p25.3	G|A	AC079779.	5upstream (intronic)
	7	rs2941543	47492265	A	0.625	0.206	G	8.582	6.44	3.40E-03	1.36E-01	6.47E-03	7p12.3	A|G	TNS3	intronic
	12	rs4608180	95165979	G	0.500	0.142	A	8.4	6.06	3.75E-03	1.50E-01	6.82E-03	q23.1	G|A	ELK3	intronic
	6	rs1217953	18572627	T	0.500	0.143	G	8.242	5.97	4.09E-03	1.64E-01	7.12E-03	NA			
	1	rs1329465	99155952	C	0.500	0.159	T	6.963	5.31	8.32E-03	3.33E-01	1.39E-02	1p21.3	T|C	LPPR5	intronic
	8	rs1198768	123131375	A	0.500	0.170	G	6.12	4.87	1.34E-02	5.35E-01	2.14E-02	8q24.13	G|A	BC052578	intronic
	4	rs1109763	99963878	C	0.500	0.177	T	5.691	4.64	1.71E-02	6.82E-01	2.56E-02	4q23			
	3	rs2371121	27825729	G	0.500	0.178	T	5.641	4.62	1.76E-02	7.02E-01	2.56E-02	3p24.1	G|T	AC098973.	intronic
	3	rs2618111	27886915	A	0.500	0.179	G	5.605	4.60	1.79E-02	7.17E-01	2.56E-02	3p24.1	A|G	AC098973.	intronic
	6	rs9501714	135248	G	0.500	0.180	A	5.54	4.56	1.86E-02	7.44E-01	2.56E-02	6p25.3	A|G	RP3-416J7	intronic
	18	rs663830	10594362	C	0.500	0.213	T	3.934	3.70	4.73E-02	1.00E+00	6.31E-02	18p11.22	T|C	RP11-883A	intronic
	14	rs1599965	94944160	G	0.000	0.318	A	3.732	0.00	5.34E-02	1.00E+00	6.89E-02	14q32.13	T|C	LINC00341	3utr
	4	rs4401453	99993206	C	0.500	0.222	T	3.57	3.51	5.88E-02	1.00E+00	7.20E-02	4q23			
	17	rs7208605	11222570	G	0.500	0.222	A	3.554	3.50	5.94E-02	1.00E+00	7.20E-02	7p12	A|G	SHISA6	intronic
	6	rs1408267	18530528	G	0.500	0.228	A	3.347	3.38	6.73E-02	1.00E+00	7.80E-02	6p22.3	T|C	RNF144B	intronic
	17	rs8068118	11256344	A	0.500	0.229	G	3.312	3.36	6.88E-02	1.00E+00	7.80E-02	17p12	G|A	SHISA6	intronic
	9	rs328012	107077538	G	0.500	0.230	T	3.277	3.35	7.02E-02	1.00E+00	7.80E-02	9q31.1	A|C	SLC44A1	intronic
	6	rs567698	18562822	T	0.500	0.234	G	3.139	3.27	7.64E-02	1.00E+00	8.26E-02	6p22.3	T|G	RNF144B	intronic
	2	rs1684465	196653630	T	0.500	0.240	C	2.964	3.17	8.51E-02	1.00E+00	8.80E-02	2q32.3			
	2	rs4395260	196675582	A	0.500	0.240	G	2.952	3.16	8.58E-02	1.00E+00	8.80E-02	2q32.3			

Perseverative Behaviors

Q1 (N = 7)	CHR	SNP	BP	A1	F_A	F_U	A2	CHISQ	OR	UNADJ	BONF	FDR_BH	Band	Alleles	Gene(s)	Role
	23	rs1974522	3248733	G	0.000	0.443	A	7.939	0.00	4.84E-03	7.74E-02	4.73E-02	23p22.33	G|A|C	MXRA5	Coding
	13	rs1050800	92952411	G	0.857	0.498	A	7.205	6.05	7.27E-03	1.16E-01	4.73E-02	13q31.3	A/G	GPC6	Intron
	2	rs4641966	73444314	A	0.000	0.299	G	5.972	0.00	1.45E-02	2.33E-01	4.73E-02	2p13.1			
	2	rs6546824	73440839	A	0.000	0.298	G	5.945	0.00	1.48E-02	2.36E-01	4.73E-02	2p13.1			
	2	rs1019151	73411911	A	0.000	0.289	G	5.693	0.00	1.70E-02	2.73E-01	4.73E-02	2p13.1			
	8	rs7828540	72258791	A	0.000	0.287	G	5.621	0.00	1.78E-02	2.84E-01	4.73E-02	8q13.3	A|G	RP11-326E	Intron
	11	rs1089161	113381063	T	0.071	0.346	C	4.645	0.15	3.11E-02	4.98E-01	6.92E-02	11q23.2			
	16	rs410059	78212573	C	0.214	0.497	T	4.464	0.28	3.46E-02	5.54E-01	6.92E-02	16q23.2			
	10	rs1074956	89882080	G	0.143	0.412	A	4.186	0.24	4.08E-02	6.52E-01	7.25E-02	10q23.31	A|G	RNLS	3utr
	11	rs4938066	113390622	G	0.071	0.321	A	3.993	0.16	4.57E-02	7.31E-01	7.31E-02	11q23.2			
	11	rs7395845	70920892	C	0.071	0.299	A	3.449	0.18	6.33E-02	1.00E+00	9.21E-02	11q13.4			
	12	rs1073593	65340149	T	0.000	0.190	C	3.273	0.00	7.04E-02	1.00E+00	9.39E-02	12q14.3	T|C	GRIP1	Intron

Q4 (N = 6)	CHR	SNP	BP	A1	F_A	F_U	A2	CHISQ	OR	UNADJ	BONF	FDR_BH	Band	Alleles	Gene(s)	Role
	9	rs7031476	15047624	A	0.667	0.168	G	21.1	9.88	4.35E-06	6.96E-05	6.96E-05	9p22.3	G|A	AK127963	Intron
	11	rs2509094	95360556	A	0.500	0.126	G	15.11	6.94	1.01E-04	1.62E-03	8.11E-04	11q21	G|A	MAML2	Intron
	10	rs1074956	89882080	G	0.917	0.412	A	12.55	15.68	3.95E-04	6.32E-03	1.40E-03	10q23.31	A|G	RNLS	3utr
	8	rs7828540	72258791	A	0.750	0.287	G	12.53	7.46	4.00E-04	6.39E-03	1.40E-03	8q13.3	A|G	RP11-326E	Intron
	16	rs410059	78212573	C	1.000	0.497	T	12.11	NA	5.01E-04	8.02E-03	1.40E-03	16q23.2			
	12	rs1073593	65340149	T	0.583	0.190	C	12.03	5.99	5.23E-04	8.37E-03	1.40E-03	12q14.3	T|C	GRIP1	Intron
	11	rs1089161	113381063	T	0.750	0.346	C	8.642	5.68	3.28E-03	5.26E-02	7.32E-03	11q23.2			
	8	rs184487	26404795	C	0.583	0.232	A	8.289	4.64	3.99E-03	6.38E-02	7.32E-03	8p21.2	C|A	BNIP3L	Intron
	13	rs1050800	92952411	G	0.083	0.498	A	8.231	0.09	4.12E-03	6.59E-02	7.32E-03	13q31.3	A|G	GPC6	Intron
	8	rs189025	26393432	A	0.583	0.241	G	7.64	4.41	5.71E-03	9.14E-02	8.85E-03	8p21.2		BNIP3L	Intron
	23	rs1974522	3248733	G	1.000	0.443	A	7.526	NA	6.08E-03	9.73E-02	8.85E-03	23p22.33	G|A|C	MXRA5	Coding
	11	rs4938066	113390622	G	0.667	0.321	A	6.556	4.23	1.05E-02	1.67E-01	1.39E-02	11q23.2			
	11	rs7395845	70920892	C	0.583	0.299	A	4.619	3.29	3.16E-02	5.06E-01	3.89E-02	11q13.4			
